# Extreme cranial asymmetry in extinct benthic feeding sperm whales

**DOI:** 10.1038/s42003-026-11074-2

**Published:** 2026-11-09

**Authors:** Aldo Benites-Palomino, Marcelo R. Sánchez-Villagra, Florencia Paolucci, Jorge Vélez-Juarbe, Alberto Collareta, Apolline Alfsen, Alton Dooley, Dan Omura, Mario Urbina, Rodolfo Salas-Gismondi, Gabriel Aguirre-Fernández

**Affiliations:** 1 GeoZentrum Nordbayern, Friedrich-Alexander-Universität Erlangen-Nürnberg, Erlangen, Germany; 2 Department of Paleontology, University of Zurich, Zürich, Switzerland; 3 Departamento de Paleontología de Vertebrados, Museo de Historia Natural-Universidad Nacional Mayor de San Marcos, Lima, Peru; 4 División Paleontología Vertebrados, Unidades de Investigación Anexo Museo, Facultad de Ciencias Naturales y Museo, UNLP, CONICET, La Plata, Argentina; 5 Department of Mammalogy, Natural History Museum of Los Angeles County, Los Angeles, CA USA; 6 Dipartimento di Scienze della Terra, Università di Pisa, Pisa, Italy; 7 Department of Evolutionary Morphology, Museum für Naturkunde, Leibniz-Institut für Evolutions- und Biodiversitätsforschung, Berlin, Germany; 8 Western Science Center, Hemet, CA USA; 9 Centro de Desarrollo para la Educación Lima Nikko, Asociación Peruano Japonesa, Lima, Perú; 10 Facultad de Ciencias y Filosofía/ Laboratorios de Investigación y Desarrollo, Universidad Peruana Cayetano Heredia, Lima, Peru

**Keywords:** Palaeontology, Taxonomy

## Abstract

The fossil record provides critical insights into morphological changes beyond those observable in extant species, revealing evolutionary novelties. Among mammals, toothed cetaceans are notable for pronounced cranial asymmetry associated with sound-emitting forehead structures. Pygmy sperm whales (Kogiidae),display some of the most extreme cranial asymmetries known. Here, we describe *Scaphokogia huachafo* sp. nov., a new pygmy sperm whale species from the Late Miocene Pisco Formation, Peru. This species is characterised by extreme craniofacial asymmetry that extends beyond the external bony nares to the surrounding cranial structures. Geometric morphometric and symmetry analyses centred on *S. huachafo* indicate that this genus exhibits the most extreme facial asymmetry documented among cetaceans, and potentially among mammals. Distinct morphological traits include a robust, ventrally oriented, tubular rostrum composed of dense pachyostotic bone and reinforced by ossified cartilage. Morphological reconstruction of the atlanto-occipital joint in closely related congeners suggest enhanced ventral neck flexibility in *Scaphokogia*, which in turn is consistent with a benthic foraging behaviour. This extreme cranial morphology likely represents a unique adaptation with no direct modern analogues – one that greatly expands the known diversity of cetacean skull architecture. *S. huachafo* demonstrably occupied extinct ecological niches, revealing pivotal benthic foraging shifts during the Late Miocene.

## Abstract (web summary)

A new Miocene pygmy sperm whale from Peru exhibits unparalleled cranial asymmetry and specialized benthic-feeding adaptations, greatly expanding the diversity of cetacean skull architecture.

## Introduction

Cranial asymmetry in vertebrates is predominantly associated with pathology or malformations^1,2^. However, in some cases, asymmetry emerges as an evolutionary innovation closely linked to the development of specific ecological traits. The most well-known example is found in flatfishes (Pleuronectiformes), where both eyes are located on one side of the head^3^. Among tetrapods, some owl species (Strigiformes) exhibit asymmetric skulls, which enhance sensory functions by improving auditory capabilities due to the differential positioning of their ears^4,5^, thereby increasing hunting efficiency during crepuscular hours. In mammals, cetaceans provide the most notable example of asymmetry. This condition is associated with adaptations to underwater life, such as directional hearing in archaeocetes^6,7^ (early-diverging cetaceans) and high-frequency sound production and echolocation in Odontoceti (toothed cetaceans), which enhance communication and prey acquisition skills^8^. Directional asymmetry, characterised by differences between the two sides of the body, reaches its most extreme manifestation in the skulls of odontocetes^9^. Sperm whales (Physeteroidea) exhibit this feature to an extraordinary degree^10,11^. Their skulls exhibit a pronounced leftward deviation of the right facial bones, including the premaxilla and nasal^12^. In addition, sperm whales have developed a dorsally excavated supracranial basin that houses an enlarged nasal complex consisting primarily of fatty forehead organs^13,14^

Directional cranial asymmetry and the development of the supracranial basin are both observed since the earliest stages of sperm whale evolution. These traits are evident even in some of the oldest known physeteroids, such as *Rhaphicetus*^15^, as well as in raptorial forms like *Acrophyseter* and *Zygophyseter*^16–18^. The fossil record further reveals substantial variations in the cranial patterning of extinct sperm whales, indicating that the development of nasal structures is species-specific within Physeteroidea^19^. This diversity in cranial shape disparity suggests that extinct sperm whales occupied a broader range of ecological niches mostly in pelagic waters during the Miocene, ranging from small piscivorous or teuthophagous taxa to large and giant apex predators. In contrast, extant physeteroids are specialised suction feeders, with diets primarily composed of cephalopods captured at great ocean depths^20–23^. Present-day sperm whales also differ markedly in body size and in the morphology of their supracranial basin. *Physeter macrocephalus*, the largest extant odontocete, is characterised by an undivided supracranial basin^13,14^, while species of *Kogia* (the only living member of the one-diverse family Kogiidae, or pygmy sperm whales) possess dorsally compartmentalised skulls with independent cavities housing multiple nasal organs^19–21^. Miocene fossils from Peru reveal a strikingly diverse stock of kogiid sperm whales, especially in the genus *Scaphokogia*, which stands out for its highly specialised craniofacial anatomy^22,23^.

Here, we describe a new pygmy sperm whale from the Late Miocene of Peru. This new species is characterised by a prominent, ventrally oriented tubular rostrum, constructed primarily of pachyostotic bone and ossified cartilage. Its cranial anatomy reveals an unprecedented degree of cranial asymmetry among cetaceans, as demonstrated by geometric morphometric analyses of craniofacial skull patterning. In addition, we reconstruct the range of motion of the atlanto-occipital joint connecting the skull and atlas in *Scaphokogia*, and propose an unusual feeding strategy for these distinctive sperm whales. Together, these results indicate that the new species exhibits a highly derived cranial morphology, suggesting a specialised benthic feeding ecology with no direct modern analogue among modern odontocetes. The species described herein expands the known limits of cranial architecture in cetaceans, and more broadly in mammals, and implies the existence of ecological niches once exploited by marine mammals that are no longer represented today.

### Systematic Palaeontology

Cetacea Brisson, 1792

Odontoceti Flower, 1867

Physeteroidea Gray, 1821

Kogiidae Gill, 1871

Scaphokogiinae Muizon, 1988

*Scaphokogia* Muizon, 1988

### Species included

*Scaphokogia cochlearis* Muizon, 1988, *Scaphokogia totajpe* Benites-Palomino et al. 2020, *Scaphokogia huachafo* sp. nov.

**Fig. 1:**
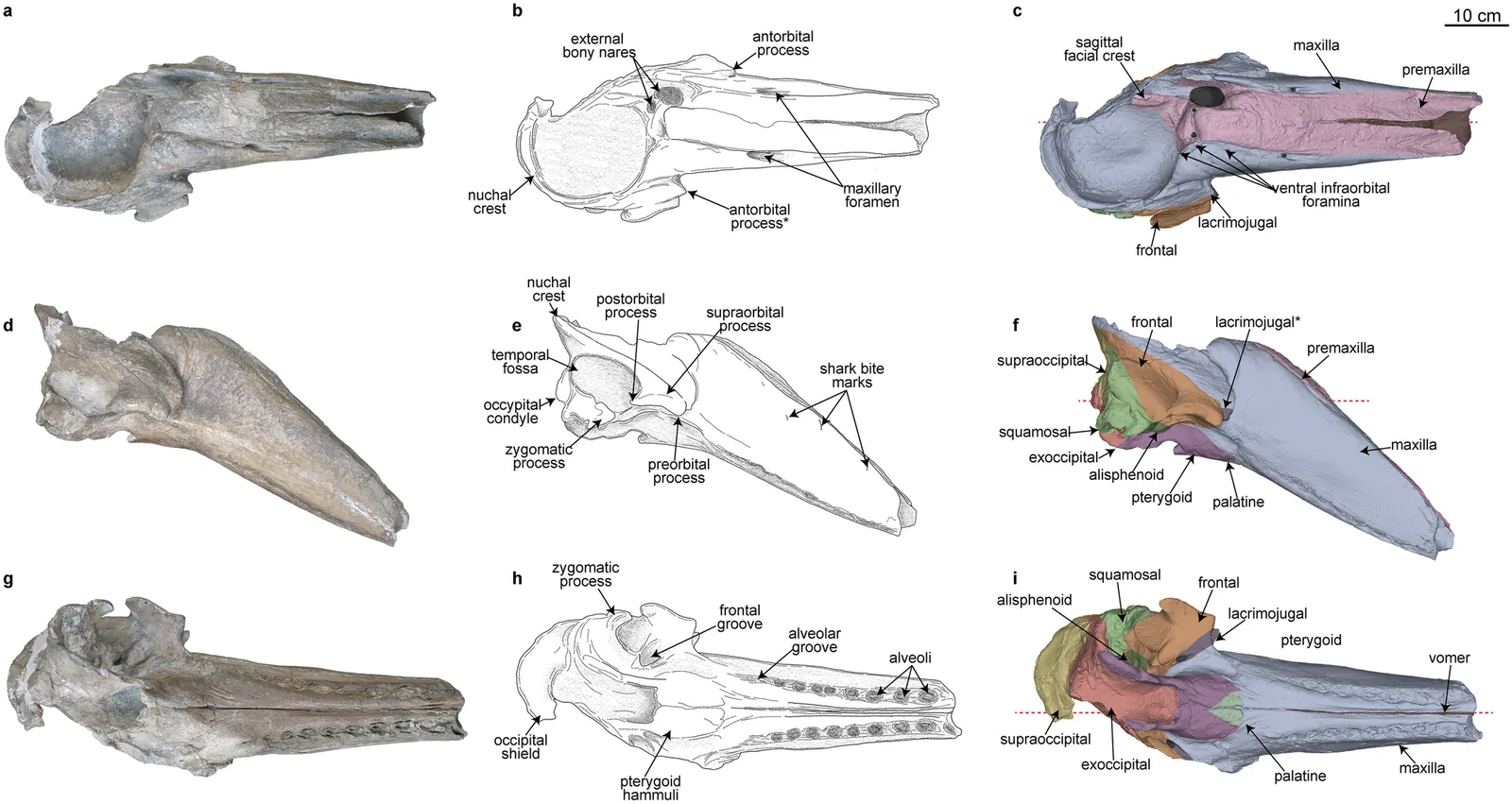
Holotype skull of *Scaphokogia huachafo* sp nov. MUSM 972 in dorsal (**a–c**), right lateral (**d–f**), and ventral (**g–i**) views, shown as the specimen (**a, d, g**), schematic drawings (**b, e, h**), and segmented 3D surface reconstructions (**c, f, i**). Dotted red lines indicate the main cranial axis.

*Scaphokogia huachafo* sp. nov.

(Figure 1; Supplementary Figs. 1–3; Supplementary Table 1)

### Etymology

The species epithet, “*huachafo*”, is a word commonly used in Peru to describe something “bizarre,” “odd,” or “extravagant,” highlighting the distinctive cranial features of the species.

### Holotype

The holotype skull of *Scaphokogia huachafo* gen. et sp. nov. MUSM 972(Departamento de Paleontologia de Vertebrados, Museo de Historia Natural) is mostly complete, except for the squamosal and lateralmost portion of the frontal on the left side of the cranium (Fig. 1; Supplementary Figs. 1–3). It corresponds to a fully-grown adult as indicated by the well-ossified cranial bones and the fully closed or ankylosed cranial sutures.

### Locality and age

MUSM 972 was collected in Aguada de Lomas, Sacaco basin (Arequipa, Peru). The fossil-bearing horizon was deposited between 6.3 and 5.6 Ma., during a shallow marine transgression^24,25^(Supplementary Notes 1).

### Phylogenetic framework

**Fig. 2:**
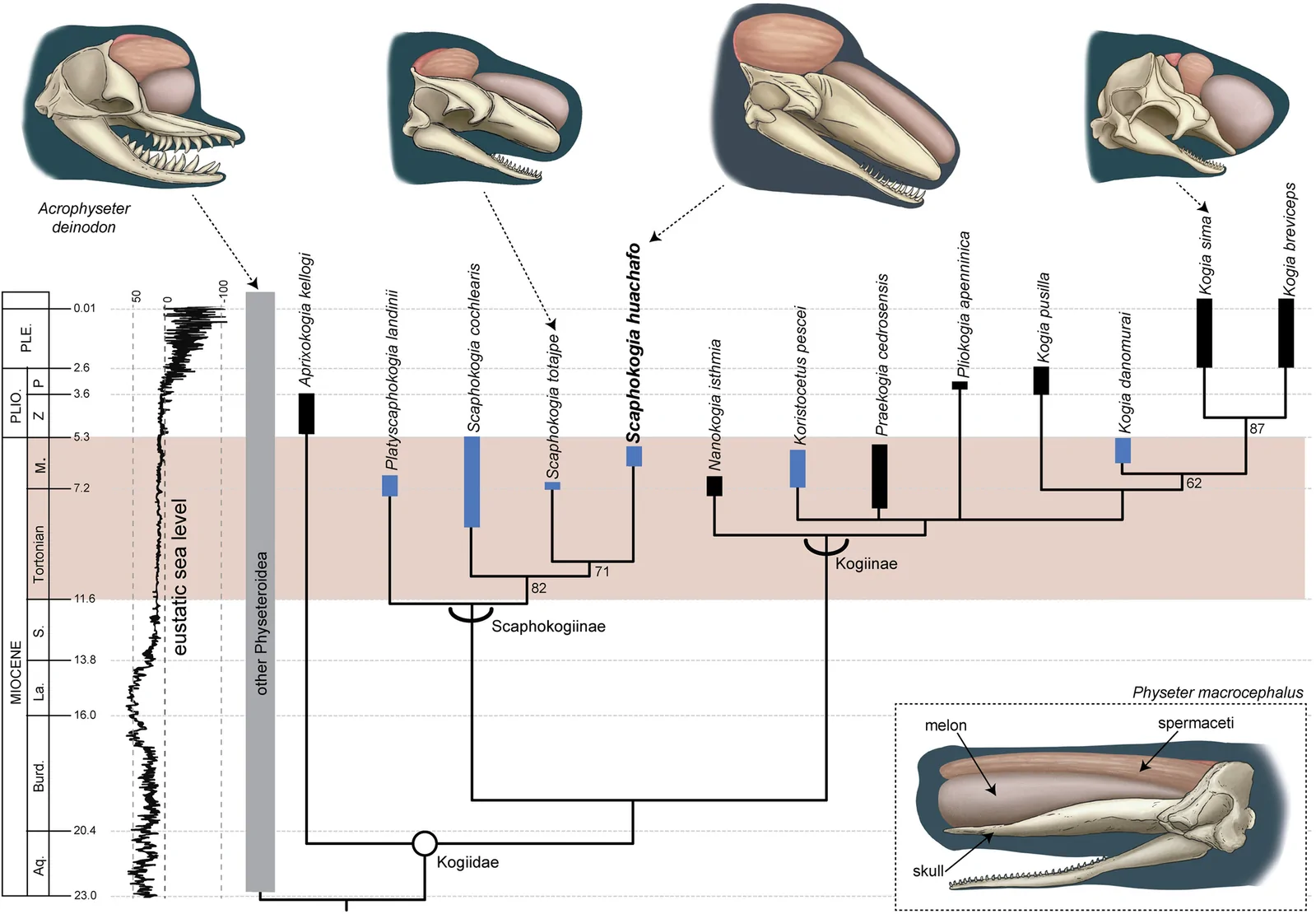
Phylogenetic relationships of *Scaphokogia huachafo* sp nov. within Kogiidae (taxa highlighted in blue originate from the Late Miocene of southern Peru). Time-calibrated phylogenetic tree obtained from 315 most parsimonious trees with a length of 257 steps, a CI of 0.397 and a RI of 0.694.

Our results recover *S. huachafo* as deeply nested within Kogiidae, specifically as a highly derived member of both the subfamily Scaphokogiinae and the genus *Scaphokogia* (Fig. 2; Supplementary Fig. 4). *Scaphokogia huachafo* shares several morphological traits with other members of the genus *Scaphokogia*, including e.g. a tubular rostrum, the inferred hypertrophied nasal complex, and the bowl-like supracranial basin. Our results are consistent with previous works showing Kogiidae as a monophyletic clade, comprising two subfamilies: Scaphokogiinae (*Scaphokogia* spp., *Platyscaphokogia*) and Kogiinae (*Nanokogia*, *Praekogia*, *Koristocetus*, *Pliokogia*, *Kogia*).

### Differential diagnosis

A species of *Scaphokogia* with an estimated body length of approximately 270 cm. Due to extensive pachyostotic bone development and partial ossification of the mesethmoid, the rostrum of *S. huachafo* is notably more robust, larger, and more massive than those of *S. totajpe* and *S. cochlearis*. Eight large and well-defined maxillary alveoli are present ventrally; in contrast, distinct dental alveoli are mostly replaced by an alveolar groove in adult individuals of other species of the genus. The size of the alveoli in *S. huachafo* suggests that teeth may have been present at an earlier ontogenetic stage and subsequently lost or reabsorbed since the alveoli are filled in with spongy bone. The facial region exhibits a remarkable asymmetry, characterised by a pronounced leftward displacement of both external bony nares beyond the cranial midline, with the left naris being considerably larger than the right (Supplementary Notes 2). The right antorbital notch is much robust than the laminar-like left antorbital process, which extends dorsally toward the rostrum and posteriorly to the postorbital process, parallel to the anterior wall of the supracranial basin. In dorsal view, the left antorbital region extends further anteriorly than the right, emphasising pronounced cranial asymmetry. Taphonomic distortion is ruled out by the absence of deformation elsewhere on the skull. The anterior wall of the supracranial basin is more robust than in other species of the genus due to pachyostotic growth of the right premaxilla. Posteriorly, the supracranial basin is proportionally smaller than in *S. totajpe* and *S. cochlearis*, but dorsoventrally deeper than in the latter species. It is mostly circular in outline and restricted to the facial region. The asymmetric deepening of the basin extends into the intracranial cavity, which is more dorsoventrally compressed on the right side, a condition comparable to that observed in extant *Kogia*. The temporal fossa is anteroposteriorly elongated, resembling *S. totajpe* and differing from the enlarged fossa of *S. cochlearis*. Finally, the nuchal crest strongly overhangs the occipital condyles, which are proportionally smaller than in other species of *Scaphokogia*.

## Discussion

Among mammals, directional cranial asymmetry is a distinctive feature of most odontocetes, linked to sound production, echolocation, and the structures involved in these processes^26^. In early diverging crown clades, such as physeteroids, ziphiids and platanistoids, the asymmetry is pronounced, as evidenced by the distribution of their bones and enlargement of nasal organs^10^. Asymmetry in head tissues and associated structures in physeteroids (e.g., the spermaceti, melon, and surrounding forehead organs) is also evident from early foetal stages^11,12,26^, supporting the idea of a derived developmental pattern distinct from other toothed cetaceans (Synrhina).

**Fig. 3:**
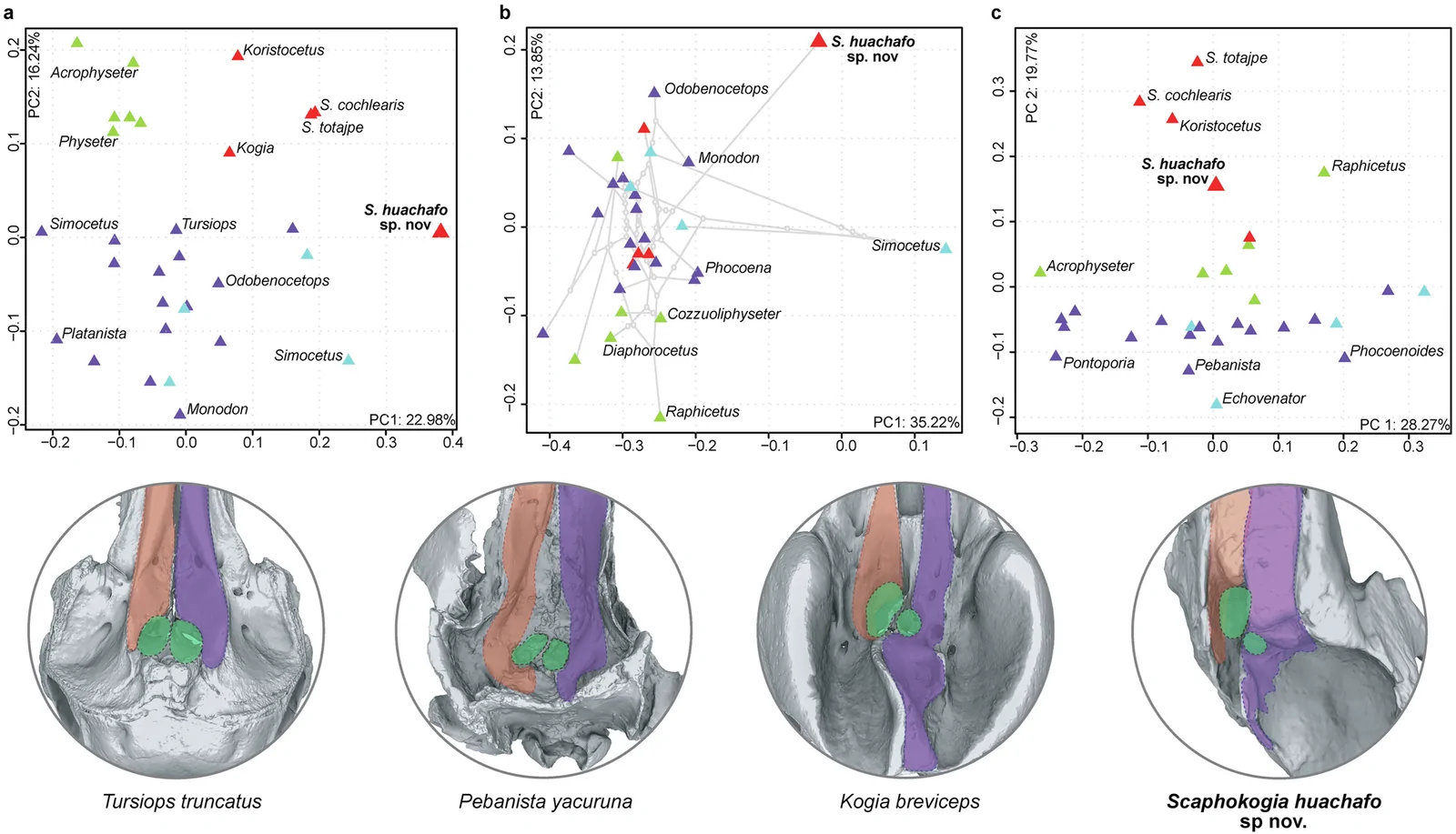
Arrangement of craniofacial bones and asymmetry in Odontoceti. Morphospace (**a**), phylogenetic PCA (**b**), and asymmetry analyses (**c**) of odontocetes, with particular focus on Kogiidae. Skulls shown at the bottom illustrate differences between the left (orange) and right (purple) premaxillae and variation in the size of the external bony nares.

Several morphometric analyses have previously quantified cranial asymmetry in cetaceans^10,26–29^, including sperm whales; however, the unique characteristics and evolutionary novelties of their skulls have not been fully considered. This is particularly relevant because some structures (e.g., the nasal bones) are lost in sperm whales^19,22,30^, whereas others (e.g., the supracranial basin) are unique to this clade. For this reason, our morphometric analyses of craniofacial bone arrangement focused on Physeteroidea and early odontocetes (Supplementary Fig. 5). In our initial morphometric analysis, physeteroids occupied a distinct region of the morphospace relative to synrhines or stem odontocetes (Fig. 3a; Supplementary Fig. 6). Beyond overall differences in the development and relative size of the left and right premaxillary bones, this distinct clustering of physeteroids is primarily driven by the curvature and excavation of the premaxillae associated with the development of the supracranial basin to house the enlarged nasal organs^13,14^. A similar pattern is observed in Kogiidae (Supplementary Notes 3), in which cranial asymmetry is driven not by premaxillary curvature, but rather by differences in size and extent between the left and right premaxillae. In this group, the left premaxilla extends only a short distance posterior to the bony nares, whereas the right premaxilla is greatly enlarged and extends far posteriorly. Within Kogiidae, some separation can be observed between Kogiinae and Scaphokogiinae. These clades are easily distinguishable based on the depth and extent of their supracranial basin and the presence of two or three cavities within this basin^19,31^, created by the sagittal facial crest. Within the supracranial basin of *Kogia*, the premaxillary fossa and the right maxillary fossa house most of the organs of the nasal complex^32^, which have higher capabilities for propagating and reflecting sound waves^33,34^, restricting the left maxillary fossa’s role as a supplementary anchor site for the enlarged facial muscles^35^, the latter being mainly responsible for sound modulation. It is not possible to assess the in vivo extent of the nasal organs in *Scaphokogia*, as the preservation of soft tissues in deep time is extremely rare. However, the attachment sites of these structures clearly indicate that the sound production system was well-developed and distinct from that of *Kogia*. Within Kogiidae, *S. huachafo* occupies the most extreme position in the morphospace. Given the strong statistical support evidenced by the MANOVA results (*p* = 0.001) and the observed *K* value (0.6072), both indicative of a robust phylogenetic signal, a phylogenetic principal component analysis (phyloPCA) was subsequently performed to assess phylogenetic structuring and investigate potential instances of adaptive radiation among the examined clades.

Within the phyloPCA (Fig. 3b), most odontocetes cluster in a relatively central position, with only a few taxa dispersing toward the extremes of morphospace. Among these the walrus-like dolphin *Odobenocetops* and the stem sperm whale *Rhaphicetus*. Somewhat unexpectedly, the stem odontocete *Simocetus* is also recovered as an outlier in this analysis. However, its position is likely an artefact of the absence of other stem taxa, such as archaeocetes, from the analysis. Furthermore, it is noteworthy that some platanistids, including *Pebanista*, are recovered in close proximity to certain physeteroids. This pattern may reflect convergent evolution in the shape of the circumnarial basin of platanistids and the supracranial basin of physeteroids. Once again, *S. huachafo* is recovered in an extreme position within the phyloPCA, even when phylogenetic signal is explicitly taken into account, indicating its exceptional occupation of morphospace and a high degree of shape innovation. These ordination patterns are further supported by the residual randomisation ANOVA, which despite marginal statistical significance (*p* = 0.076) indicates that group membership explains a substantial proportion of shape variation (*R*² = 0.224), consistent with biologically meaningful differentiation among clades. Finally, an additional analysis focusing exclusively on asymmetry between the left and right premaxillae was conducted. In this analysis, the genus *Scaphokogia* was unequivocally recovered as the most asymmetric group of cetaceans in the sample, clustering in an extreme position along PC1 and PC2. Surprisingly, *S. huachafo* was not the most extreme taxon; instead, this position was occupied by *S. totajpe*, the smallest species within the genus. This result likely reflects the analytical focus on direct left–right differences in premaxillary morphology rather than on overall bilateral symmetry. Nevertheless, the analysis clearly and consistently identifies *Scaphokogia* as the most asymmetric cetacean genus in the dataset.

One of the most distinctive features of *Scaphokogia* is its massive tubular rostrum. Additionally, all species within this genus exhibit some degree of ventral deviation of the snout (klinorhynchy)^36^, with the angle between the rostrum and the main cranial axis varying from ~16–21° in *S. cochlearis* to 27° in *S. totajpe* and 31° in *S. huachafo* (Supplementary Fig. 9). The rostrum of *S. huachafo* is further characterised by moderate longirostry, bulging due to pachyostotic bone growth, and partial ossification of the mesorostral cavity. These features likely shifted the centre of gravity of the animal forward. Bone mass increase in the skull and/or skeleton in marine mammals is usually associated with active benthic feeding (e.g. sirenians, walruses, some archaeocetes) by providing greater hydrostatic control^37–39^ when compared to facultative benthic feeders as extant *Tursiops*^40^.

**Fig. 4:**
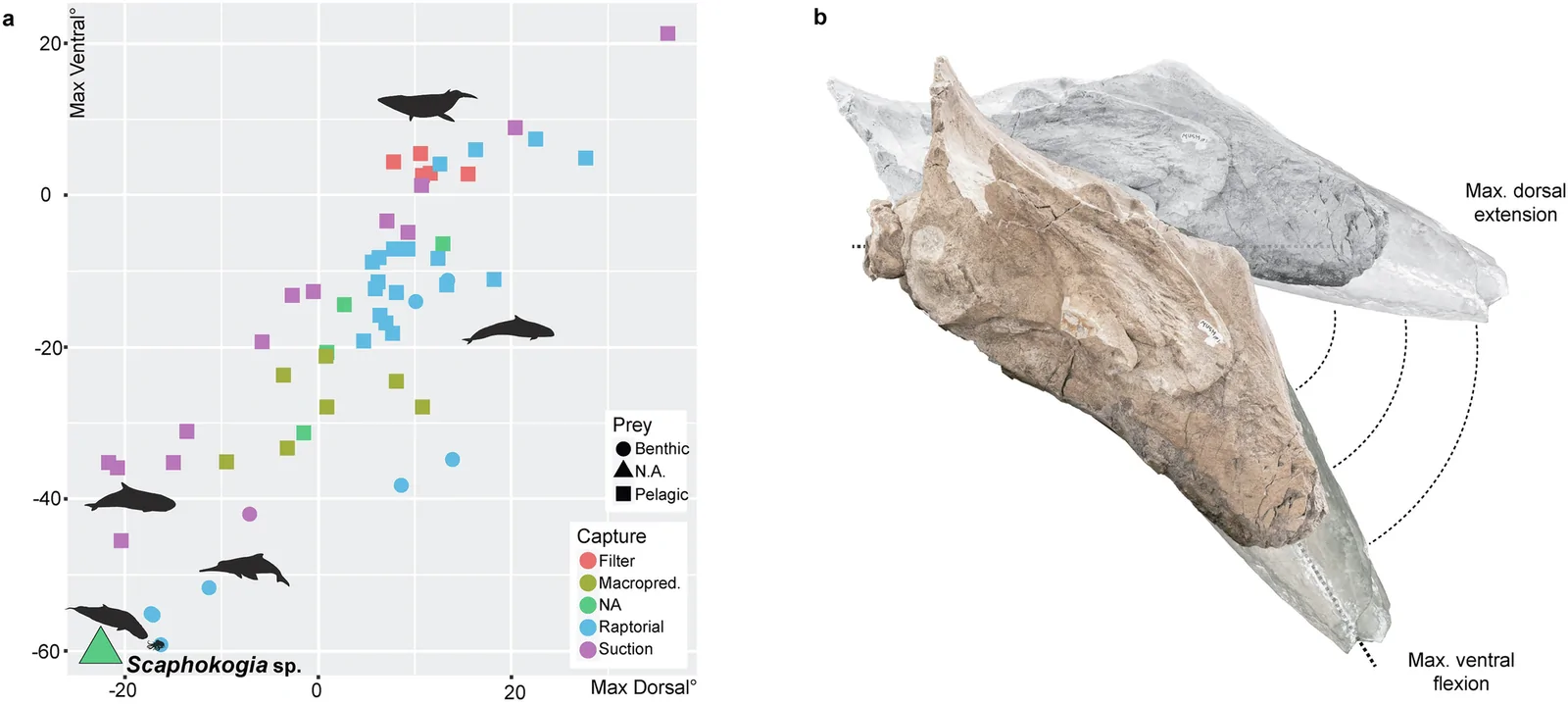
Benthic adaptations in *Scaphokogia* spp: Range of Motion (ROM) of the atlantooccipital joint in Cetacea, including *Scaphokogia* sp. MUSM 171 (**a**); shapes indicate the general morphology of the clusters recovered. Hypothesised reconstruction of the maximum dorsal extension and ventral flexion of the atlanto-occipital joint in *Scaphokogia huachafo* (**b**) based on *Scaphokogia* sp.

An additional specimen identified as *Scaphokogia* sp. (MUSM 171) preserves the atlas, allowing for the reconstruction of the range of motion (ROM) of the atlantooccipital joint^41^. When the neck was fully flexed dorsally, the rostrum formed a ventral angle of 22° with the main body axis, and at maximum ventral flexion, the angle increased to 55° (Fig. 4). This suggests that the rostrum was permanently oriented ventrally. The skull asymmetry of *Scaphokogia* spp. is also evident in the occipital condyles: the right condyle is broader than the left one and is more dorsolaterally oriented, while the left one is larger but slightly more ventrally positioned (Supplementary Fig. 7). The corresponding articular facets of the atlas are likewise asymmetrical, with the right facet projecting slightly ventrally. This asymmetry is further accentuated by anteroposterior thickening of the right half of the bone, which gives it a trapezoidal profile. As a consequence, movements at the atlanto-occipital joint most likely followed a curved rather than linear trajectory.

Collectively, these features indicate that the skull of *Scaphokogia* exhibited a ventrolateral torsion relative to the main body axis, suggesting that the cranio-cervical articulation did not operate strictly within a single vertical plane. The combination of pronounced skull asymmetry and significant klinorhynchy points to a highly specialised benthic foraging ecology for *S. huachafo* and other members of the genus. Klinorhynchy is generally regarded as an energy-efficient adaptation for benthic feeding, as seen for example in sirenians^42^. Together, these morphological traits support a benthic forager hypothesis, in which the pachyostotic ventral surface of the skull (Supplementary Fig. 8) may have aided hydrostatic control during benthic foraging.

Benthic feeding ecologies have been previously proposed for some ancient cetaceans like protocetids, as well as other coeval cetaceans from the Pisco Formation, particularly the walrus-dolphin *Odobenocetops*, which also exhibits a ventrally oriented skull and rostrum^43,44^. Most bottom-feeding marine mammals rely on vision and specialised orofacial structures with varying degrees of motor and sensory functions for detecting and acquiring food, as seen in *Odobenocetops*. Since *Scaphokogia* appears to lack specialised orofacial structures, we propose that it instead evolved unique echolocation adaptations to facilitate prey detection and acquisition in soft substrates. Finally, the co-occurrence of multiple genera of specialised benthic feeders in Late Miocene ecosystems reflects the higher abundance of benthic invertebrate taxa and the relatively stable environmental conditions of the region. This stability, especially in shallow marine and coastal habitats, lasted until the Pliocene^27,28^, when the final marine turnover restructured the oceans into their modern configuration.

*Scaphokogia huachafo* represents a new species of pygmy sperm whale from the Late Miocene Pisco Formation in Peru, one of the most bizarre fossil cetaceans known so far due to its highly idiosyncratic cranial architecture. *S. huachafo* is recognised as the most derived species of the genus *Scaphokogia* in our subsequent phylogenetic analyses, displaying the typical cranial characters of the genus but in its most derived state. Furthermore, 3D morphometric analyses of sperm whales and other odontocetes indicate that this new species exhibits a unique craniofacial asymmetry among cetaceans, due to the disproportionate positioning of the external bony nares and premaxillary bones. Additionally, reconstructions of the atlantooccipital joint in this genus, along with the ventrally oriented rostrum, indicate that this species was able to significantly flex its skull ventrally. This, combined with the massive tubular rostrum, suggests that this animal was a specialised benthic feeder, capable of sweeping through the bottom of the shallow coastal environments of the Late Miocene. *Scaphokogia huachafo* represents one of the most highly derived morphologies ever attained by sperm whales in the past, occupying an ecological niche with no modern parallel across odontocetes, shortly before the final turnover that gave rise to our modern marine mammal fauna.

## Methods

### Phylogenetic analyses

To assess the phylogenetic relationships of *Scaphokogia huachafo* within Kogiidae, a phylogenetic matrix was constructed using Mesquite 3.70^45^, comprising 68 morphological characters and 38 operational taxonomic units. This matrix was based on previous research on Physeteroidea systematics^16,19,22,31,43,44,46,47^, yet several characters were re-defined due to ambiguous coding (Supplementary Notes 4). The cladistic parsimony analyses were performed in PAUP 4.0a169^48^ taking characters as unordered and ordered. Because of the high number of poorly supported nodes (less than 50% bootstrap support) and polytomies resulting from the first parsimony analysis, additional analyses were performed by down-weighting of homoplastic characters with *k* values of 2, 3, 10, 20, and 40. Because the topology remained unmodified, the lowest value was kept (*K* = 3). The statistical support for the results was calculated on the basis of 1000 bootstrap replicates. To further test the consistency of our results, the same analyses were also performed in TNT 1.6^49^ using the Traditional Search and New Technologies algorithms.

### Arrangement of craniofacial bones and asymmetry

To characterise the patterning and spatial arrangement of the principal facial bony structures in cetacean skulls, a series of geometric morphometric analyses was conducted. Because most previous morphometric analyses of cetacean skulls have focused on Synrhina^10,29,30,32^, existing landmarking protocols are not directly reproducible across the full range of cetacean cranial morphologies. We therefore developed a new landmarking protocol, optimised for the facial region of sperm whales, that explicitly accounts for their distinctive cranial architecture and asymmetry. Landmarks were digitised using MorphoDig^50^ and comprised a total of 37 points (Supplementary Data 1). These included seven type I landmarks: the preorbital process of the frontal (left and right), the dorsal margin of the antorbital notch (left and right), the floor of the antorbital notch (left and right), and the midpoint of the nasal septum. In addition, two sets of semi-landmarks, each consisting of 15 calibration points, were used to capture the shape of the premaxillary bones (left and right) in the facial region, extending anteriorly from the level of the antorbital notch (Supplementary Fig. 5). The rostrum was not landmarked due to its high plasticity, as this structure responds to modular evolution, primarily related to the specific feeding ecology of each species^51^. Additionally, no landmarks were taken on the nasal bones as these are completely missing in kogiids. Furthermore, since at least two scaphokogiines (*Scaphokogia huachafo* MUSM 972 and *Scaphokogia totajpe* MUSM 973) have not preserved the entirety of the supracranial basin, no landmarks were taken on the maxillae. The dataset consisted of 31 taxa, including 11 sperm whales, 4 stem odontocetes and 16 Synrhina (Supplementary Table 2). The craniofacial asymmetry (CFA) analyses were performed in R 4.3.2^52^ using the package geomorph^53^, calculating the phylomorphospace and PhyloPCA. Statistical support for the morphometric analyses was assessed using MANOVA tests applied to both the non-phylogenetic morphospace and the phylogenetic PCA, allowing evaluation of the presence or absence of phylogenetic signal. In addition, a symmetry analysis was performed to quantify asymmetry between the left and right premaxillae.

### Benthic adaptations

To measure the range of dorsoventral motion (ROM) allowed by the atlantooccipital joint (the articulation between the occipital condyles of the skull and the atlas bone), the skull and atlas are positioned in their most ventrally flexed and dorsally extended positions. The angle formed by the rostrum and the main body axis is then measured. The ROM was measured in *Scaphokogia* sp. MUSM 171, the only specimen of the genus with a preserved atlas (Supplementary Fig. 7). Unlike other odontocetes, where the rostral orientation is measured using the plane formed between the distal tip of the premaxillae and the dorsal margin of the orbit, in *Scaphokogia* sp. MUSM 171, the rostral plane is averaged using the angles from the roof and floor of the rostrum. These measurements were incorporated into the dataset of Okamura and Fujiwara^41^, expanding the dataset to include 31 species of cetaceans (Supplementary Data 2), including the extant sperm whales *K*. *breviceps* and *Physeter macrocephalus*.

### Bone development patterning

The holotype specimen of *Scaphokogia totajpe* MUSM 973 was CT-scanned at Materials, Research & Technology facilities (Lima, Peru). The data was first handled in Dragonfly 3D (Version 2024.1 for Windows) to orientate the skulls. Three slices were extracted and segmented: one from near the distal tip of the rostrum (Supplementary Fig. 8), another from the midlength of the rostrum, and the final one at the level of the external bony nares. Binarized tiffs were exported for performing local thickness analyses. Thickness maps and measurements of the slices were completed using the Local Thickness tool in Fiji (v.1.54 f)^54^.

### Reporting summary

Further information on research design is available in the Nature Portfolio Reporting Summary linked to this article.

## Supplementary information

Transparent Peer Review file

Supplementary Information

Description of Additional Supplementary Files

Supplementary Data 1

Supplementary Data 2

Reporting Summary

## Data Availability

Code used in this paper is available at: https://doi.org/10.5281/zenodo.22095873.

## Data availability

The associated 3D Surface scans of *Scaphokogia huachafo*^55^ are available at Morphomuseum: https://doi.org/10.18563/journal.m3.304.
