# Supplementary material for: Extreme cranial asymmetry in extinct benthic feeding sperm whales
Source: Commun Biol. 2026 Nov 9;9:1406. doi: 10.1038/s42003-026-11074-2 (PMC13748650; doi:10.1038/s42003-026-11074-2)
Supplement: Supplementary file 1 [file 42003_2026_11074_MOESM1_ESM.pdf]

nature portfolio

Peer Review File

Extreme Cranial Asymmetry in Extinct Benthic Feeding Sperm Whales

Corresponding Author: Dr Aldo Benites-Palomino

This file contains all reviewer reports in order by version, followed by all author rebuttals in order by version.

Attachments originally included by the reviewers as part of their assessment can be found at the end of this file.

Version 0:

Reviewer comments:

Reviewer #1

(Remarks to the Author)
The authors describe an interesting new extinct physeteroid species, Scaphokogia huachafo, from the Miocene of Peru. They characterize in detail the sometimes bizarre anatomical aspects of this species, place the new taxon into a phylogenetic framework, and use morphometrics to document the extreme morphology/asymmetry of the skull from this species. Overall, the paper is generally clearly written and a useful contribution to the field. The authors might consider the following comments in revising/improving their manuscript.
1) Line 27. "Fossils offer critical insights into morphological changes beyond those observed in extant species and, in rare instances, unveil evolutionary novelties." A minor point, but isn't it a fact that fossils commonly unveil evolutionary novelties? There are large, completely extinct clades of species (e.g., sauropods) that greatly extend the morphological diversity of what is known from just extant species, so I think this opening sentence should be restated to make more sense.
2) Line 37. "Morphological reconstruction of the atlanto-occipital joint suggests enhanced ventral neck flexibility, optimized for benthic foraging behaviours." It should be noted in the abstract that the neck bones are from another species in the genus. As the abstract reads currently, it sort of implies that these neck bones are from the new species, and really, the neck bones could be completely different from this other species of Scaphokogia (or not). Throughout the text, this leap of inference from one species to another in clade is somewhat dubious, so it should always be clearly expressed what is assumed by inferring that the morphology of a congener is identical, or nearly so, in another species in the same extinct genus.
3) Line 39. "…greatly…" I think the new species does expand the range of skull architecture in Cetacea, but I am not so sure about 'greatly' according to the morphometric analyses done. The species closely related to the new one show similar morphologies, and the new one is different, but maybe not that much different and based on a very limited sampling of individuals in different species. Extended data Fig. 9 sort of shows similar morphologies among the various species in this genus that were already described?
4) Line 40. "S. huachafo occupied now-extinct ecological niches, feeding near the bottom of the shallow coasts of Peru during the late Miocene, a crucial moment for oceans evolution." I guess I would argue that the ecological niche of the new species is certainly 'now-extinct' as a fact, but its exact ecological niche is more guesswork than anything, and I am not sure if any of the authors would bet the life of their firstborn on their best hypothesis for this species' niche being accurately reconstructed? Throughout the text when the authors infer ancient behaviors or ecological roles based on fragmentary fossils (no lower jaws, no neck bones, no postcranials at all), I think it would be better to make language clear that all of these reconstructions are quite speculate and use words like 'may be', 'could be', 'was possibly', etc. to document that it is quite challenging to infer specific behaviors from fragmentary fossils. It is not that I think the authors' inferences are dubious in any way, but these reconstructions will always be pretty speculative, even if much more complete fossil remains are eventually recovered. And, in the present case, bones from closely related species are sometimes used to reconstruct what the new species looked like or how it behave, and this makes things even more speculative. The assumptions made when applying knowledge of the anatomy from one species to another should be explicitly stated at least once in the text.
5) Line 122. Phylogeny: general stuff. It should be made more clear in the figure legends of the tree figures what is being shown. Especially for the online tree figure, there are multiple trees shown, and it is not clear from the figure legend what the various trees represent (unless I missed something in reading the text?).
6) Line 122. "Our results are consistent with previous works showing a high support of Kogiidae as a clade an in its

subdivision into subfamilies with their respective composition: Scaphokogiinae (Scaphokogia spp., Platyscaphokogia) and Kogiinae (Nanokogia, Praekogia, Koristocetus, Pliokogia, Kogia).” Really, no clades in these trees show ‘high support’ for any relationships, so this statement should be revised? “Kogiidae as a clade an in its subdivision” should be edited to “Kogiidae as a clade and in its subdivision”?
7) Line 140. “In adults of other members of the genus, alveoli are partially reabsorbed and have mostly filled with spongy bone, suggesting that functional teeth are greatly reduced.” An example of the incompleteness of the feeding apparatus of this species is that the authors are not really sure whether this new species has no teeth, many teeth, vestigial teeth, or fully formed teeth, and if any teeth are present, their morphology is unknown. So, I think the authors should be aware that some (many?) readers will be skeptical that they can confidently reconstruct how this new species was feeding back in the Miocene, when no teeth, no lower jaw, no neckbones, etc. are preserved in this admittedly interesting/intriguing specimen. So, as noted above, I think it would benefit the authors to almost never be definitive in the language they choose when describing the functional morphology/behavior/ecology of this extinct species and instead use language that reflects the challenges of reconstructing ancient ecology/behavior in fragmentary fossils.
8) Lines 204-227. This section includes the authors’ interpretation of functional morphology of the new species and this interesting genus, and it is not that I do not agree with their interpretations necessarily, but they should be explicit in this section about what assumptions they make in reconstructing ancient behaviors given that the same bones are not always preserved in different species in this genus yet they cobble together a story based on bones from different members of this genus. This sort of extrapolation may be justified, but surely has its limits in terms of how far analogies/homologies among species should be extended down a phylogenetic tree.
9) Line 254. “occupying an ecological niche with no modern parallel across odontocetes”. As an example of simple ways to temper language so as to get across uncertainty, could say here something like “likely occupying an ecological niche with no modern parallel across odontocetes”.
10) Line 266. “Because of the high number of poorly supported nodes (less than 50% bootstrap support) and polytomies resulting from the first parsimony analysis, additional analyses were performed by down-weighting of homoplastic characters with k values of 2, 3, 10, 20, and 40. Because the topology remained unmodified, the lowest value was kept (K = 3).” It is difficult to understand what is being said here, and the text is pretty ambiguous as currently written. The authors say the ‘topology remained unmodified’, but does this mean that the equally weighted parsimony analysis (the ‘first parsimony analysis’?) yielded a strict consensus tree of most parsimonious trees that did not change no matter what value of K was used to weight characters differentially? When they say that ‘Because the topology remained unmodified, the lowest value was kept (K = 3)’, this does not make sense to me, because they also seemed to do a ‘lower’ K analysis in which K=2, so this is confusing to me. Furthermore, as mentioned above, it is not clear to me what the online Figure 4 shows. There are four trees shown that all differ a bit, but all have low support values. It is not clear from the text which trees these are. I also recommend that an equally weighted parsimony analysis result be shown and that Bremer supports should be given at nodes as an alternative to the bootstraps shown, which will help the reader to get a feel for the level of support, which generally seems quite low. Lots of unevenly distributed missing data can yield very low bootstrap and Bremer supports, so it would be nice to show both and the equally weighted parsimony tree.
11) Line 273. Change “Since most of previous studies” to “Since most previous studies”.
12) Line 276. Change “taking in account their morphological peculiarities” to “taking into account their morphological peculiarities”.
13) Line 280. “The rostrum was not landmarked due to its high plasticity, as this structure responds to modular evolution, primarily related to the species' ecology.” This sentence could use a rewrite. Does ‘high plasticity’ refer to environmentally driven, within species variation that is extensive or to something else? Since there seems to be only one specimen for the new species, is the inference of high plasticity based on data from other species? Either way, a citation should be given here for this assertion. I also was not sure what was meant by ‘this structure responds to modular evolution’? Again, how is this known, which studies have shown this, and why would this fact lead to not measuring the rostrum?
14) Online Figure 10. Most of the skeletal anatomy for the new species is not known. How much postcranial anatomy is known from other species in the genus and also morphology of the lower jaw. It might be good to add which species were used to make this artist’s reconstruction, as the new species is based on just a fragmentary specimen.

Reviewer #2

(Remarks to the Author)
This paper is a well-written, worthwhile addition to the literature on a bizarre group of cetaceans, the kogiids. It is a further example of the the incredible fossils and the excellent work being done on them in Peru. The paper describes a new species of kogiid from the Scaphokogiinae subfamily, and it appears to have a very distinct set of morphological features which hint at a specialised feeding ecology. There are two main claims of the paper. First, is the extreme asymmetry of species. And second, the high degree of ventral flexibility. My main issue with the paper is the justification used for the former main claim. The authors have used PCA to quantify asymmetry, but this is not what PCA does. Rather, it is quantifying the shape variation in the craniofacial region. This may well be due to asymmetry, but it could just be differently shaped. Similarly, saying S. huachafo is more asymmetrical than all other taxa is not quite right, it is simply in a distinct region of morphospace. I would recommend considering at least one method of directly quantifying asymmetry to be employed. At a minimum, the differences in the morphology described by the landmark configurations should be described and compared to highlight if it is indeed due to increased asymmetry. Some of this detail is already in the extended description. Figure 3 is a good start in visualising the differences but I believe the use of the R package landVr to show landmark variation would be very useful indeed. Lastly, a very speculative question from me is whether the high diversity of this genus seen in this region could be potentially due to some taxa actually representing an ontogenetic series or sexual dimorphism?

Overall, this is a very solid paper, and once the recommendations below and in the attached PDF are made, I look forward to

seeing it in the literature. Well done to all the authors.

Extended data notes

ESM notes

ED Fig 1: should be a comma after '(b)'
ED Fig 3: should be a comma after '(b)'
ED Fig 6: the order of the figure caption is incorrect, I'm assuming A and C are the regular PCA and B and D are the phylogenetic PCA (which is actually a phylogenetically aligned PCA if you have used Geomorph to do it). Please correct the figure captions accordingly and label the PC axes of your pPCA/PaPCA so that they are different to the standard PCA (i.e. PC1 vs pPC1).

line 84: should be 'such a condition'
line 84: should be 'sunken'
line 107: 'nares' should be 'naris'
lines 122-126: could this exotosis potentially indicate a functional dentition? I know you do mention the spongy internal bone of the teeth in the main paper.
line 125: do you mean 'unintentional' here?
line 166: should be 'much more vertical'
line 174: should be 'much more ventral'
line 203: 'is' should be 'are'
line 219: delete the word the word 'the' from the very end of the line
line 221: 'choana' should be 'choanae'
line 226: 'littel define' should be 'poorly defined'
line 242: delete 'impeding to recognise most of the structure; nevertheless'
line 252: should be 'interpreted as the dorsal'

Dr Travis Park

Reviewer #3

(Remarks to the Author)
Dear colleagues,

The manuscript by Aldo Benites-Palomino et al. presents the interesting and important discovery of an unusual extinct toothed whale showing exceptionally developed cranial asymmetry combined with other unusual evolutionary adaptations. I believe this is a spectacular specimen which improves our knowledge on extreme manifestations of animal evolution, and it will be of broad public interest and attention and a source of inspiration for applied science. Therefore, it is worth to be published in Communications Biology.

The suggestions which should be considered before the publication:

1. The Results section in the main text is reduced by the diagnosis with the remarks which are the small part of the description, while the description itself is in the supplementary information. This should be reorganized: the diagnosis should be clearly separated following the ICZN requirements and have a clear differential formulation, and the descriptive elements (starting from the size of the specimen) should follow it and include the references to the supplementary files. Also, the description in the main text should be expanded to give an overall idea of the specimen. Given that its current version contains only 200 lines, most of it can be placed in the main text with some editing.

2. Quantification of directional asymmetry should be done in the ways comparable with previous studies, e.g. the partial Procrustes distance of the asymmetric component of shape to the corresponding symmetric component (Laeta et al. 2023), the sum of radii (Coombs et al. 2020) and lateral displacement of landmarks (Huggenberger et al. 2017). See also Ness (1967) and Hirose et al. (2015). At least, several criteria should be considered. Specifically, it should be clearly shown that the directional asymmetry in the new species exceeds all the previously known records by all the measured criteria.

3. Clinorhynchy (l. 221-223) and hyperostosis of the rostrum (l. 225-227) should be extensively discussed for their possible functions. Relation of clinorhynchy to benthic feeding in toothed whales has been discussed in several works from Monteiro-Filho et al. 2002 to Galatius et al. 2020 (see some refs below). Equally, rostral ossification should be compared at least with that of beaked whales with its possible evolutionary implications (e.g., Cozzi et al. 2010; Lambert et al. 2011). In overall, here the great bulk of previous comparative anatomical research should be discussed.

4. Unique sensory adaptations (l. 233-235): This is a productive idea, potentially the core discovery of the research, that

should be expanded and clearly communicated as working hypotheses. What kind of unique echolocation adaptations or, if other than echolocation, what kind of sensory developments? How the heavy rostrum could contribute to these adaptations? How the deep supracranial basin could be involved? (Cranford et al. 2008, etc). Additionally, the overall idea of prey and other object search under the substrate should be better discussed (e.g., Rossbach and Herzing 1997; Mann et al. 2008), maybe with other suggestions if interesting (Gol'din 2014).

5. Methods and presentation. Full record of all the statistical data should be included as the supplementary data. TNT: Traditional Search and New Technologies algorithms: all the details should be added. All the resulting clade values and indices should be added to the figure depicting the phylogeny. PCA: PACA should be added to the PhyloPCA, and all the statistics should be provided. In addition, the interpretation of the PCA results (ED Fig 6) does not correspond to the authors' main idea: the species showing the same trends as the new one are Simocetus, Phocoena and Ankylorhiza, all showing little to no asymmetry (especially, Simocetus). There should be another explanation for the data.

Sincerely,
Pavel Gol'din

References

Cranford, T. W., Krysl, P., & Hildebrand, J. A. (2008). Acoustic pathways revealed: simulated sound transmission and reception in Cuvier's beaked whale (Ziphius cavirostris). Bioinspiration & biomimetics, 3(1), 016001.

Coombs, E. J., Clavel, J., Park, T., Churchill, M., & Goswami, A. (2020). Wonky whales: the evolution of cranial asymmetry in cetaceans. Bmc biology, 18(1), 86.

Cozzi, B., Panin, M., Butti, C., Podestà, M., & Zotti, A. (2010). Bone density distribution patterns in the rostrum of delphinids and beaked whales: Evidence of family-specific evolutive traits. Anatomical Record, 293(2), 235-242.

Galatius, A., Racicot, R., McGowen, M., & Olsen, M. T. (2020). Evolution and diversification of delphinid skull shapes. Iscience, 23(10).

Gol'din, P. (2014). ‘Antlers inside’: are the skull structures of beaked whales (Cetacea: Ziphiidae) used for echoic imaging and visual display?. Biological Journal of the Linnean Society, 113(2), 510-515.

Gol’din, P., & Vishnyakova, K. (2016). Habitat shapes skull profile of small cetaceans: evidence from geographical variation in Black Sea harbour porpoises (Phocoena phocoena relicta). Zoomorphology, 135(3), 387-393.

Hirose, A., Nakamura, G., & Kato, H. (2015). Some aspects on an asymmetry of nasal bones in toothed whales. Mammal study, 40(2), 101-108.

Huggenberger, S., Leidenberger, S., & Oelschläger, H. H. A. (2017). Asymmetry of the nasofacial skull in toothed whales (Odontoceti). Journal of Zoology, 302(1), 15-23.

Laeta, M., Oliveira, J. A., Siciliano, S., Lambert, O., Jensen, F. H., & Galatius, A. (2023). Cranial asymmetry in odontocetes: a facilitator of sonic exploration?. Zoology, 160, 126108.

Lambert, O., de Buffrénil, V., & de Muizon, C. (2011). Rostral densification in beaked whales: diverse processes for a similar pattern. Comptes Rendus Palevol, 10(5-6), 453-468.

Mann, Janet, et al. "Why do dolphins carry sponges?." PloS one 3.12 (2008): e3868.

Monteiro-Filho, E. L. D. A., Monteiro, L. R., & dos Reis, S. F. (2002). Skull shape and size divergence in dolphins of the genus Sotalia: a tridimensional morphometric analysis. Journal of Mammalogy, 83(1), 125-134.

Ness, A. R. (1967). A measure of asymmetry of the skulls of odontocete whales. Journal of Zoology, 153(2), 209-221.

Rossbach KA, Herzing DL. 1997. Underwater observations of benthic-feeding bottlenose dolphins (Tursiops truncatus) near Grand Bahama Island, Bahamas. Marine Mammal Science 13: 498–504.

Version 1:

Reviewer comments:

Reviewer #1

(Remarks to the Author)
The authors have improved their manuscript with their responses to the various reviewers. I will not comment on the specifics brought up by other reviewers (e.g., on taxonomic description and quantification of skull asymmetery) and leave this to editors and other reviewers to weigh in. For the minor points that I raised, the authors generally addressed all of my

concerns which mainly had to do with qualifying results just a bit. For the point on snout being a different module and being more 'plastic', where the authors note on line 294 that, "The rostrum was not landmarked due to its high plasticity, as this structure responds to modular evolution, primarily related to the specific feeding ecology of each species", the authors should add the reference that they provided in response to reviews here (Boessenecker et al.) and perhaps other published papers to back up this point, which is not obvious and should be referenced by prior work. I think maybe 'evolutionarily labile' might be a better term to use here rather than 'plastic' as plasticity in biology often refers to the ability of an organism to adjust phenotypically to different environmental conditions such that morphology depends on environment, while it seems the authors here are referring to the fact that the snout responds evolutionarily over phylogenetic scales and changes freely (among species) to fit ecological circumstances. At any rate, this work is very interesting and the authors should be commended for their efforts.

Reviewer #2

(Remarks to the Author)
The revised version of this manuscript does very well at addressing the reviewer comments. Other than the small suggested changes in the attached pdf, I believe that this article is now ready for publication. Well done to the authors.

Reviewer #3

(Remarks to the Author)
The revised manuscript has been well improved to the extent that now it can be published as is.

Open Access This Peer Review File is licensed under a Creative Commons Attribution 4.0 International License, which permits use, sharing, adaptation, distribution and reproduction in any medium or format, as long as you give appropriate credit to the original author(s) and the source, provide a link to the Creative Commons license, and indicate if changes were made.
In cases where reviewers are anonymous, credit should be given to 'Anonymous Referee' and the source.
The images or other third party material in this Peer Review File are included in the article's Creative Commons license, unless indicated otherwise in a credit line to the material. If material is not included in the article's Creative Commons license and your intended use is not permitted by statutory regulation or exceeds the permitted use, you will need to obtain permission directly from the copyright holder.
To view a copy of this license, visit https://creativecommons.org/licenses/by/4.0/

Wednesday, 18.02.2026

Editor

Communications Biology

Dear Dr.

Editor *Communications Biology*

Thank you for the opportunity to submit a revised version of our paper entitled: "EXTREME CRANIAL ASYMMETRY IN EXTINCT BENTHIC FEEDING SPERM WHALES" to *Communications Biology*. We extend our gratitude and are thankful for your editorial work and time invested in reading and reviewing our manuscript. We also wish to thank the referees for the extensive input regarding our work.

We have agreed with most reviewer comments unless explicitly stated otherwise. As several reviewers raised similar concerns, we begin by summarizing the major revisions made to the manuscript in the section below, before providing detailed, point-by-point responses to individual comments. This structure is intended to clarify how common issues were addressed across the manuscript while preserving specific responses where needed.

Additionally, we provide a consolidated list of reviewer-suggested modifications, indicating how each comment or concern was addressed and describing the corresponding changes implemented in the manuscript. Further revisions made independently of reviewer comments, but intended to improve clarity, organization, or interpretation, are also noted. A tracked-changes version of the manuscript is included as a separate file, documenting all revisions and facilitating comparison with the original submission.

We hope the revised document will better suit *Communications Biology* but are happy to consider further revisions. We reiterate our gratitude once again for the time invested.

Sincerely and on behalf of my coauthors,

Aldo Benites Palomino

General Comments on the Manuscript

1. Justification and interpretation of morphometric and asymmetry analyses

All reviewers raised concerns regarding the interpretation and potential ambiguity of our morphometric and asymmetry analyses. While we do not agree with all comments and recommendations (see specific replies below), we have nevertheless expanded the analytical framework and provided additional methodological details to directly address these points and to clarify the basis of our interpretations.

Within the geometric morphometric framework, we revised the phylogenetic tree to incorporate first and last occurrence data, yielding a time-calibrated tree for the OTUs. This modification allows the morphometric results to be evaluated explicitly in a temporal context and constrains interpretations of morphological change through time.

Results from the phylomorphospace and phylogenetic principal component analysis (phyloPCA) are provided in the code and supplementary material. A standard PCA was retained in the main text to maximize clarity and visual interpretability. Across all analyses, the genus *Scaphokogia*, and in particular *S. huachafo*, is consistently recovered as a distinct outlier, demonstrating that this result is not dependent on analytical framework.

To address concerns regarding cranial asymmetry, we conducted an explicit symmetry analysis using landmarked premaxillary surfaces as a reference (see main text for details). Although *S. huachafo* is not recovered as the most extremely asymmetric morphology, the analysis consistently identifies the genus *Scaphokogia*, and especially *S. totajpe*, as the most asymmetric taxon. This pattern is congruent with previous qualitative observations by our group (e.g. Benites-Palomino et al. 2020, 2021). A comprehensive reassessment of asymmetry in sperm whales and other Odontoceti lies beyond the scope of the present study, as both the morphometric and asymmetry analyses are intended to document and contextualize the distinctive morphology of the new species. Ongoing work by our group expands this approach to broader odontocete sampling and ontogenetic data.

2. Support for the new species within the context of the Pisco Formation

Within the Pisco Formation, multiple taxa exhibit gradual morphological transitions through time. For example, the aquatic sloth *Thalassocnus* comprises a succession of chronospecies traceable through the stratigraphic sequence. Although species are generally restricted to discrete stratigraphic intervals, at least one case documents the co-occurrence of two lineages within the same genus. This pattern provides an appropriate comparative framework for interpreting species-level differentiation within the Pisco Formation. In the case of the members of the genus *Scaphokogia*, but also *Platyscaphokogia* most of the occurrences also happen on specific intervals within the Pisco Formation on the East Pisco Basin and the Sacaco sub-basin. There is overlap observed between this new species and *S. cochlearis*, but the overall cranial morphology is evidently different between one and the other one to interpret these as the result of intraspecific variation.

Reviewer #1 (Remarks to the Author):

The authors describe an interesting new extinct physeteroid species, *Scaphokogia huachafo*, from the Miocene of Peru. They characterize in detail the sometimes-bizarre anatomical aspects of this species, place the new taxon into a phylogenetic framework, and use morphometrics to document the extreme morphology/asymmetry of the skull from this species. Overall, the paper is generally clearly written and a useful contribution to the field. The authors might consider the following comments in revising/improving their manuscript:

1) Line 27. "Fossils offer critical insights into morphological changes beyond those observed in extant species and, in rare instances, unveil evolutionary novelties." A minor point, but isn't it a fact that fossils commonly unveil evolutionary novelties? There are large, completely extinct clades of species (e.g., sauropods) that greatly extend the morphological diversity of what is known from just extant species, so I think this opening sentence should be restated to make more sense.

   Reply: The opening sentence has been modified.

2) Line 37. “Morphological reconstruction of the atlanto-occipital joint suggests enhanced ventral neck flexibility, optimized for benthic foraging behaviours.” It should be noted in the abstract that the neck bones are from another species in the genus. As the abstract reads currently, it sort of implies that these neck bones are from the new species, and really, the neck bones could be completely different from this other species of *Scaphokogia* (or not). Throughout the text, this leap of inference from one species to another in clade is somewhat dubious, so it should always be clearly expressed what is assumed by inferring that the morphology of a congener is identical, or nearly so, in another species in the same extinct genus.

Reply: The abstract has been revised to clearly state that the analysis was conducted on a different member of the genus. Nevertheless, it is important to emphasize that the majority of the inferences and interpretations derived from this analysis remain broadly applicable to most members of the genus. This is particularly relevant given the shared morphological traits across the genus, as well as the stratigraphic and paleoenvironmental characteristics observed at the localities where these specimens were recovered. As discussed in the General Comments on the Manuscript, specifically in the section addressing support for the new species within the context of the Pisco Formation, the comparative framework and geological setting provide a robust basis for extending these conclusions beyond the single taxon examined.

3) Line 39. “…greatly…” I think the new species does expand the range of skull architecture in Cetacea, but I am not so sure about ‘greatly’ according to the morphometric analyses done. The species closely related to the new one show similar morphologies, and the new one is different, but maybe not that much different and based on a very limited sampling of individuals in different species. Extended data Fig. 9 sort of shows similar morphologies among the various species in this genus that were already described?

Reply: The original description of the genus by de Muizon (1988) was based on a subadult and incomplete specimen (*Scaphokogia cochlearis*) and does not reflect current anatomical standards. A later study (Benites-Palomino et al., 2020) described a second species using the anatomical framework available at the time; several aspects of its terminology and interpretation have since been revised and are updated here. Previous treatments of this genus were therefore largely qualitative and lacked quantitative assessment of cranial variation. The present study provides the first quantitative evaluation of cranial morphology within *Scaphokogia*. Although the new species overlaps with congeners in morphospace, it consistently occupies an extreme position within that distribution. *Scaphokogia huachafo* does not represent a distinct cranial architecture, but rather documents the upper limits of variation expressed within this architectural framework in Cetacea.

4) Line 40. “S. huachafo occupied now-extinct ecological niches, feeding near the bottom of the shallow coasts of Peru during the late Miocene, a crucial moment for oceans evolution.” I guess I would argue that the ecological niche of the new species is certainly ‘now-extinct’ as a fact, but its exact ecological niche is more guesswork than anything, and I am not sure if any of the authors would bet the life of their firstborn on their best hypothesis for this species’ niche being accurately reconstructed? Throughout the text when the authors infer ancient behaviors or ecological roles based on fragmentary fossils (no lower jaws, no neck bones, no postcranials at all), I think it would be better to make language clear that all of these reconstructions are quite speculate and use words like ‘may be’, ‘could be’, ‘was possibly’, etc. to document that it is quite challenging to infer specific behaviors from fragmentary fossils. It is not that I think the authors’ inferences are dubious in any way, but these reconstructions will always be pretty speculative, even if much more complete fossil remains are eventually recovered. And, in the present case, bones from closely related species are sometimes used to reconstruct what the new species looked like or how it behave, and this makes things even more speculative. The assumptions made when applying knowledge of the anatomy from one species to another should be explicitly stated at least once in the text.

Reply: We agree with the reviewer that ecological and behavioural reconstructions based on fragmentary fossil material are inherently uncertain. The manuscript has been revised throughout to reflect this more explicitly, and speculative inferences are now consistently framed using cautious language (e.g., “may,” “could,” “possibly”). The statement in Line 40 has been modified accordingly to avoid implying a level of ecological resolution not directly supported by the available material. Comparative inferences are restricted to closely related congeners that are geographically, temporally, and phylogenetically proximate, and this assumption is now stated explicitly in the text. Under these conditions, the use of closely related taxa to infer missing anatomical information follows standard comparative practice, but its limitations are now

clearly acknowledged. In addition, the present study documents previously undescribed cervical and vertebral material and incorporates comparisons based on an additional specimen, which modestly expands the anatomical basis for these interpretations without eliminating their speculative nature.

5) Line 122. Phylogeny: general stuff. It should be made more clear in the figure legends of the tree figures what is being shown. Especially for the online tree figure, there are multiple trees shown, and it is not clear from the figure legend what the various trees represent (unless I missed something in reading the text?).

   Reply: Phylogeny references have been modified .

6) Line 122. "Our results are consistent with previous works showing a high support of Kogiidae as a clade an in its subdivision into subfamilies with their respective composition: Scaphokogiinae (Scaphokogia spp., Platyscaphokogia) and Kogiinae (Nanokogia, Praekogia, Koristocetus, Pliokogia, Kogia)." Really, no clades in these trees show 'high support' for any relationships, so this statement should be revised? "Kogiidae as a clade an in its subdivision" should be edited to "Kogiidae as a clade and in its subdivision"?

   Reply: We thank the reviewer for this comment. The wording has been revised to remove the phrase "high support" and to better reflect the level of branch support recovered in the analyses. Within the broader context of generally low support values across Physeteroidea, Kogiidae and its subdivision are consistently recovered, and the revised text now reflects this pattern without overstating statistical support.

7) Line 140. "In adults of other members of the genus, alveoli are partially reabsorbed and have mostly filled with spongy bone, suggesting that functional teeth are greatly reduced." An example of the incompleteness of the feeding apparatus of this species is that the authors are not really sure whether this new species has no teeth, many teeth, vestigial teeth, or fully formed teeth, and if any teeth are present, their morphology is unknown. So, I think the authors should be aware that some (many?) readers will be skeptical that they can confidently reconstruct how this new species was feeding back in the Miocene, when no teeth, no lower jaw, no neckbones, etc. are preserved in this admittedly interesting/intriguing specimen. So, as noted above, I think it would benefit the authors to almost never be definitive in the language they choose when describing the functional morphology/behavior/ecology of this extinct species and instead use language that reflects the challenges of reconstructing ancient ecology/behavior in fragmentary fossils.

   Reply: We agree with the reviewer that the feeding apparatus of *Scaphokogia huachafo* is incompletely preserved and that this necessarily limits the confidence of functional and ecological inferences. The text has been revised to avoid definitive language and to clarify explicitly which components of the feeding apparatus are preserved in each species and which are absent in the new taxon. Interpretations of feeding strategy are therefore presented as hypotheses. Inferences regarding feeding mode are further informed by comparisons with closely related sister taxa, acknowledging the assumptions and limitations inherent in this approach. We also clarify in the manuscript that benthic feeding in cetaceans commonly involves suction and that the potential presence, absence, or reduction of teeth does not preclude suction-based feeding strategies.

8) Lines 204-227. This section includes the authors' interpretation of functional morphology of the new species and this interesting genus, and it is not that I do not agree with their interpretations necessarily, but they should be explicit in this section about what assumptions they make in reconstructing ancient behaviors given that the same bones are not always preserved in different species in this genus yet they cobble together a story based on bones from different members of this genus. This sort of extrapolation may be justified, but surely has its limits in terms of how far analogies/homologies among species should be extended down a phylogenetic tree.

   Reply: We thank the reviewer for this comment. The assumptions underlying our functional interpretations have been clarified in the revised manuscript, as suggested here and in related comments. Although the atlas is referred to *Scaphokogia* indet., *Scaphokogia huachafo* exhibits the same asymmetry in the occipital condyles. This shared morphology provides the anatomical basis for the inference discussed in Lines 204–227.

9) Line 254. "occupying an ecological niche with no modern parallel across odontocetes". As an example of simple ways to temper language so as to get across uncertainty, could say here something like "likely

occupying an ecological niche with no modern parallel across odontocetes".

Reply: we have done the proper change

10) Line 273. Change "Since most of previous studies" to "Since most previous studies".

Reply: Change has been done

11) Line 276. Change "taking in account their morphological peculiarities" to "taking into account their morphological peculiarities".

Reply: Change has been done

12) Line 280. "The rostrum was not landmarked due to its high plasticity, as this structure responds to modular evolution, primarily related to the species' ecology." This sentence could use a rewrite. Does 'high plasticity' refer to environmentally driven, within species variation that is extensive or to something else? Since there seems to be only one specimen for the new species, is the inference of high plasticity based on data from other species? Either way, a citation should be given here for this assertion. I also was not sure what was meant by 'this structure responds to modular evolution'? Again, how is this known, which studies have shown this, and why would this fact lead to not measuring the rostrum?

Reply: The objective of the paper is to analyse facial region so rostrum information was not used. Analyses by colleagues have pointed out that rostrum evolution is completely independent from the rest of the skull, responding to a modular type of evolution.

Boessenecker, R. W., Fraser, D., Churchill, M., & Geisler, J. H. (2017). A toothless dwarf dolphin (Odontoceti: Xenorophidae) points to explosive feeding diversification of modern whales (Neoceti). *Proceedings of the Royal Society B: Biological Sciences*, *284*(1861), 20170531.

13) Online Figure 10. Most of the skeletal anatomy for the new species is not known. How much postcranial anatomy is known from other species in the genus and also morphology of the lower jaw. It might be good to add which species were used to make this artist's reconstruction, as the new species is based on just a fragmentary specimen.
Reply: We have previously reported specimens of the genus with associated (partial) mandibles e.g. MUSM 171 in Benites-Palomino etl al. 2020, which is the basis of this reconstruction.

Reviewer #2 (Remarks to the Author):

This paper is a well-written, worthwhile addition to the literature on a bizarre group of cetaceans, the kogiids. It is a further example of the incredible fossils and the excellent work being done on them in Peru. The paper describes a new species of kogiid from the Scaphokogiinae subfamily, and it appears to have a very distinct set of morphological features which hint at a specialised feeding ecology. There are two main claims of the paper. First, is the extreme asymmetry of species. And second, the high degree of ventral flexibility:

1) My main issue with the paper is the justification used for the former main claim. The authors have used PCA to quantify asymmetry, but this is not what PCA does. Rather, it is quantifying the shape variation in the craniofacial region. This may well be due to asymmetry, but it could just be differently shaped. Similarly, saying S. huachafo is more asymmetrical than all other taxa is not quite right, it is simply in a distinct region of morphospace. I would recommend considering at least one method of directly quantifying asymmetry to be employed. At a minimum, the differences in the morphology described by the landmark configurations should be described and compared to highlight if it is indeed due to increased asymmetry. Some of this detail is already in the extended description. Figure 3 is a good start in visualising the differences but I believe the use of the R package landVr to show landmark variation would be very useful indeed.

Reply: We have commented on general modifications to the paper, particularly on the morphometric and asymmetry analysis on the section on top of revision comments. Figure have also been improved in order to facilitate the interpretation of our more exhaustive analyses (e.g. Fig. 3)

2) Lastly, a very speculative question from me is whether the high diversity of this genus seen in this region could be potentially due to some taxa actually representing an ontogenetic series or sexual dimorphism?

Reply: This is also partly addressed into the "general modifications" section. Regarding ontogenetic series, it is worth noting that most of our specimens represent adult stages, with the exception of a juvenile/sub-adult previously published: *Scaphokogia* sp. MUSM 978 (Benites-Palomino et al. 2022). Furthermore, observations of stranded specimens indicate that most cranial anatomical features are already present in prenatal individuals, suggesting that differences in cranial morphology develop before birth (Benites-Palomino et al., *unpublished*).

3) Overall, this is a very solid paper, and once the recommendations below and in the attached PDF are made, I look forward to seeing it in the literature. Well done to all the authors

Reply: we thank the reviewer for all the suggestions and this positive feedback

Reviewer #3 (Remarks to the Author):

Dear colleagues,

The manuscript by Aldo Benites-Palomino et al. presents the interesting and important discovery of an unusual extinct toothed whale showing exceptionally developed cranial asymmetry combined with other unusual evolutionary adaptations. I believe this is a spectacular specimen which improves our knowledge on extreme manifestations of animal evolution, and it will be of broad public interest and attention and a source of inspiration for applied science. Therefore, it is worth to be published in Communications Biology.

The suggestions which should be considered before the publication:

1) The Results section in the main text is reduced by the diagnosis with the remarks which are the small part of the description, while the description itself is in the supplementary information. This should be reorganized: the diagnosis should be clearly separated following the ICZN requirements and have a clear differential formulation, and the descriptive elements (starting from the size of the specimen) should follow it and include the references to the supplementary files. Also, the description in the main text should be expanded to give an overall idea of the specimen. Given that its current version contains only 200 lines, most of it can be placed in the main text with some editing.

   Reply: As suggested by reviewer, we have modified the section accordingly: the section has been modified into a Differential Diagnosis and some features have been also modified in the Supplementary Information.

2) Quantification of directional asymmetry should be done in the ways comparable with previous studies, e.g. the partial Procrustes distance of the asymmetric component of shape to the corresponding symmetric component (Laeta et al. 2023), the sum of radii (Coombs et al. 2020) and lateral displacement of landmarks (Huggenberger et al. 2017). See also Ness (1967) and Hirose et al. (2015). At least, several criteria should be considered. Specifically, it should be clearly shown that the directional asymmetry in the new species exceeds all the previously known records by all the measured criteria.
   Reply: Since this is a comment also pointed out by other reviewer, please refer to 'General Comments on the Manuscript: Justification and interpretation of morphometric and asymmetry analyses'

3) Clinorhynchy (l. 221-223) and hyperostosis of the rostrum (l. 225-227) should be extensively discussed for their possible functions. Relation of clinorhynchy to benthic feeding in toothed whales has been discussed in several works from Monteiro-Filho et al. 2002 to Galatius et al. 2020 (see some refs below). Equally, rostral ossification should be compared at least with that of beaked whales with its possible evolutionary implications (e.g., Cozzi et al. 2010; Lambert et al. 2011). In overall, here the great bulk of previous comparative anatomical research should be discussed.

   Reply: we have included additional comments regarding this in the discussion.

4) Unique sensory adaptations (l. 233-235): This is a productive idea, potentially the core discovery of the research, that should be expanded and clearly communicated as working hypotheses. What kind of unique echolocation adaptations or, if other than echolocation, what kind of sensory developments? How the heavy

rostrum could contribute to these adaptations? How the deep supracranial basin could be involved? (Cranford et al. 2008, etc). Additionally, the overall idea of prey and other object search under the substrate should be better discussed (e.g., Rossbach and Herzing 1997; Mann et al. 2008), maybe with other suggestions if interesting (Gol'din 2014).

Reply: we have included additional comments regarding this in the discussion.

5) Methods and presentation. Full record of all the statistical data should be included as the supplementary data. TNT: Traditional Search and New Technologies algorithms: all the details should be added. All the resulting clade values and indices should be added to the figure depicting the phylogeny. PCA: PACA should be added to the PhyloPCA, and all the statistics should be provided. In addition, the interpretation of the PCA results (ED Fig 6) does not correspond to the authors' main idea: the species showing the same trends as the new one are Simocetus, Phocoena and Ankylorhiza, all showing little to no asymmetry (especially, Simocetus). There should be another explanation for the data.

Reply: As stated in our methods, the main driver for the phylogenetic analyses was PAUP, TNT was used as a secondary software to validate our results. We have included references to this nevertheless in the methods. Regarding the PCA/PaCA there is a section in the beginning that deals with this. In order to further clarify Figure 3 has been modified to include the morphospace and the phyloPCA. It is worth noting that *Simocetus* (besides *Ankylorhiza*) is one of the few fully preserved stem Odontoceti whose skull has been fully available o repositories and since its one of the earliest diverging odontoctes of course it appears as an outlier since the analysis was focused on Odontoceti (no Archaeoceti were included in the analysis due to several constrains).

Thursday, 28.05.2026

Editor

Communications Biology

Dear Dr. Michele Repetto

Associate Editor *Communications Biology*

Thank you for the opportunity to submit a revised version of our paper entitled "EXTREME CRANIAL ASYMMETRY IN EXTINCT BENTHIC FEEDING SPERM WHALES" to Communications Biology. We are grateful for your editorial work and for the time invested in evaluating our manuscript. We also thank the referees for their detailed and constructive comments.

We agreed with all reviewer comments and revised the manuscript accordingly. Detailed, point-by-point responses to the reviewers' comments are provided below. A tracked-changes version of the manuscript is included as a separate file to facilitate comparison with the original submission.

We hope that the revised manuscript is now suitable for publication in *Communications Biology*, and we reiterate our sincere gratitude for the time and effort invested in the review process.

Sincerely and on behalf of my coauthors,

Aldo Benites Palomino

Reviewer #1 (Remarks to the Author):

The authors have improved their manuscript with their responses to the various reviewers. I will not comment on the specifics brought up by other reviewers (e.g., on taxonomic description and quantification of skull asymmetery) and leave this to editors and other reviewers to weigh in. For the minor points that I raised, the authors generally addressed all of my concerns which mainly had to do with qualifying results just a bit. For the point on snout being a different module and being more 'plastic', where the authors note on line 294 that, "The rostrum was not landmarked due to its high plasticity, as this structure responds to modular evolution, primarily related to the specific feeding ecology of each species", the authors should add the reference that they provided in response to reviews here (Boessenecker et al.) and perhaps other published papers to back up this point, which is not obvious and should be referenced by prior work. I think maybe 'evolutionarily labile' might be a better term to use here rather than 'plastic' as plasticity in biology often refers to the ability of an organism to adjust phenotypically to different environmental conditions such that morphology depends on environment, while it seems the authors here are referring to the fact that the snout responds evolutionarily over phylogenetic scales and changes freely (among species) to fit ecological circumstances. At any rate, this work is very interesting and the authors should be commended for their efforts.

Reply: *We thank the reviewer for the positive evaluation of the revised manuscript and for the constructive final suggestions. Following the reviewer's recommendation, we added the relevant literature reference (Boessenecker et al.) to support the statement regarding rostral evolution and modularity. We also replaced the term "plasticity" with wording more appropriate to evolutionary change across phylogenetic scales.*

Reviewer #2 (Remarks to the Author):

The revised version of this manuscript does very well at addressing the reviewer comments. Other than the small suggested changes in the attached pdf, I believe that this article is now ready for publication. Well done to the authors.

Reply: *We thank the reviewer for the positive assessment of the revised manuscript. Suggested minor changes were implemented accordingly.*

Reviewer #3 (Remarks to the Author):

The revised manuscript has been well improved to the extent that now it can be published as is.

Reply: *We thank the reviewer for the positive evaluation of the revised manuscript and for considering the manuscript suitable for publication.*

EXTREME CRANIAL ASYMMETRY IN EXTINCT BENTHIC FEEDING SPERM WHALES

Aldo Benites-Palomino,[1,2,*] Marcelo R. Sánchez-Villagra[1], Florencia Paolucci[3], Jorge Vélez-Juarbe[4], Alberto Collareta[5], Apolline Alfsen[6], Alton Dooley[7], Dan Omura[8], Mario Urbina[2], Rodolfo Salas-Gismondi[2,9] & Gabriel Aguirre-Fernández[1]

[1]Department of Paleontology, University of Zurich, Karl-Schmid-Strasse 4 Zürich 8006, Switzerland.

[2]Departamento de Paleontología de Vertebrados, Museo de Historia Natural-Universidad Nacional Mayor de San Marcos, Avenida Arenales 1256, Lima 11, Peru.

[3]División Paleontología Vertebrados, Unidades de Investigación Anexo Museo, Facultad de Ciencias Naturales y Museo, UNLP, CONICET, Av. 60 y 122, B1900AVW La Plata, Argentina

[4]Department of Mammalogy, Natural History Museum of Los Angeles County, 900 Exposition Blvd., Los Angeles, California 90007, U.S.A.,

[5]Dipartimento di Scienze della Terra, Università di Pisa, via Santa Maria 53, 56126, Pisa, Italy;

[6]Department of Evolutionary Morphology, Museum für Naturkunde, Leibniz-Institut für Evolutions- und Biodiversitätsforschung, Berlin, Germany

[7]Western Science Center, 2345 Searl Parkway, Hemet, CA 92543

[8]Centro de Desarrollo para la Educación Lima Nikko. Asociación Peruano Japonesa. Av. Gregorio Escobedo 803, Residencial San Felipe, Jesús María; Lima, Perú

[9]Facultad de Ciencias y Filosofía/ Laboratorios de Investigación y Desarrollo, Universidad Peruana Cayetano Heredia, Lima, Peru

*Corresponding author: aldo.benitespalomino@uzh.ch

Abstract

Fossils offer critical insights into morphological changes beyond those observed in extant species and, in rare instances, unveil evolutionary novelties. Toothed cetaceans exhibit pronounced cranial asymmetry, particularly linked to their sound-emitting forehead organs. Among these, pygmy sperm whales (Kogiidae) display some of the most extreme cranial asymmetries known. Here, we describe *Scaphokogia huachafo* sp. nov., a new pygmy sperm whale species from the Late Miocene Pisco Formation, Peru. This species exhibits extreme craniofacial asymmetry not only ~~limiting~~ to the external bony nares, but the surrounding bony structures. Geometric morphometric analyses of facial bone structure reveal *S. huachafo* as exhibiting the most pronounced facial asymmetry documented among cetaceans, and possibly among mammals. Distinct morphological traits include a robust, ventrally oriented tubular rostrum composed of dense pachyostotic bone reinforced by ossified cartilage. Morphological reconstruction of the atlanto-occipital joint suggests enhanced ventral neck flexibility, optimized for benthic foraging behaviours. This extreme cranial morphology likely represents a unique adaptation, with no direct modern analogues, greatly expanding the known diversity of cetacean skull architecture. *S. huachafo* occupied now-extinct ecological niches, feeding near the bottom of the shallow coasts of Peru during the late Miocene~~, a crucial moment for oceans evolution~~.

MAIN TEXT

Introduction

Cranial asymmetry in vertebrates is predominantly associated with pathologies or malformations[1,2]. However, in some cases, asymmetry emerges as an evolutionary innovation closely linked to the development of specific ecological traits. The most well-known example is found in flatfishes (Pleuronectiformes), where both eyes are located on one side of the head[3]. Among tetrapods, certain owl species (Strigiformes) exhibit asymmetric skulls, which enhance sensory functions by improving auditory capabilities due to the differential positioning of their ears[4,5], thereby increasing hunting efficiency during crepuscular hours. In mammals, cetaceans provide the most notable example of asymmetry. This condition is associated with adaptations to underwater life, such as directional hearing in archaeocetes[6,7] (early-diverging cetaceans) and high-frequency sound production and echolocation in Odontoceti (toothed cetaceans), which enhance communication and prey acquisition skills[8]. Directional asymmetry, characterized by differences between the two sides of the body, reaches its most extreme manifestation in the skulls of odontocetes across cetaceans[9]. Amongst them, sperm whales (Physeteroidea) exhibit this feature to an extraordinary degree[10,11]. Their skulls show a remarkable leftward deviation of the right facial bones, such as the premaxilla and nasal bones[12]. Additionally, sperm whales have developed a dorsally excavated basin, known as the supracranial basin, which houses their enlarged nasal complex, composed mainly of fatty forehead organs[13,14].

Directional cranial asymmetry and the development of the supracranial basin are both observed since the earliest stages of sperm whale evolution. These traits are evident even in some of the oldest known physeteroids, such as *Raphicetus*[15] as well as in raptorial sperm whales like *Acrophyseter* and *Zygophyseter*[16–18]. The fossil record further reveals significant variation in the cranial patterning of extinct sperm whales, indicating that nasal structure development was unique to each physeteroid species[15]. Diversity in cranial shape disparity suggests that extinct sperm whales occupied a broader range of ecological niches in pelagic and epipelagic waters during the Miocene. These niches ranged from small piscivorous or teuthophagous species to large apex predators. In contrast, modern physeteroids are specialised suction feeders with a diet primarily consisting of cephalopods captured at great ocean depths[19–22]. Present-day sperm whales differ notably in body size and the structure of their supracranial basins. *Physeter macrocephalus*, the largest extant odontocete, is characterised by an undivided supracranial basin[13,14], while species of *Kogia* (Kogiidae) possess dorsally compartmentalised skulls with independent cavities housing multiple nasal organs[23,24]. The Miocene fossils of Peru showcase a diverse array of kogiid sperm whales, especially those from the genus *Scaphokogia*, which are distinguished by their highly specialised craniofacial anatomy, including a robust, tubular rostrum[25,26].

Here, we describe a new pygmy sperm whale from the Late Miocene Pisco Formation (Peru). This new species is defined by a prominent, downward-oriented tubular rostrum, primarily constructed from pachyostotic bone and ossified cartilage. The cranial structure reveals an unprecedented cranial asymmetry among cetaceans, as demonstrated through Geometric Morphometric analyses of the skull patterning. Additionally, we reconstructed the range of motion of the atlanto-occipital joint (connecting the skull and atlas) for this genus, proposing an unusual feeding strategy for these peculiar sperm whales. Together, these findings indicate that the new species exhibits a highly derived cranial morphology, suggesting it was a highly specialized benthic feeder with no modern equivalent. The new species described here pushes the boundaries of cranial architecture in cetaceans (and mammals) and implies the existence of ecological niches once exploited by marine mammals that have no modern equivalents.

Systematic Palaeontology

Cetacea Brisson, 1792

Odontoceti Flower, 1867

Physeteroidea Gray, 1821

Kogiidae Gill, 1871

Scaphokogiinae Muizon, 1988

Scaphokogia Muizon, 1988

Species included— *Scaphokogia cochlearis* Muizon, 1988, *Scaphokogia totajpe* Benites-Palomino et al. 2020, *Scaphokogia huachafo* sp. nov.

Emended Diagnosis— Small to medium-sized physeteroids, with estimated body lengths ranging from 220-280cm. Diagnosed by the following combination of characters: mesorostral groove completely

closed (c.6[2]), tubular rostrum with a height-to-width ratio taken ad midlength exceeding 0.8 (c.8[1]), dorsoventrally-thickened vomer at the anterior tip of the rostrum (c.9[1]), rostrum with a rectangular outline in dorsal view due to its predominantly parallel lateral margins (c.10[1]), constriction of the premaxilla indicated by the premaxilla-maxilla suture being roughly anteriorly directed (c.11[1]), transversely-thickened mesethmoid in the nasal septum (c.22[1]), and deep alveoli with rudimentary septa (c.51[1]). Additionally, the supracranial basin of *Scaphokogia* is deeper over the right orbit, where it can even overhang the orbit as in *S. cochlearis*.

Scaphokogia huachafo sp. nov.

(Fig. 1; Extended Data Figs. 1-3)

Etymology— The specific name *huachafo*, commonly used in Peru to describe something "bizarre," "odd," or "extravagant," highlights the distinctive cranial features of the species.

Holotype–The holotype skull of *Scaphokogia huachafo* gen. et sp. nov. MUSM 972 is mostly complete, except for the squamosal and lateralmost portion of the frontal on the left side of the cranium (Extended Data Figs. 1-3). It corresponds to a fully grown adult as indicated by the well-ossified cranial bones and the fully closed or ankylosed cranial sutures

Locality and Age–MUSM 972 was collected in Aguada de Lomas, Sacaco basin (Arequipa, Peru). The fossil-bearing horizon was deposited between 6.3-5.6 Ma., during a shallow marine transgression[27,28].

Phylogenetic framework–Our results undoubtfully recover *S. huachafo* deeply nested within Kogiidae as a highly derived member of the Scaphokogiinae clade and the genus *Scaphokogia* (Fig. 2; Extended Data Fig. 4). *Scaphokogia huachafo* share morphological aspects with others member of the genus *Scaphokogia*, such as a tubular rostrum, the inferred hypertrophied nasal complex, and the bowl-like supracranial basin. Our results are consistent with previous works showing a high support of Kogiidae as a clade an in its subdivision into subfamilies with their respective composition: Scaphokogiinae (*Scaphokogia* spp., *Platyscaphokogia*) and Kogiinae (*Nanokogia*, *Praekogia*, *Koristocetus*, *Pliokogia*, *Kogia*)

Diagnosis and remarks– The holotype specimen of *Scaphokogia huachafo* exhibits a condylobasal length of 632 mm and a maximum anteroposterior length of 701 mm, measured from the posterior tip of the nuchal crest to the anterior tip of the rostrum. *Scaphokogia huachafo* is identified as a Kogiidae due to absence of nasal bones, a rostrum formed by the premaxillae, maxillae, and vomer, the lack of premaxillary teeth, the absence of a posteriorly widened right premaxilla, the extension of the right maxilla reaching the sagittal plane of the skull, and a ventrally extended postorbital process of the frontal bone. The rostrum of the holotype specimen is notably robust, significantly larger, and more massive compared to *S. totajpe* and *S. cochlearis*, due to the extensive development of pachyostotic bone and the partial ossification of the mesethmoid. The rostrum is oriented anteroventrally, forming a 31° angle with the main cranial (or body) axis. Ventrally, there are eight large and well-defined maxillary alveoli that transition posteriorly into a deep alveolar groove. In adults of other members of the genus, alveoli are partially reabsorbed and have mostly filled with spongy bone, suggesting that functional teeth are greatly reduced. Due to the size of the alveoli in *S. huachafo*, it is possible that teeth were present during an earlier ontogenetic stage and were subsequently reabsorbed or lost. In the facial region, both external bony nares are displaced towards the left side of the skull, with the left naris being significantly larger than the right—both unique conditions among sperm whales. The left antorbital process is better preserved than the right, forming a lamina-like structure that extends dorsally to the rostrum and posteriorly to the postorbital process, parallel to the anterior wall of the supracranial basin. The left antorbital region extends more anteriorly than the right, highlighting the skull's overall asymmetry. Taphonomic distortion was ruled out as there is no evidence of deformation in other regions of the skull. The anterior wall of the supracranial basin is much more robust in *S. huachafo* due to the pachyostotic growth of the right premaxilla. Posteriorly, the supracranial basin is proportionally smaller than in *S. totajpe* or *S. cochlearis*, but deeper when compared with the latter, displaying a mostly circular outline and being restricted to the facial region of the skull. The asymmetric deepening of the supracranial basin extends into the intracranial cavity, which is more dorsoventrally compressed on its right half, likely affecting brain shape, as in extant *Kogia*. The temporal fossa is longer anteroposteriorly than it is high, similar to the condition observed in *S. totajpe*, contrasting with the enlarged fossa of *S. cochlearis*. The nuchal crest significantly overhangs the occipital condyles, which are proportionally smaller than in other *Scaphokogia* species.

Discussion

Among mammals, directional cranial asymmetry is a distinctive feature of most odontocetes, linked to sound production, biosonar, and the structures involved in these processes[29]. In early diverging clades, such as physeteroids, ziphiids, and platanistoids, the asymmetry is pronounced, as evidenced by the

distribution of their bones and the enlargement of nasal organs[10]. Asymmetry in head tissues and associated structures in physeteroids (e.g., the spermaceti, melon, and surrounding forehead organs) is also evident from early foetal stages[11,12,29], supporting the idea of a derived developmental pattern distinct from other toothed cetaceans (Synrhina).

Several morphometric analyses have quantified cranial asymmetry in cetaceans[10,29–32], including sperm whales; however, the unique characteristics and evolutionary novelties in the skulls of these animals have not been fully considered. This is particularly relevant as some structures (e.g., nasal bones) are lost in sperm whales[23,24], while others (e.g., sagittal facial crest) are unique to this clade. For this reason, our craniofacial asymmetry (CFA) analyses focused on Physeteroidea and ~~including~~ early odontocetes (Extended Data Fig. 5) In our initial CFA analyses, physeteroids occupied a different region of the phylomorphospace than Synrhina or stem odontocetes but were somewhat close to Platanistidae (Fig. 3; Extended Data Fig. 6). This proximity could be related to the development of a circumnarial basin in some platanistids[33] (e.g., *Pebanista*), likely convergent with the supracranial basin of physeteroids. However, within the same analysis, Kogiidae formed a cluster at an extreme position of the PCA, separate from other sperm whales. This separation is primarily associated with the much greater posterior extension of the right premaxilla and the more pronounced size difference between the external bony nares in kogiids[34].

In a subsequent analysis, which included only physeteroids, the CFA revealed a subdivision of Kogiidae into its two main clades which follow different evolutionary paths: Kogiinae and Scaphokogiinae. The most extreme conditions are found in two members of these clades: *Kogia breviceps* and *Scaphokogia huachafo*. These clades are easily distinguishable based on the depth and extent of their supracranial basin and the presence of two or three cavities within this basin[23,25,35], created by the sagittal facial crest. Within the supracranial basin of *Kogia*, the premaxillary fossa and the right maxillary fossa house most of the organs of the nasal complex[24,36], which have higher capabilities for propagating and reflecting sound waves[37,38], restricting the left maxillary fossa's role as a supplementary anchor site for the enlarged facial muscles[39], mainly responsible for sound modulation. It is not possible to assess the in-vivo extent of the nasal organs in *Scaphokogia*, as the preservation of soft tissues in deep time is extremely rare. However, the attachment sites of these structures clearly indicate that the sound production system was well-developed and distinct from that of *Kogia*.

One of the most distinctive features of *Scaphokogia* spp. is its massive tubular rostrum. Additionally, all species within this genus exhibit ventral deviation of the rostrum (klinorhynchy)[40], with the angle of deviation varying across species: from a low range in *S. cochlearis* (~16-21º) to a moderately high angle in *S. totajpe* (27º) and reaching 31º in *S. huachafo* relative to the main cranial axis (Extended Data Fig. 9). The rostrum of *S. huachafo* is further characterized by elongation, bulging due to the pachyostotic bone growth, and partial ossification of the mesorostral cavity. These features likely shifted the centre of gravity of the animal forward. Bone mass increase in the skull and/or skeleton in marine mammals is usually associated with benthic feeding (e.g. sirenians, odobenines, archaeocetes) by providing greater hydrostatic control[41–43].

An additional specimen identified as *Scaphokogia* sp. (MUSM 171) preserves the atlas, allowing reconstruction of the range of motion (ROM) of the atlantooccipital joint[44]. When the neck was fully flexed dorsally, the rostrum formed a ventral angle of 22° with the main body axis, and at maximum ventral flexion, the angle increased to 55° (Fig. 4). This suggests that the rostrum was permanently oriented ventrally. The skull asymmetry of *Scaphokogia* spp. is also evident in the occipital condyles: the right condyle is broader than the left one and is more dorsolaterally oriented, while the left one is larger but slightly more ventrally positioned (Extended Data Fig. 7). The corresponding articular facets of the atlas are also asymmetrical, with the right facet projecting slightly ventrally, further emphasized by the anteroposterior thickening of the right half of the bone given to it a trapezoidal profile. Because of this the movement of the atlanto-occipital joint most likely followed a curved trajectory rather than a straight line.

Collectively, these features suggest that the skull of *Scaphokogia* was not aligned with the main body axis but exhibited a ventrolateral torsion during swimming or resting. The articulation between the skull and neck of the *Scaphokogia* genus apparently did not function strictly in a single vertical plane but likely involved some degree of torsion, possibly related to its unique feeding mode. The combination of the pronounced skull asymmetry and significant ventral deflection (klinorhynchy) of the rostrum indicates a highly specialized feeding ecology for *S. huachafo* and other members of the genus *Scaphokogia*. Klinorhynchy is generally considered an energy-efficient adaptation for benthic feeding, as seen in sirenians. A plausible hypothesis is that the pronounced asymmetry in this animal may have served an enhanced sensory function. The concave surface of both premaxillae suggests the presence of a large melon, which, along with other forehead structures, may have functioned as a benthic sonar, facilitating benthic foraging. Additional indirect evidence includes the greatly hyperossified ventral

surface of the skull (Extended Data Fig. 8), which may have aided hydrostatic control when foraging near the seafloor.

Benthic feeding ecologies have been previously proposed for some ancient cetaceans like protocetids, as well as other coeval cetaceans from the Pisco Formation, particularly the walrus-dolphin *Odobenocetops*, which also exhibits a ventrally oriented skull and rostrum[45,46]. Most bottom-feeding marine mammals rely on vision and specialized orofacial structures with varying degrees of motor and sensory functions for detecting and acquiring food, as seen in *Odobenocetops*. Since *Scaphokogia* appears to lack specialized orofacial structures, we propose that it instead evolved unique echolocation adaptations to facilitate prey detection and acquisition in soft substrates. Finally, the co-occurrence of multiple genera of specialized benthic feeders in Late Miocene ecosystems reflects the higher abundance of benthic invertebrate taxa and the relatively stable environmental conditions of the region. This stability, especially in shallow marine and coastal habitats, lasted until the Pliocene[27,28], when the final marine turnover restructured the oceans into their modern configuration.

Conclusion

Scaphokogia huachafo represents a new species of pygmy sperm whale from the Late Miocene Pisco Formation in Peru, one of the most bizarre fossil cetaceans known so far due to its highly idiosyncratic cranial architecture. *S. huachafo* is recognised as the most derived species of the genus *Scaphokogia* in our subsequent phylogenetic analyses, displaying the typical cranial characters of the genus but in its most derived state. Furthermore, 3D morphometric analyses of sperm whales and other odontocetes indicate that this new species exhibits a unique craniofacial asymmetry among cetaceans, due to the disproportionate positioning of the external bony nares and premaxillary bones. Additionally, reconstructions of the atlantooccipital joint in this genus, along with the ventrally oriented rostrum, indicate that this species was able to ~~significantly~~ flex its skull ventrally. This, combined with the massive tubular rostrum suggests that this animal was a specialized benthic feeder, capable of sweeping through the bottom of the shallow coastal environments of the Late Miocene. *Scaphokogia huachafo* represents one of the most highly derived morphologies ever attained by sperm whales in the past, occupying an ecological niche with no modern parallel across odontocetes, shortly before the final turnover that gave rise to our modern marine mammal fauna.

Methods

Phylogenetic analyses– To assess the phylogenetic relationships of *Scaphokogia huachafo* within Kogiidae, a phylogenetic matrix was constructed using Mesquite 3.70[47], comprising 68 morphological characters and 38 operational taxonomic units. This matrix was based on previous research on Physeteroidea systematics[15,16,23,25,35,48–51], yet several characters were re-defined due to ambiguous coding (Supplementary Information S4). The cladistic parsimony analyses were performed in PAUP 4.0a169[52] taking characters as unordered and ordered. Because of the high number of poorly supported nodes (less than 50% bootstrap support) and polytomies resulting from the first parsimony analysis, additional analyses were performed by down-weighting of homoplastic characters with k values of 2, 3, 10, 20, and 40. Because the topology remained unmodified, the lowest value was kept (K=3). The statistical support for the results were calculated on the basis of 1000 bootstrap replicates. To further test the consistency of our results, the same analyses were also performed in TNT 1.6[53] using the Traditional Search and New Technologies algorithms.

Craniofacial asymmetry– To assess the patterning and degree of asymmetry specific to the facial region of the skull in cetaceans, a series of morphometric analyses were conducted. Since most of previous studies that have dealt with morphometrics in cetacean skulls were focused on Synrhina[10,29,30,32], a new landmarking protocol was developed mostly focusing on sperm whales and taking in account their morphological peculiarities. The set of landmarks was collected in the software MorphoDIG[54] and included 37 points, consisting of two semilandmark curves (each with 15 points) taken on each premaxilla from the level of the antorbital notch, and seven landmarks taken on the preorbital process of the frontal, the antorbital notch, and the posterior end of the nasal septum (Extended Data Fig. 5). The rostrum was not landmarked due to its high plasticity, as this structure responds to modular evolution, primarily related to the species' ecology. Additionally, no landmarks were taken on the nasal bones as these are completely missing in kogiids. Furthermore, since at least two scaphokogiines (*Scaphokogia huachafo* MUSM 972 and *Scaphokogia totajpe* MUSM 973) have not preserved the entirety of the supracranial basin, no landmarks were taken on the maxillae. The dataset consisted of 31 taxa, including 11 sperm whales, 4 stem odontocetes and 16 Synrhina. The craniofacial asymmetry (CFA) analyses were performed in R 4.3.2[55] using the package geomorph[56], calculating the phylomorphospace and PhyloPCA. An additional subsequent analysis was performed in a subset only including members of the Physeteroidea.

Benthic adaptations–To measure the range of dorsoventral motion (ROM) allowed by the atlantooccipital joint (the articulation between the occipital condyles of the skull and the atlas bone), the skull and atlas are positioned in their most ventrally flexed and dorsally extended positions. The angle formed by the rostrum and the main body axis is then measured. The ROM was measured in *Scaphokogia* sp. MUSM 171, the only specimen of the genus with a preserved atlas (Extended Data Fig. 7). Unlike other odontocetes, where the rostral orientation is measured using the plane formed between the distal tip of the premaxillae and the dorsal margin of the orbit, in *Scaphokogia* sp. MUSM 171, the rostral plane is averaged using the angles from the roof and floor of the rostrum. These measurements were incorporated into the dataset of Okamura and Fujiwara[44], expanding the dataset to include 31 species of cetaceans, including the extant sperm whales *K. breviceps* and *Physeter macrocephalus*.

Bone development patterning–The holotype specimen of *Scaphokogia totajpe* MUSM 973 was CT-scanned at Materials, Research & Technology facilities (Lima, Peru). The data was first handled in Dragonfly 3D (Version 2024.1 for Windows) to orientate the skulls. Three slices were extracted and segmented: one from near the distal tip of the rostrum (Extended Data Fig. 8), another from the midlength of the rostrum, and the final one at the level of the external bony nares. Binarized tiffs were exported for performing local thickness analyses. Thickness maps and measurements of the slices were completed using the Local Thickness tool in Fiji (v.1.54f)[57].

Data Availability

Code used in this paper is available in https://github.com/aldombp/Oddwhale

Surface scans will be available at Morphomuseum: http://dx.doi.org/10.18563/m3.sf.977

References

1. Captier, G. *et al.* Plagiocephaly: morphometry of skull base asymmetry. *Surg. Radiol. Anat.* **25**, 226–233 (2003).
2. Netherway, D. J. *et al.* Three-Dimensional Computed Tomography Cephalometry of Plagiocephaly: Asymmetry and Shape Analysis. *Cleft Palate. Craniofac. J.* **43**, 201–210 (2006).
3. Friedman, M. The evolutionary origin of flatfish asymmetry. *Nature* **454**, 209–212 (2008).
4. Ocurrence and independent evolution of bilateral ear asymmetry in owls and implications on owl taxonomy. *Philos. Trans. R. Soc. Lond. B Biol. Sci.* **280**, 375–408 (1977).
5. Pecsics, T., Laczi, M., Nagy, G., Kondor, T. & Csörgő, T. Analysis of skull morphometric characters in Owls (Strigiformes). *Ornis Hung.* **26**, 41–53 (2018).
6. Fahlke, J. M. & Hampe, O. Cranial symmetry in baleen whales (Cetacea, Mysticeti) and the occurrence of cranial asymmetry throughout cetacean evolution. *Sci. Nat.* **102**, 1–16 (2015).
7. Fahlke, J. M., Gingerich, P. D., Welsh, R. C. & Wood, A. R. Cranial asymmetry in Eocene archaeocete whales and the evolution of directional hearing in water. *Proc. Natl. Acad. Sci.* **108**, 14545–14548 (2011).
8. Fordyce, R. E. & Muizon, C. de. Evolutionary history of cetaceans: a review. *Second. Adapt. Tetrapods Life Water* 169–233 (2001).
9. Compagnucci, C., Fish, J. & Depew, M. J. Left–right asymmetry of the gnathostome skull: Its evolutionary, developmental, and functional aspects. *genesis* **52**, 515–527 (2014).
10. Coombs, E. J., Clavel, J., Park, T., Churchill, M. & Goswami, A. Wonky whales: The evolution of cranial asymmetry in cetaceans. *BMC Biol.* **18**, (2020).
11. Klima, M. *Development of the Cetacean Nasal Skull*. vol. 149 (Springer Science & Business Media, 1999).
12. Klima, M. Cetacean phylogeny and systematics based on the morphogenesis of the nasal skull. *Aquat. Mamm.* **21**, 79–79 (1995).
13. Huggenberger, S., André, M. & Oelschläger, H. H. A. The nose of the sperm whale: Overviews of functional design, structural homologies and evolution. *J. Mar. Biol. Assoc. U. K.* **96**, 783–806 (2016).
14. Cranford, T. W. The sperm whale's nose: Sexual selection on a grand scale? *Mar. Mammal Sci.* **15**, 1133–1157 (1999).
15. Lambert, O., Muizon, C. de, Urbina, M. & Bianucci, G. A new longirostrine sperm whale (Cetacea, Physeteroidea) from the lower Miocene of the Pisco Basin (southern coast of Peru). *J. Syst. Palaeontol.* **18**, 1707–1742 (2020).
16. Lambert, O., Bianucci, G. & Muizon, C. de. Macroraptorial sperm whales (cetacea, odontoceti, physeteroidea) from the miocene of peru. *Zool. J. Linn. Soc.* **179**, 404–474 (2017).
17. Bianucci, G. & Landini, W. Killer sperm whale: A new basal physeteroid (Mammalia, Cetacea) from the Late Miocene of Italy. *Zool. J. Linn. Soc.* **148**, 103–131 (2006).
18. Lambert, O., Bianucci, G. & Muizon, C. de. A new stem-sperm whale (Cetacea, Odontoceti, Physeteroidea) from the Latest Miocene of Peru. *Comptes Rendus - Palevol* **7**, 361–369 (2008).
19. Clarke, M. R. & Pascoe, P. L. Cephalopod Species in the Diet of a Sperm Whale (*Physeter catodon*) Stranded at Penzance, Cornwall. *J. Mar. Biol. Assoc. U. K.* **77**, 1255–1258 (1997).
20. Clarke, M. R., Martins, H. R. & Pascoe, P. L. The Diet of Sperm Whales (Physeter macrocephalus Linnaeus 1758) off the Azores. *Philos. Trans. R. Soc. B Biol. Sci.* **339**, 67–82 (1993).
21. Evans, K. & Hindell, M. A. The diet of sperm whales (Physeter macrocephalus) in southern Australian waters. *ICES J. Mar. Sci.* **61**, 1313–1329 (2004).
22. Beasley, I., Cherel, Y., Robinson, S., Betty, E. & Gales, R. Pygmy sperm whale (Kogia breviceps) stranding record in Tasmania, Australia, and diet of a single specimen. *Pap. Proc. R. Soc. Tasman.* **147**, 25–32 (2013).
23. Benites-Palomino, A. *et al.* Nasal compartmentalization in Kogiidae (Cetacea, Physeteroidea): insights from a new late Miocene dwarf sperm whale from the Pisco Formation. *Pap. Palaeontol.* **n/a**, (2021).
24. Thornton, S. W. *et al.* Morphology of the Nasal Apparatus in Pygmy (Kogia Breviceps) and Dwarf (K. Sima) Sperm Whales. *Anat. Rec.* **298**, 1301–1326 (2015).
25. Benites-Palomino, A., Vélez-Juarbe, J., Salas-Gismondi, R. & Urbina, M. *Scaphokogia totajpe*, sp. nov., a new bulky-faced pygmy sperm whale (Kogiidae) from the late Miocene of Peru. *J. Vertebr. Paleontol.* **39**, e1728538 (2020).
26. Muizon, C. de. Les vertébré s fossiles de la Formation Pisco (Perou). Troisième partie : les odontocètes (Cetacea, Mammalia) du Miocène. *É Ditions Rech. Sur Civilis.* **78**, 1 (1988).
27. Ochoa, D. *et al.* Age and provenance of the Mio-Pleistocene sediments from the Sacaco area, Peruvian continental margin. *J. South Am. Earth Sci.* **116**, 103799 (2022).
28. Ochoa, D. *et al.* Revisiting the Late Neogene chronostratigraphic and paleontological record of the Pisco Basin (Peru): Implications on the onset and evolution of modern marine-coastal Humboldt Current ecosystems. *Int. J. Earth Sci.* (2021).

29. Lanzetti, A., Coombs, E. J., Portela Miguez, R., Fernandez, V. & Goswami, A. The ontogeny of asymmetry in echolocating whales. *Proc. R. Soc. B Biol. Sci.* **289**, 20221090 (2022).
30. Laeta, M. *et al.* Cranial asymmetry in odontocetes: a facilitator of sonic exploration? *Zoology* **160**, 126108 (2023).
31. Churchill, M., Geisler, J. H., Beatty, B. L. & Goswami, A. Evolution of cranial telescoping in echolocating whales (Cetacea: Odontoceti). *Evolution* **72**, 1092–1108 (2018).
32. Vicari, D. *et al.* Ecomorphology of toothed whales (Cetacea, Odontoceti) as revealed by 3D skull geometry. *J. Mamm. Evol.* **30**, 475–491 (2023).
33. Benites-Palomino, A *et al.* The largest freshwater odontocete: a South Asian river dolphin relative from the Proto-Amazonia. *Sci. Adv.* (2024).
34. Schulte, H. von. The skull of Kogia breviceps Blainv. *Bull. Am. Mus. Nat. Hist.* **37**, 361 (1917).
35. Collareta, A. *et al.* A new physeteroid from the late Miocene of Peru expands the diversity of extinct dwarf and pygmy sperm whales (Cetacea: Odontoceti: Kogiidae). *Comptes Rendus Palevol* (2020) doi:10.5852/cr-palevol2020v19a5.
36. Karol, R., Litchfield, C., Caldwell, D. K. & Caldwell, M. C. Compositional topography of melon and spermaceti organ lipids in the pygmy sperm whale Kogia breviceps: Implications for echolocation. *Mar. Biol.* **47**, 115–123 (1978).
37. Song, Z. *et al.* Acoustic property reconstruction of a pygmy sperm whale (Kogia breviceps) forehead based on computed tomography imaging. *J. Acoust. Soc. Am.* **138**, 3129–3137 (2015).
38. Song, Z., Zhang, Y., Thornton, S. W., Li, S. & Dong, J. The influence of air-filled structures on wave propagation and beam formation of a pygmy sperm whale (Kogia breviceps) in horizontal and vertical planes. *J. Acoust. Soc. Am.* **142**, 2443–2453 (2017).
39. Schulte, H. von & Smith, M. de F. The external characters, skeletal muscles, and peripheral nerves of Kogia breviceps (Blainville). *Bull. Am. Mus. Nat. Hist. 3827-72 1918* **38**, 66 (1918).
40. Bianucci, G. & Gingerich, P. D. *Aegyptocetus tarfa* , n. gen. et sp. (Mammalia, Cetacea), from the middle Eocene of Egypt: clinorhynchy, olfaction, and hearing in a protocetid whale. *J. Vertebr. Paleontol.* **31**, 1173–1188 (2011).
41. Bianucci, G. *et al.* A heavyweight early whale pushes the boundaries of vertebrate morphology. *Nature* 1–6 (2023) doi:10.1038/s41586-023-06381-1.
42. de Buffrénil, V., Canoville, A., D'Anastasio, R. & Domning, D. P. Evolution of sirenian pachyosteosclerosis, a model-case for the study of bone structure in aquatic tetrapods. *J. Mamm. Evol.* **17**, 101–120 (2010).
43. Houssaye, A. 'Pachyostosis' in aquatic amniotes: a review. *Integr. Zool.* **4**, 325–340 (2009).
44. Okamura, T. & Fujiwara, S. The range of atlanto-occipital joint motion in cetaceans reflects their feeding behavior. *J. Anat.* **236**, 434–447 (2020).
45. Muizon, C. de. Walrus-like feeding adaptation in a new cetacean from the Pliocene of Peru. *Nature* **365**, 745–748 (1993).
46. Muizon, C. de & Domning, D. P. The anatomy of Odobenocetops (Delphinoidea, Mammalia), the walrus-like dolphin from the Pliocene of Peru and its palaeobiological implications. *Zool. J. Linn. Soc.* **134**, 423–452 (2002).
47. Maddison, W. P. & Maddison, D. R. Mesquite: a modular system for evolutionary analysis. Version 2.75. 2011. *URL Httpmesquiteproject Org* (2015).
48. Paolucci, F., Buono, M. R., Fernández, M. S., Marx, F. G. & Cuitiño, J. I. Diaphorocetus poucheti (Cetacea, Odontoceti, Physeteroidea) from Patagonia, Argentina: one of the earliest sperm whales. *J. Syst. Palaeontol.* **18**, 335–355 (2020).
49. Paolucci, F., Fernandez, M., Buono, M. R. & Cuitiño, J. I. 'Aulophyseter' rionegrensis (Cetacea: Odontoceti: Physeteroidea) from the Miocene of Patagonia (Argentina): a reappraisal. *Zool. J. Linn. Soc.* (2020).
50. Velez-Juarbe, J., Wood, A. R., De Gracia, C. & Hendy, A. J. W. Evolutionary patterns among living and fossil kogiid sperm whales: Evidence from the neogene of Central America. *PLoS ONE* **10**, 1–29 (2015).
51. Lambert, O. *et al.* A new platyrostrine sperm whale from the Early Miocene of the southeastern Pacific (East Pisco Basin, Peru) supports affinities with the southwestern Atlantic cetacean fauna. *Geodiversitas* **45**, 659–679 (2023).
52. Swofford, D. L. PAUP*: phylogenetic analysis using parsimony, version 4.0b10. *21 Libro* (2003) doi:citeulike-article-id:2345226.
53. Goloboff, P. A., Farris, J. S. & Nixon, K. C. TNT, a free program for phylogenetic analysis. *Cladistics* **24**, 774–786 (2008).
54. Lebrun, R. MorphoDig, an open-source 3D freeware dedicated to biology. in *IPC5 The 5th International Palaeontological Congress* (2018).
55. Team, R. C. A language and environment for statistical computing. *No Title* (2021).
56. Adams, D. C. & Otárola-Castillo, E. geomorph: an R package for the collection and analysis of geometric morphometric shape data. *Methods Ecol. Evol.* **4**, 393–399 (2013).
57. Schindelin, J. *et al.* Fiji: an open-source platform for biological-image analysis. *Nat. Methods* **9**, 676–682 (2012).

Acknowledgments

The present contribution represents the culmination of almost a decade of continuous work devoted to the study of fossil sperm whales from Peru. As such, we express our tremendous gratitude to the palaeontological teams of PIMUZ (Department of Paleontology, University of Zurich, Switzerland), STRI (Smithsonian Tropical Research Institute, Panama) and DPV-MUSM (Departamento de Paleontologia de Vertebrados, Museo de Historia Natural UNMSM, Peru) for their tremendous support and aid in discussions during these years.

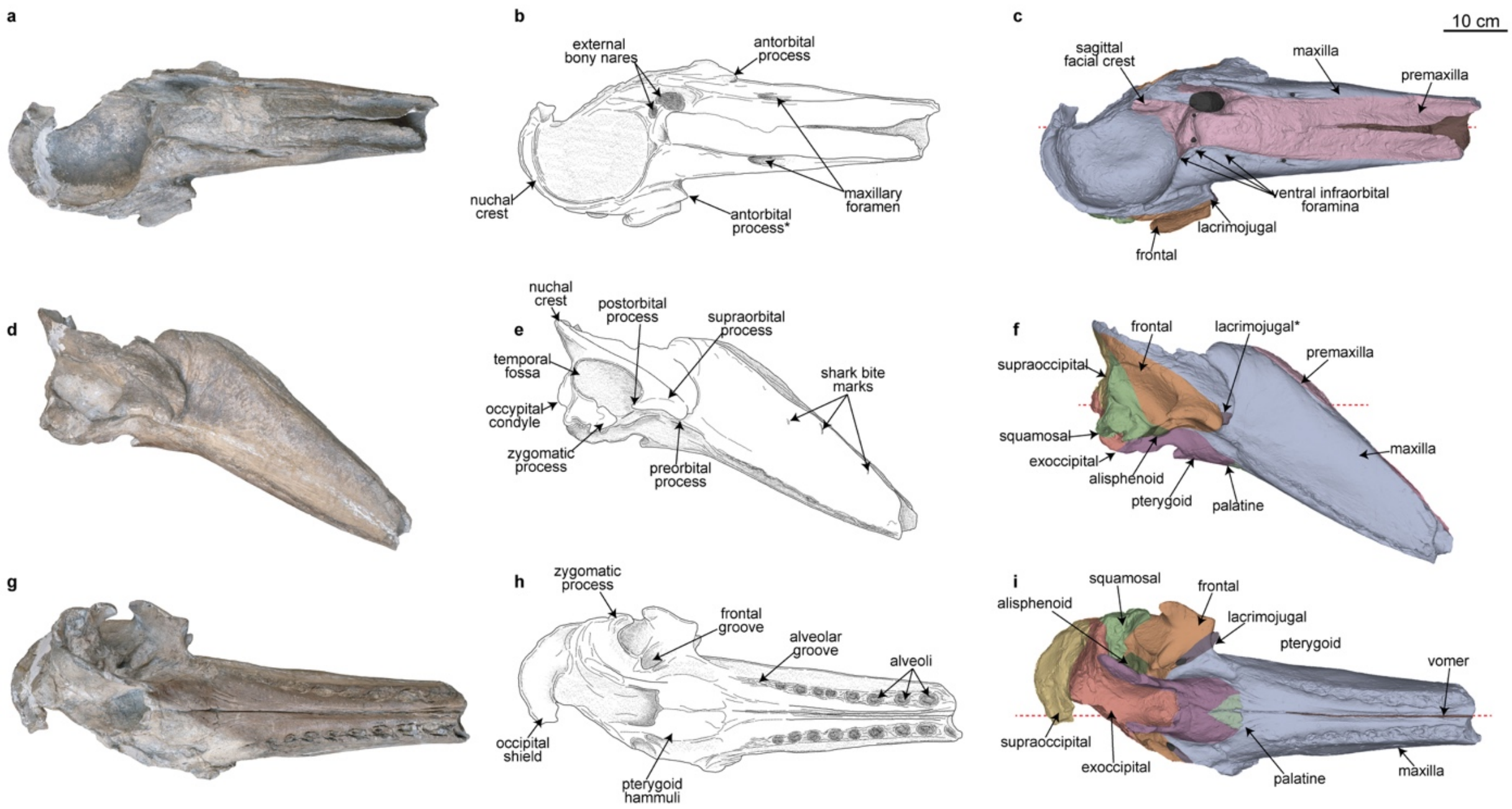

Figure 1. Holotype skull of *Scaphokogia huachafo* sp nov. MUSM 972 in dorsal (a-c), right lateral (d-f) and ventral (g-i) views. Dotted red lines indicate the main cranial axis.

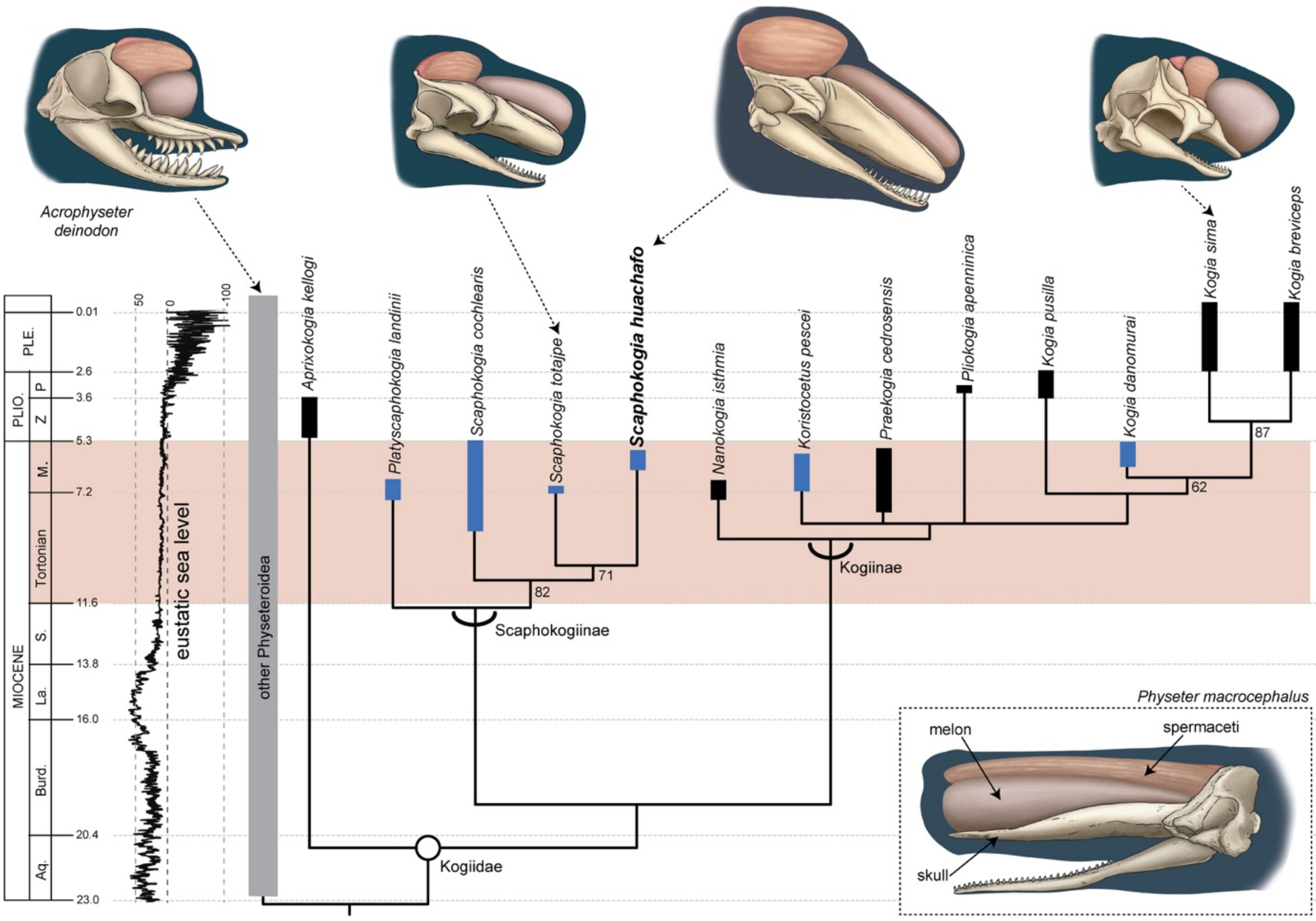

460
461 **Figure 2.** Phylogenetic relationships of *Scaphokogia huachafo* sp nov. within Kogiidae. Time-
462 calibrated phylogenetic tree obtained from 315 most parsimonious trees with a length of 257 steps, a
463 CI of 0.397 and a RI of 0.694.

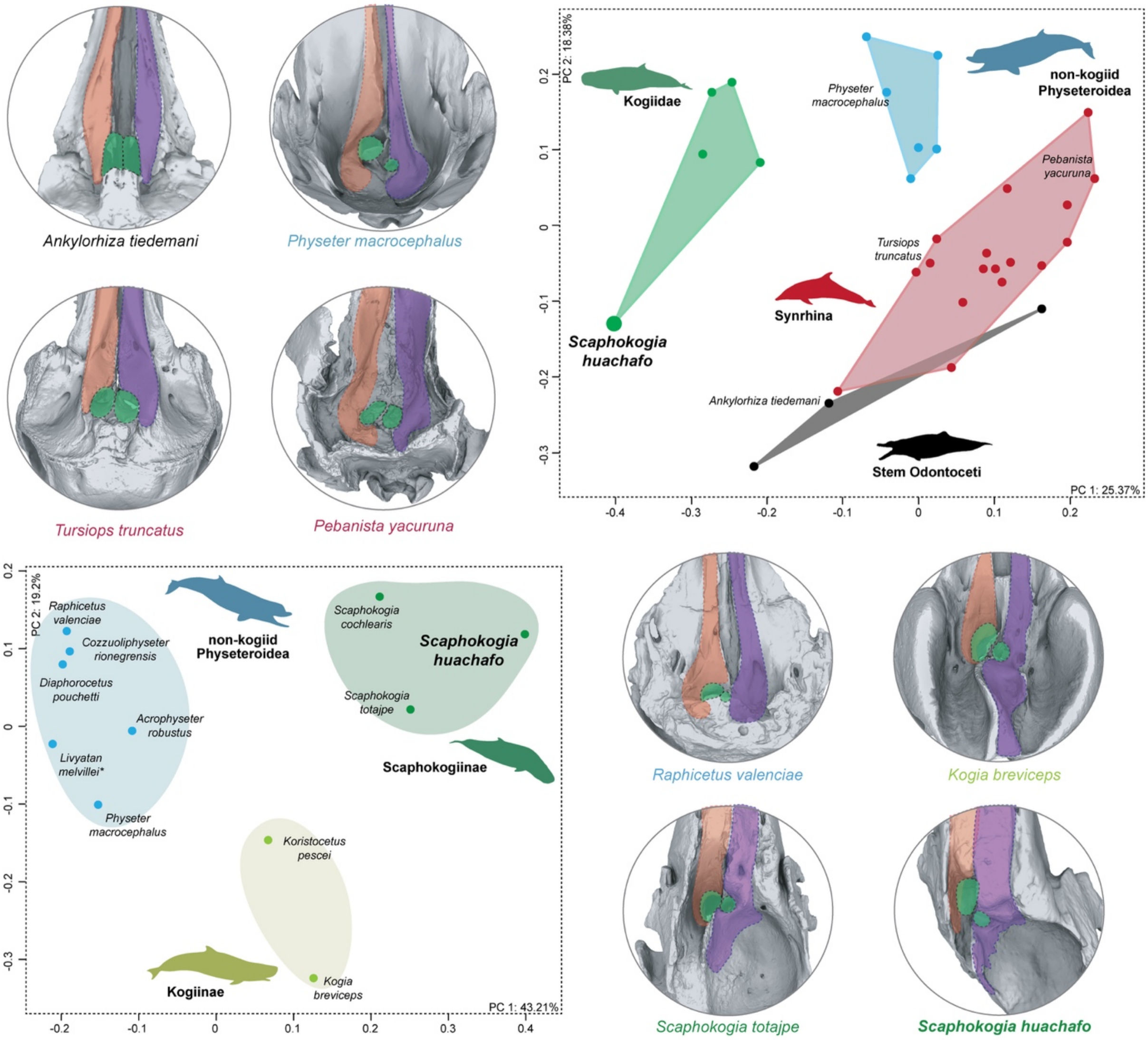

Figure 3. Craniofacial Asymmetry (CFA) analyses of Odontoceti (top) and a subset including only Physeteroidea (bottom). Skulls display the differences between the left (orange) and right (purple) premaxillae and the size of the external bony nares.

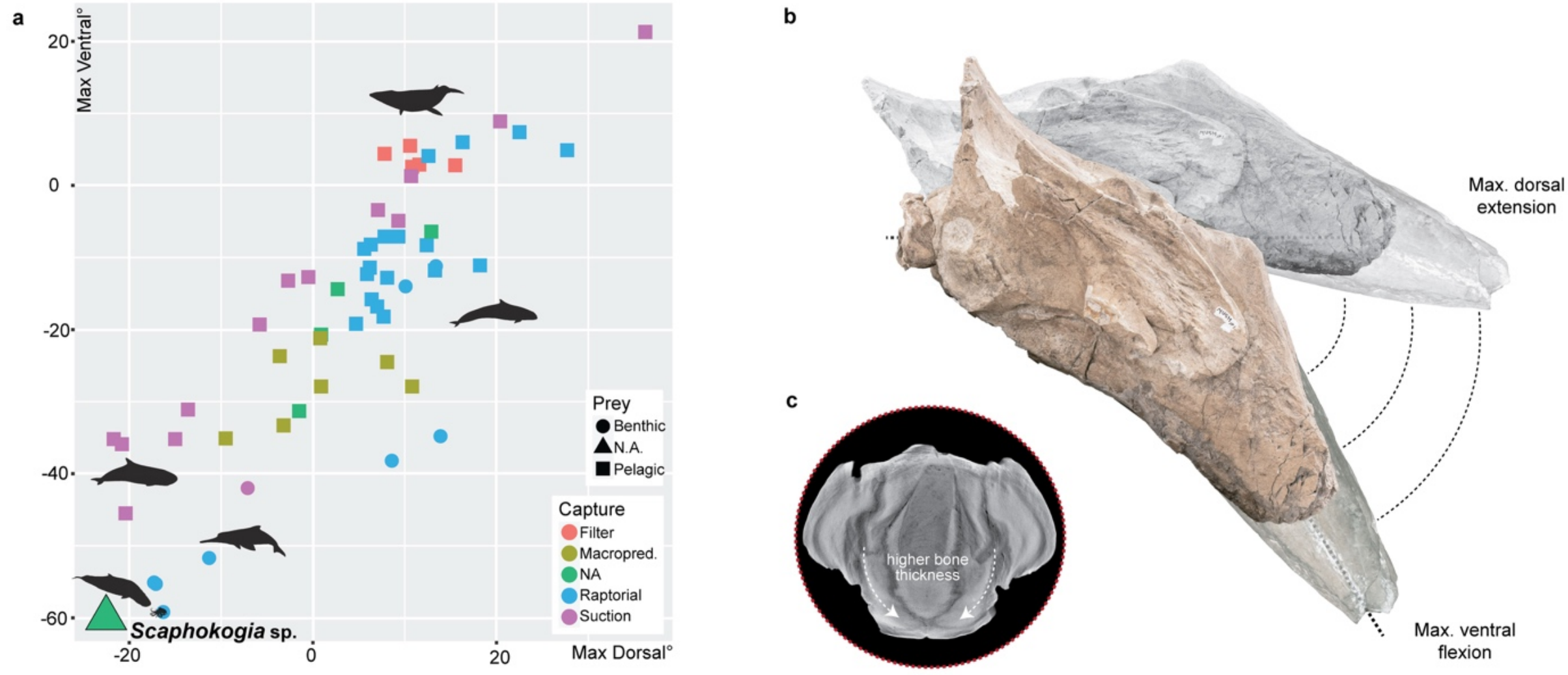

Figure 4. Benthic adaptations in *Scaphokogia* spp: Range of Motion (ROM) of the atlantooccipital joint in Cetacea, including *Scaphokogia* sp. MUSM 171 (a); shapes indicate the general morphology of the clusters recovered. Hypothesised reconstruction of the maximum dorsal extension and ventral flexion of the atlanto-occipital joint in *Scaphokogia huachafo* based in *Scaphokogia* sp. MUSM 171 (b). Virtual cross section of Scaphokogia totajpe indicating the ventral increase in bone thickness (c).
