# Supplementary material for: Extreme cranial asymmetry in extinct benthic feeding sperm whales
Source: Commun Biol. 2026 Nov 9;9:1406. doi: 10.1038/s42003-026-11074-2 (PMC13748650; doi:10.1038/s42003-026-11074-2)
Supplement: Supplementary file 2 [file 42003_2026_11074_MOESM2_ESM.pdf]

Supplementary Information

EXTREME CRANIAL ASYMMETRY IN EXTINCT BENTHIC FEEDING SPERM WHALES

Aldo Benites-Palomino,[1,2,3*] Marcelo R. Sánchez-Villagra[2], Florencia Paolucci[4], Jorge Vélez-Juarbe[5], Alberto Collareta[6], Apolline Alfsen[7], Alton Dooley[8], Dan Omura[9], Mario Urbina[3], Rodolfo Salas-Gismondi[3,10] & Gabriel Aguirre-Fernández[2]

[1]GeoZentrum Nordbayern, Friedrich-Alexander-Universität Erlangen-Nürnberg, 91054 Erlangen, Germany
[2]Department of Paleontology, University of Zurich, Karl-Schmid-Strasse 4 Zürich 8006, Switzerland.
[3]Departamento de Paleontología de Vertebrados, Museo de Historia Natural-Universidad Nacional Mayor de San Marcos, Avenida Arenales 1256, Lima 11, Peru.
[4]División Paleontología Vertebrados, Unidades de Investigación Anexo Museo, Facultad de Ciencias Naturales y Museo, UNLP, CONICET, Av. 60 y 122, B1900AVW La Plata, Argentina
[5]Department of Mammalogy, Natural History Museum of Los Angeles County, 900 Exposition Blvd., Los Angeles, California 90007, U.S.A.,
[6]Dipartimento di Scienze della Terra, Università di Pisa, via Santa Maria 53, 56126, Pisa, Italy;
[7]Department of Evolutionary Morphology, Museum für Naturkunde, Leibniz-Institut für Evolutions- und Biodiversitätsforschung, Berlin, Germany
[8]Western Science Center, 2345 Searl Parkway, Hemet, CA 92543, U.S.A.,
[9]Centro de Desarrollo para la Educación Lima Nikko. Asociación Peruano Japonesa. Av. Gregorio Escobedo 803, Residencial San Felipe, Jesús María; Lima, Perú
[10]Facultad de Ciencias y Filosofía/ Laboratorios de Investigación y Desarrollo, Universidad Peruana Cayetano Heredia, Lima, Peru
*Corresponding author: aldo.benites@fau.de

Table of Contents:

- Supplementary Figures 1-10
- Supplementary Notes 1: Geological and environmental context
- Supplementary Notes 2: Extended description of *Scaphokogia huachafo* gen. et. sp. nov.
- Supplementary Notes 3: Phylogenetic definitions
- Supplementary Notes 4: List of Morphological Characters
- Supplementary Table 1: Measurements of *Scaphokogia huachafo* MUSM 972
- Supplementary Table 2: List of Taxa Featured in the morphometric analyses
- Supplementary References

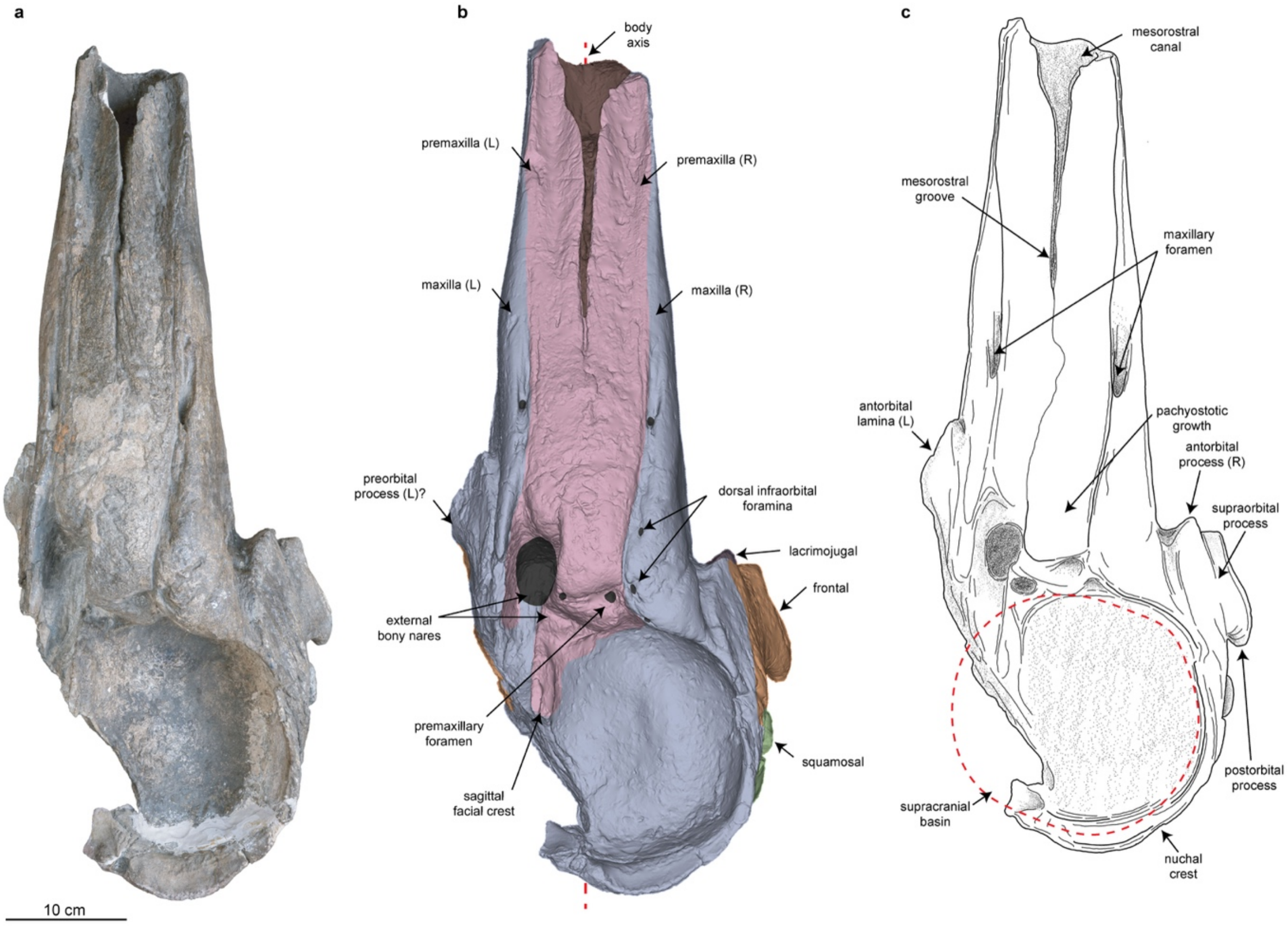

Supplementary Figure 1. Holotype skull of *Scaphokogia huachafo* sp nov. MUSM 972 in dorsal view: photograph of the specimen (a), 3D model highlighting the bone structures (b) and schematic drawing (c).

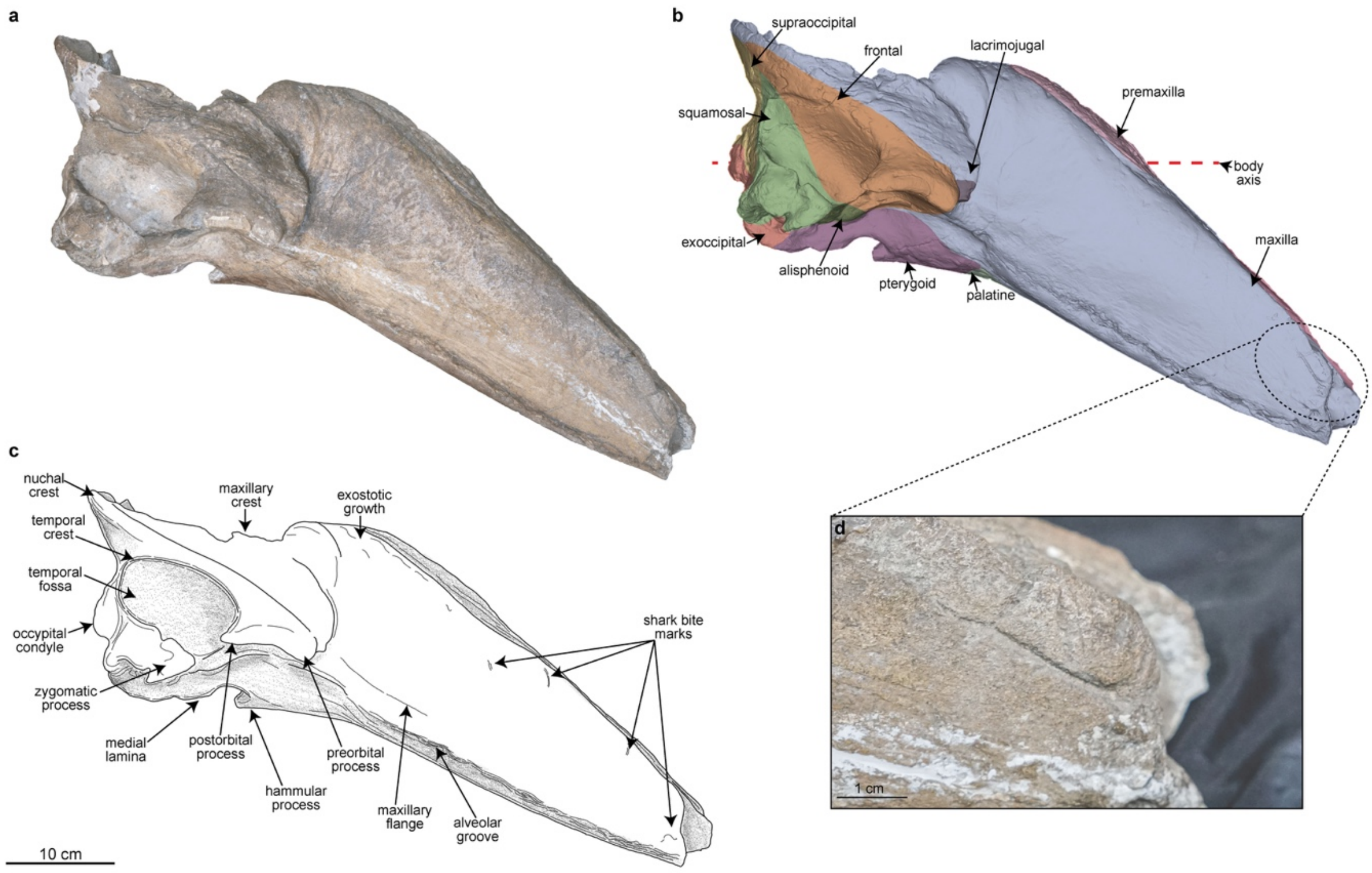

Supplementary Figure 2. Holotype skull of *Scaphokogia huachafo* sp nov. MUSM 972 in right lateral view: photograph of the specimen (a), 3D model highlighting the bone structures (b), schematic drawing (c) and close up of the shark bite marks (d).

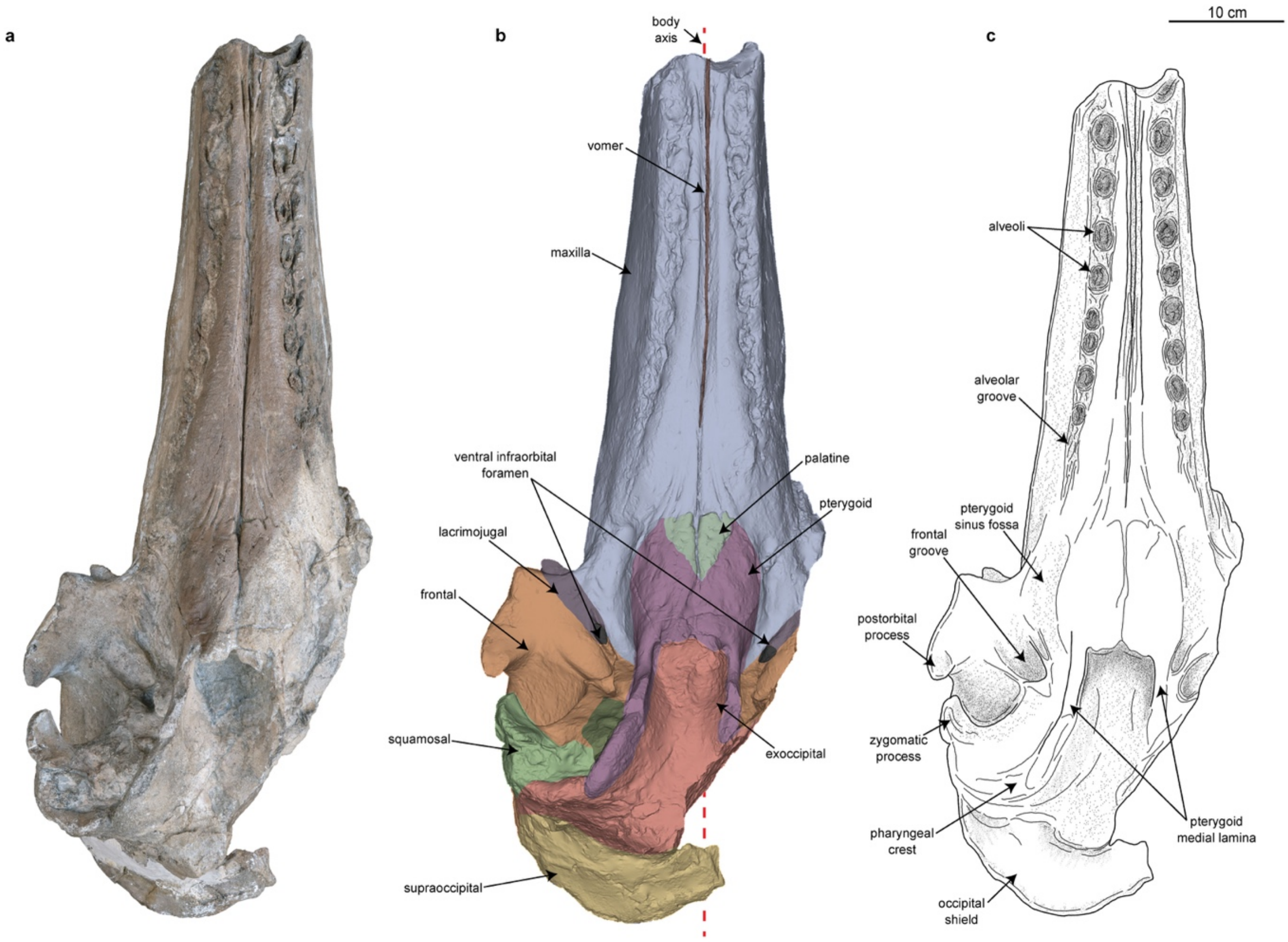

Supplementary Figure 3. Holotype skull of *Scaphokogia huachafo* sp nov. MUSM 972 in ventral view: photograph of the specimen (a), 3D model highlighting the bone structures (b) and schematic drawing (c).

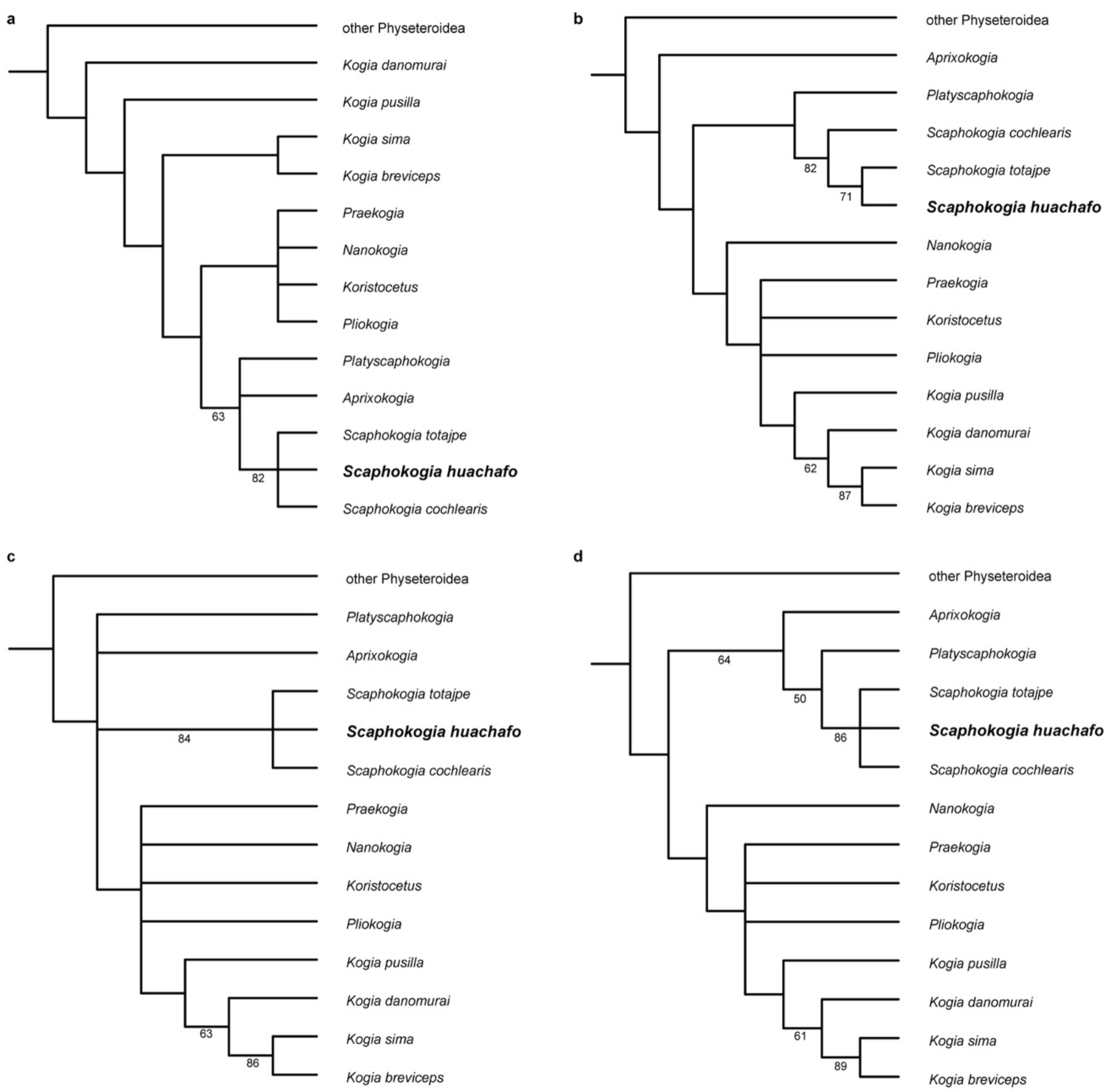

Supplementary Figure 4. Phylogenetic relationships of *Scaphokogia huachafo* sp. nov. with other

Kogiids. Parsimony analyses used unordered (a, b) vs. ordered (c, d), and unweighted (a, c) vs. implied-weighted (b, d) characters. Numbers under branches indicate bootstrap support values.

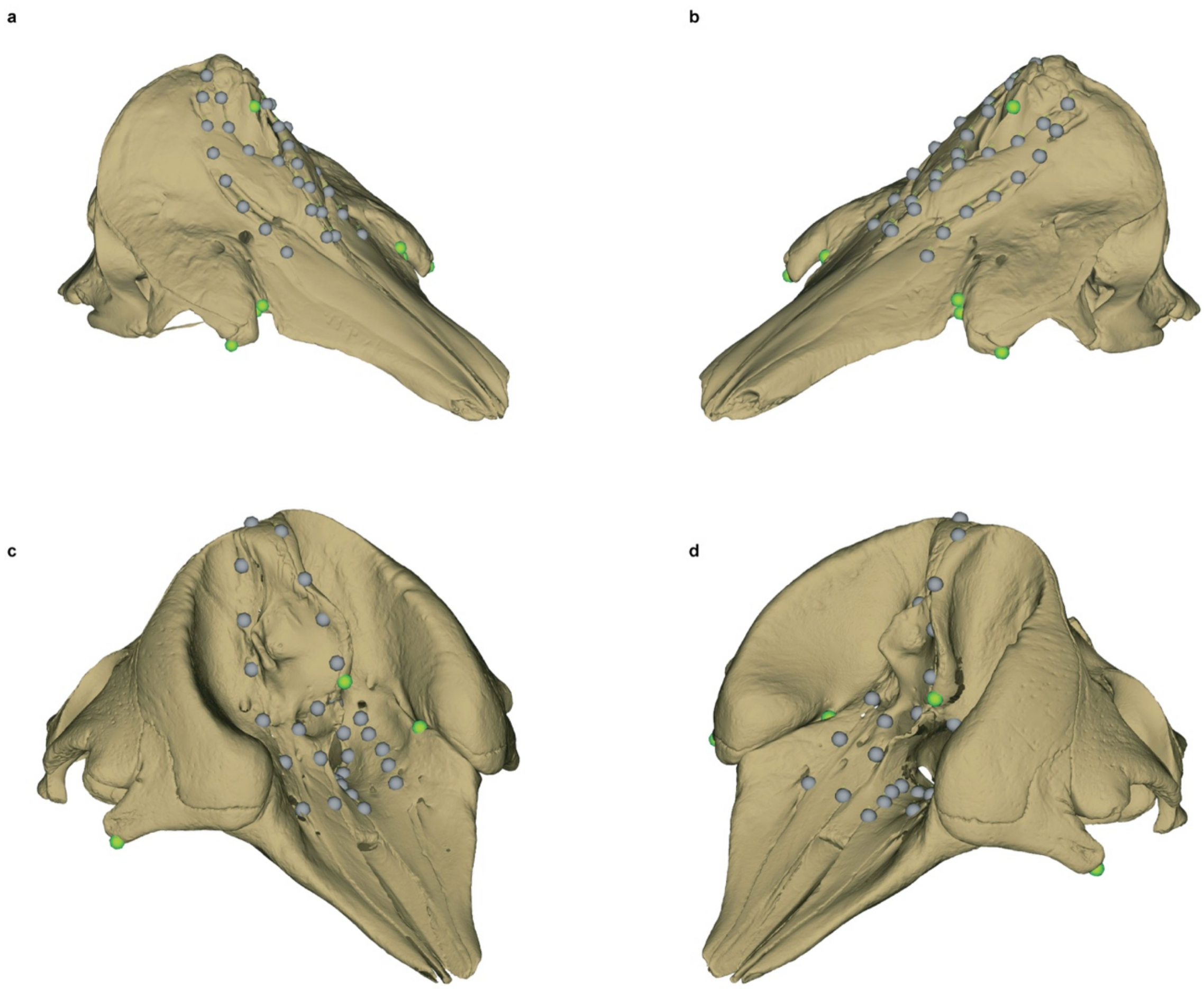

Supplementary Figure 5. Skulls of *Monodon* and *Kogia* indicating the Type-I anatomical landmarks (in green) and the reference point for the (sliding) semilandmarks (in grey) taken across 31 cetacean skulls for our analyses of craniofacial bones.

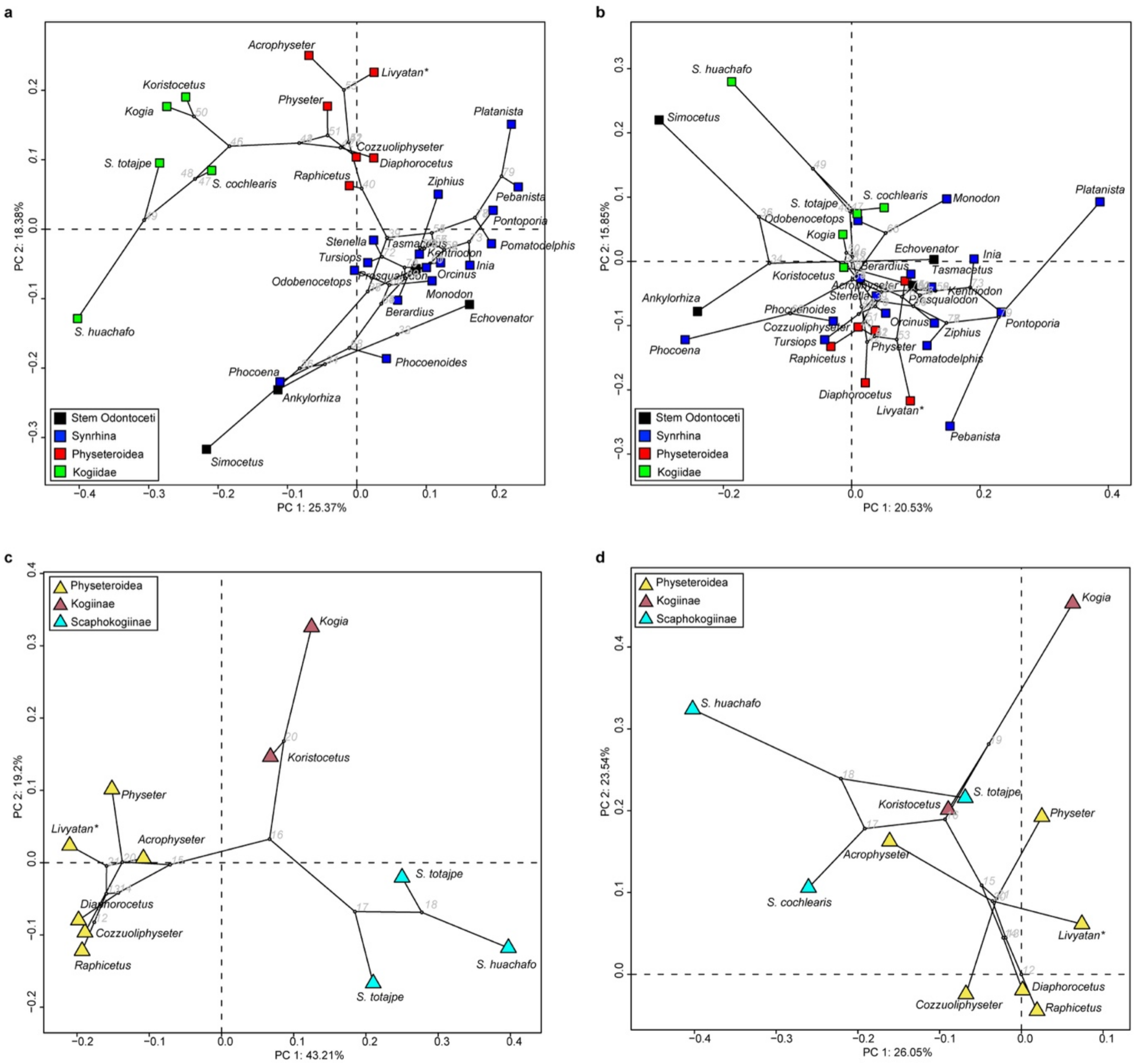

Supplementary Figure 6. Arrangement of craniofacial bones analyses of 31 cetacean skulls including the phylomorphospace of the entire dataset (a), a subset of only physeteroids (b), the phyloPCA of the entire sample (c) and a subset of solely physeteroids (d).

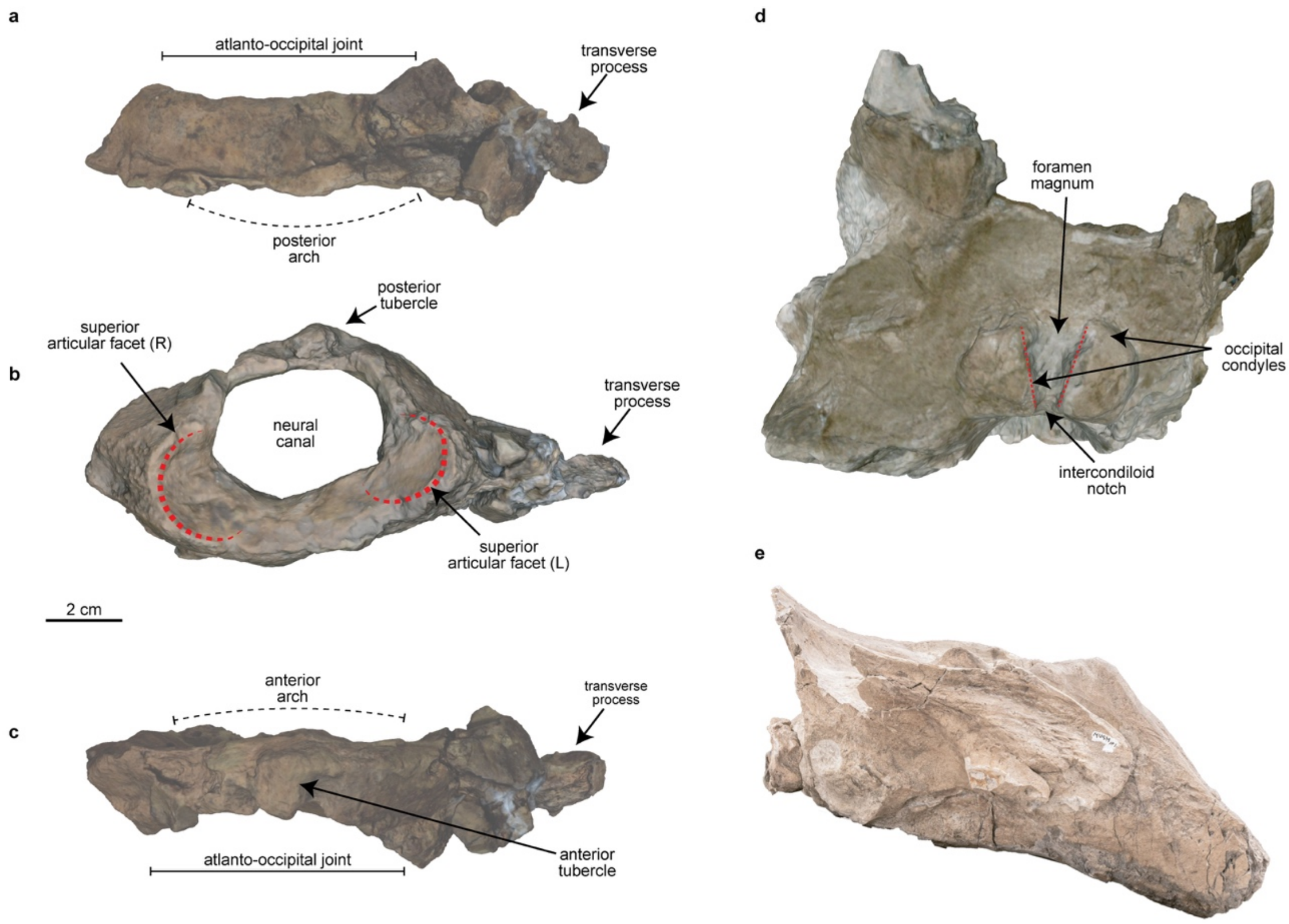

Supplementary Figure 7. *Scaphokogia* sp. MUSM 171 highlighting the atlas in dorsal (a), anterior (b) and ventral (c) views; and skull in posterior (d) and right lateral views (e). Note the asymmetric distribution of the articular facets and the occipital condyles.

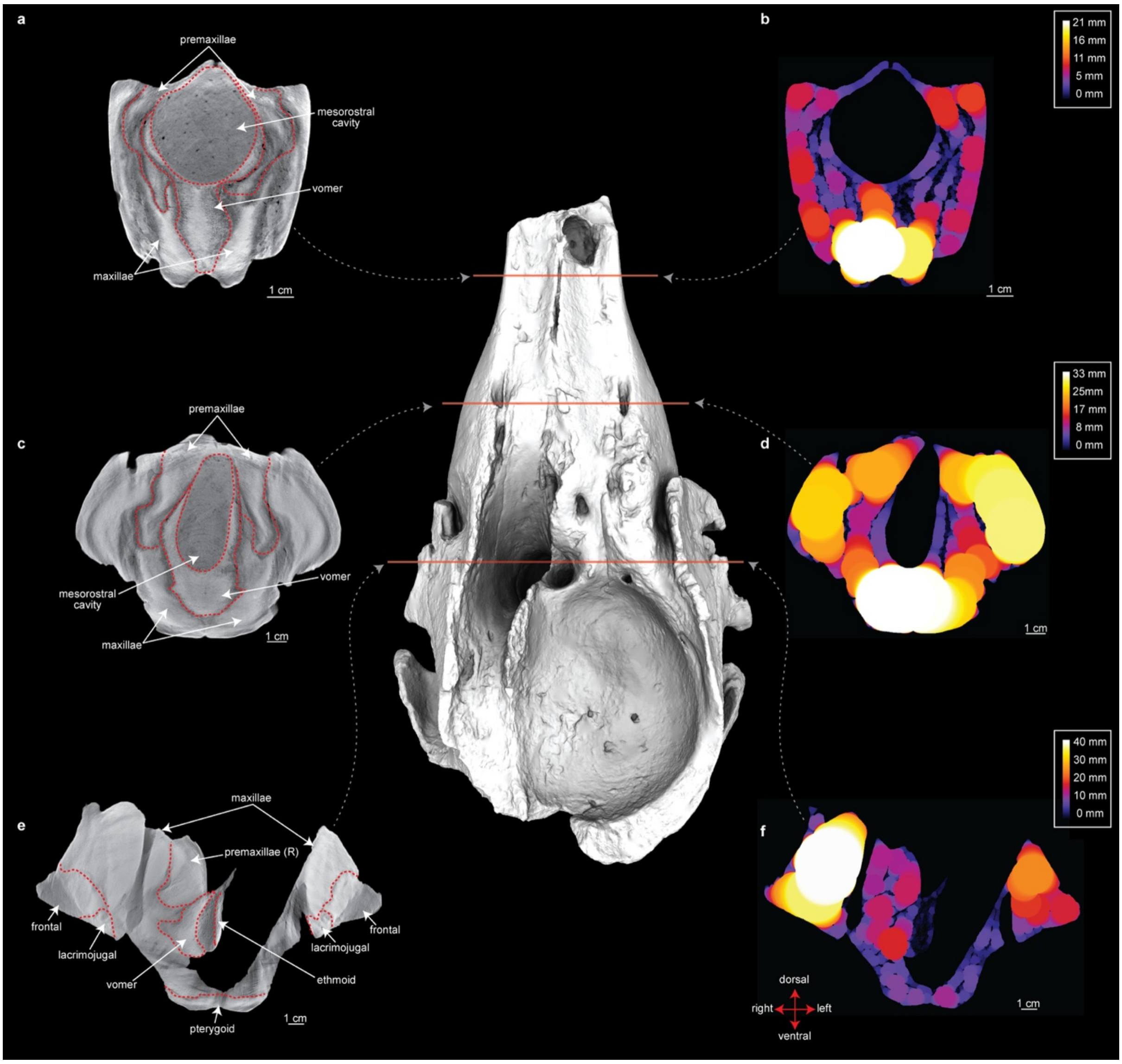

Supplementary Figure 8. Local bone density across the skull of *Scaphokogia totajpe* MUSM 973, taken at the tip of the rostrum (a, b) midlenght of the rostrum (c, d) and the rostral base (e, f). Note the higher bone density ventral area of the rostrum and right lateral maxillary crest.

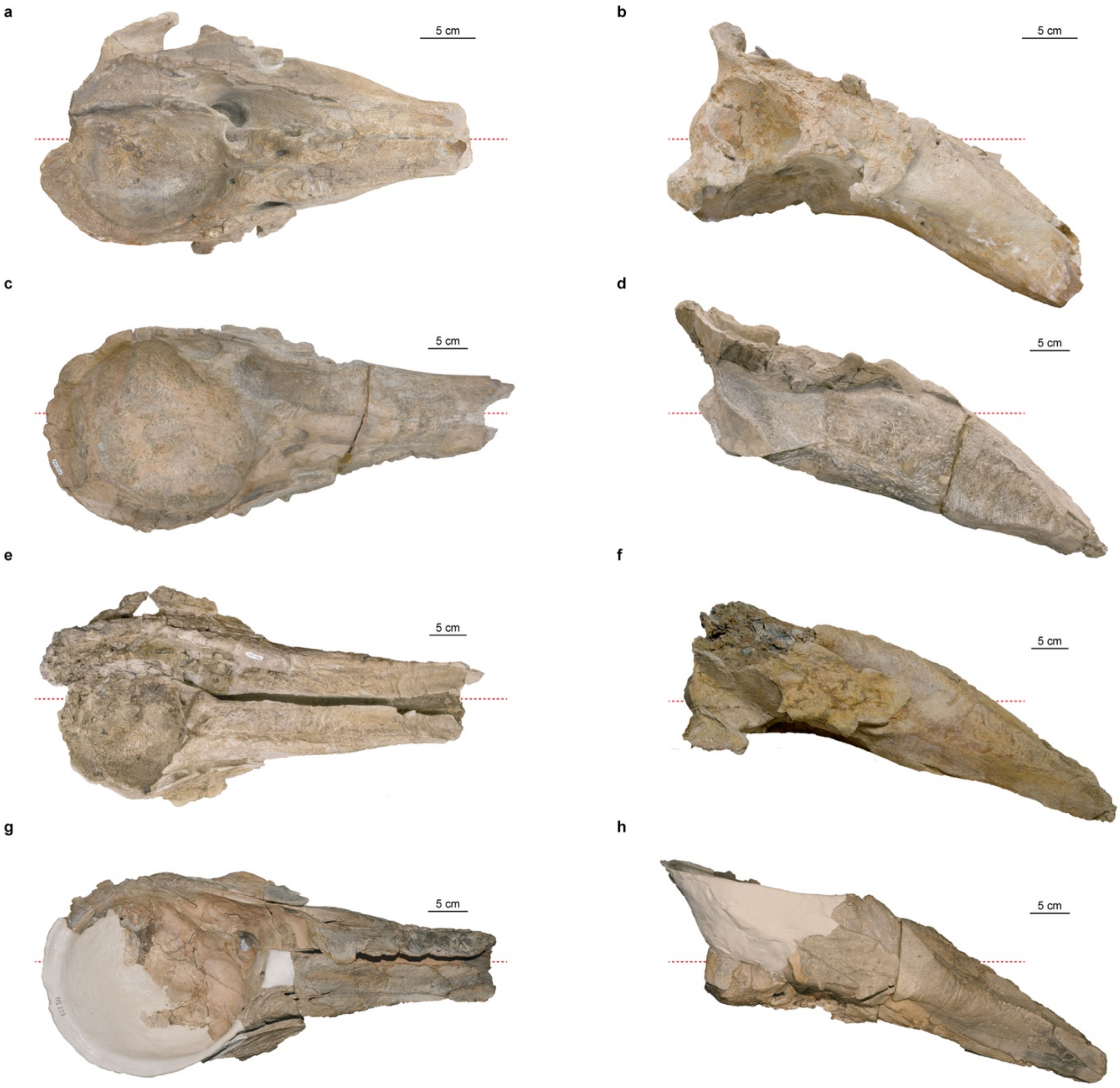

Supplementary Figure 9. Gradient of rostral deviation across the genus *Scaphokogia*: *Scaphokogia totajpe* MUSM 973, *Scaphokogia cochlearis* MUSM 1998, *Scaphokogia* sp. MUSM 3244 and *Scaphokogia cochlearis* MHN PPI-229.

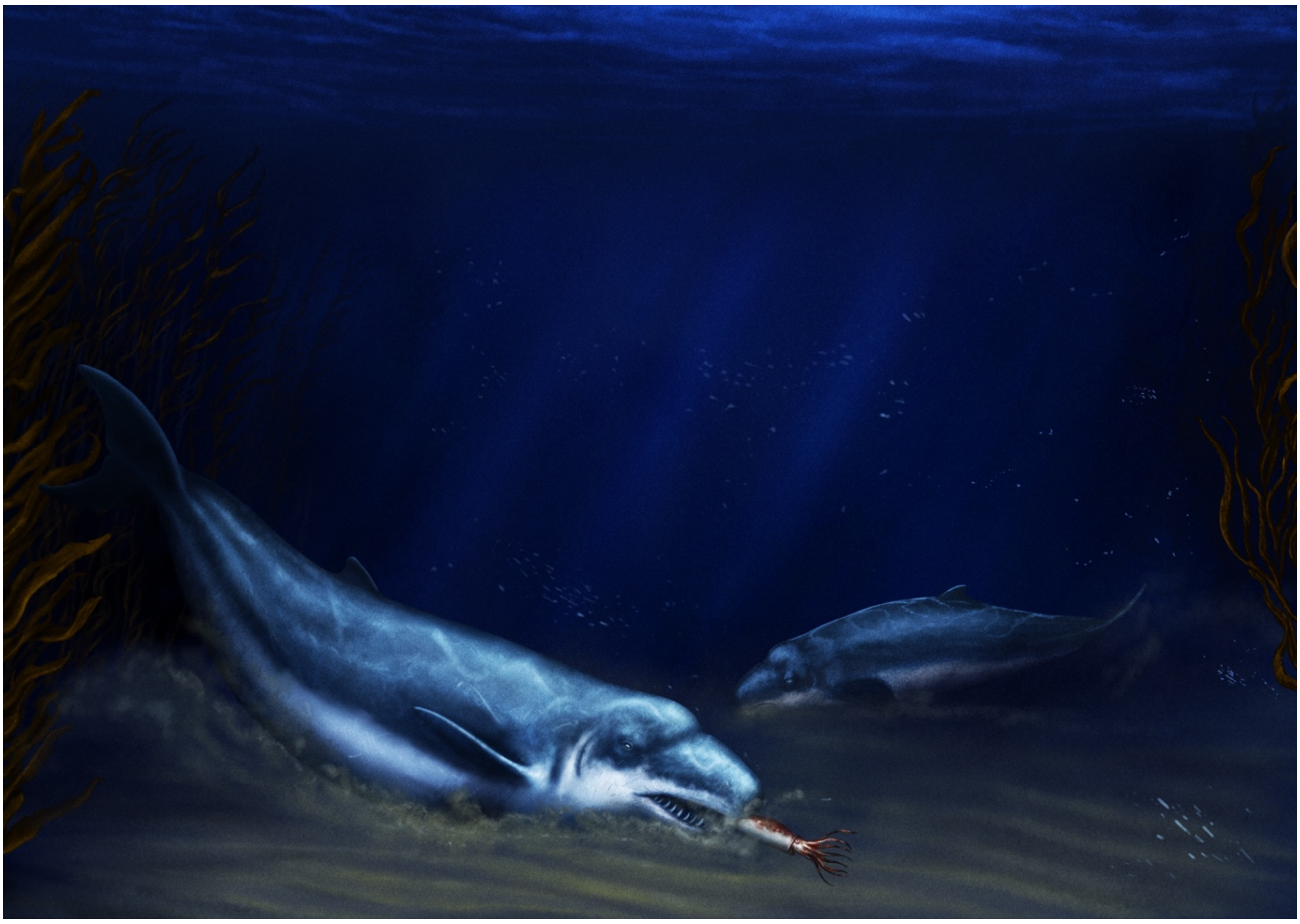

Supplementary Figure 10. Artistic reconstruction by Jaime Bran of *Scaphokogia huachafo* feeding near the bottom in the shallow coastal waters during the Late Miocene in Peru

- **Supplementary Notes 1: Geological and environmental context**

The Pisco Formation is an upper Neogene shallow-marine sedimentary unit that extends along the southern Peruvian coastal desert in the regions of Ica and Arequipa. It comprises three significant transgressive-regressive cycles that occurred from the middle Miocene to the early Pliocene [1–4], both in the Eastern Pisco Basin and more recently in the Sacaco Basin[3,5,6]. Renowned as the most important marine Miocene Lagerstätte, the Pisco Formation possesses a wealth of well-preserved fossils, most notably fully articulated whale skeletons with baleen *in situ* [7–9]. The Sacaco sub-basin, located in the northern Arequipa region (Fig. 1A), represents the southernmost extent of the Pisco Formation and consists of a series of marine transgressions deposited between 9.5 and 4.5 Ma (Muizon and DeVries, 1985; Ehret et al., 2012). During this period, the Sacaco area was covered by a shallow sea adjacent to a rugged coastline, creating protected bay-like environments, peninsulas, archipelagos, and marine lagoons (Marocco and Muizon, 1988). The holotype specimen of *Scaphokogia huachafo* MUSM 972 was collected in the higher layers of Aguada de Lomas, Sacaco basin (Arequipa, Peru). The fossil-bearing horizon was deposited between 6.3-5.6 Ma., during a shallow marine transgression.

- **Supplementary Notes 2: Extended description of *Scaphokogia huachafo* gen. et. sp. nov.**

General description of the skull—The holotype skull of *Scaphokogia huachafo* gen. et sp. nov. (MUSM 972) is mostly complete, lacking only the squamosal and the lateralmost portion of the frontal on the left side of the cranium. The specimen corresponds to a fully grown adult, as indicated by the well-ossified cranial elements and fully closed or ankylosed cranial sutures. The skull has a condylobasal length of 632 mm and a maximum anteroposterior (transversal longitudinal) length of 701 mm, measured from the posterior tip of the nuchal crest to the anterior tip of the rostrum. The rostrum is massive and tube-like, formed by the premaxillae, maxillae, and vomer, with partial ossification of the mesethmoid. It is ventrally oriented, forming an angle of 31° with the main cranial axis. Ventrally, eight well-developed maxillary alveoli are present, continuing posteriorly into a deep alveolar groove. In the facial region, the external bony nares are displaced toward the left side of the skull, with the left naris being substantially larger than the right. Cranial asymmetry is also evident in the orbital region, where the left antorbital process and antorbital notch are positioned more anteriorly than their right counterparts, suggesting an anterior displacement of the left orbital region. Posteriorly, the supracranial basin is proportionally smaller but deeper than in other scaphokogiines, with a mostly circular outline and restriction to the facial region of the skull. The asymmetric deepening of the supracranial basin extends into the intracranial cavity, which is dorsoventrally compressed on its right side. The temporal fossa is longer anteroposteriorly than high, resembling the condition observed in other members of *Scaphokogia*. The nuchal crest markedly overhangs the occipital condyles, which are proportionally small. The orientation of the occipital condyles suggests that the skull was directed downward in life, consistent with a benthic lifestyle.

Premaxilla—The dorsal surface of the premaxilla is concave from the level of the right maxillary foramen till its anterior end, such condition creates two sunk spaces that run parallel along the porcelaneous region of the premaxilla, being laterally delimited by the premaxilla-maxilla suture and medially by the mesorostral groove. The mesorostral groove is only open on the anterior half of the rostrum, closing abruptly at about mid-length. Due to dorsal pachyostotic growth, the suture between both premaxillae is not easily distinguishable until the anterior wall of the left bony naris. Unlike the small and localised bony excrescences of *Scaphokogia cochlearis* or *S. totajpe* that are mostly restricted to the right premaxilla and maxilla, S. huachafo has a pachyostotic growth that extends to the left premaxilla, covering most of its dorsal surface in the region. Furthermore, this swollen part of the bone makes the dorsal surface of the premaxillae convex posteriorly till the anterior wall of the supracranial basin. Both premaxillae extend mostly parallel on the rostral surface, being roughly the same size. From the level of the left antorbital process, the right premaxilla expands medially towards the left side of the skull, reducing the transversal width of the left premaxilla and its dorsal exposition, overlapping it. This condition is enhanced by the widening of the right maxilla slightly anterior to the right antorbital notch, similar to the condition observed in *S. cochlearis*[11]. Slightly anterior to the level

of the right antorbital notch the left premaxilla becomes nearly vertical, dropping abruptly at an 80° angle creating a robust and bulky anterior wall of the left bony naris. The left premaxilla also creates the lateral wall of the left naris roughly to a point in line with the right premaxillary foramen. The right premaxilla reaches its maximum height at the level of the right antorbital notch, before dropping abruptly to nearly an 85° angle to form the anterior wall of the supracranial basin. The right premaxillary foramen, which displays a semi-elliptical profile, is located within this wall slightly below the rostral surface and near the suture with the maxilla. The right premaxillary foramen reaches an anteroposterior length of 4 mm and approx. a 12 mm width. The right premaxilla extends leftwards posteriorly, to form the walls of the reduced right bony naris, which is roughly located at mid length between the rostral surface and the supracranial basin floor. In *Scaphokogia* the right bony nares is posterodorsally oriented, contrasting with the condition observed in other physeteroids where the right naris is mostly dorsally oriented. Such disposition of the bony nares suggests a posterodorsal orientation of the right nasal passage, a condition that might be related to the greatly excavated supracranial basin. The posteriormost part of the right premaxilla extends into the supracranial basin displaying a triangle-like shape in dorsal view, and also contributing as a tiny peg to the medial surface of the sagittal facial crest as observed in other members of the genus *Scaphokogia*.

Maxilla– The maxillae are exceptionally enlarged in *Scaphokogia huachafo*. In dorsal view and across the rostrum, the maxilla is exposed laterally to the premaxilla where it runs mostly parallel until the level of the left antorbital notch, where the left maxilla narrows and the right broadens, displacing leftwards the dorsal exposure of the premaxillae. Along the rostrum the maxilla has a moderately concave surface on its lateral and ventral exposures. Only the maxillary flange, which starts roughly at the level that the alveolar groove ends, creates a small protrusion laterally. Three large shark bite-marks can be identified on the right side of the rostrum, reaching a length between 32-51mm[12]. Ventrally, eight large alveoli can be distinguished, which display a sub-circular outline. The walls of the alveoli have an uneven surface due to some irregular growth of the bone, or exostosis which partly resembles the condition previously described in *Acrophyseter*[13], due to the position of the bony outgrowth. However, this growth might be related to some intentional damage to the gum, as the exostosis in some areas has grown into the alveoli. Due to this uneven surface, no additional alveoli can be distinguished. Furthermore, the condition observed in *S. huachafo* is a particular one, as besides the alveoli each maxilla display a moderately deep alveolar groove, with rather rough lateral and medial margins. There are no teeth preserved in the holotype specimen; however, some dentine remains can still be distinguished in the anteriormost alveoli, identified as part of a tooth reabsorption process. Posteriorly, the alveolar groove extends until the level of the left antorbital lamina. Medial to the alveoli, the vomer is partly exposed on the anterior half of the rostrum as a tiny strip. The surrounding portion of the maxilla between the alveoli and the vomer has a bulbous profile, with several canals. The left antorbital process is better preserved than the right one, being transversely compressed forming a lamina-like structure. This lamina extends dorsally until the level of the rostrum and extends posteriorly parallel to the anterior wall of the supracranial basin until the level of the postorbital process. The left orbital region appears to be more anteriorly located than the right one, as

suggested by the broken left preorbital process. Such distortion could be the result from the taphonomic process of the skull; however, no evidence of such deformation has been found on other parts or bones of the skull. Furthermore, when considering the eye socket orientation and the long axis of the skull, the right one appears to be more anteriorly oriented and the left one. Before dropping to form the anterior wall of the supracranial basin and at the level of the external bony nares, the right maxilla reaches its maximum rostral width. The surface of the maxilla in this region is rough, mostly due to the bone excrescences which create an ideal insertion site for the greatly enlarged facial muscles as in *Scaphokogia totajpe*[10]. Nevertheless, three dorsal infraorbital foramina can be recognised, following a similar distribution pattern as other Scaphokogiinae, and possessing a mostly rounded profile. The supracranial basin in *Scaphokogia huachafo* is proportionally smaller than that of other Scaphokogiinae, mostly due to the greatly enlarged rostrum. In dorsal view it has a semi-circular outline reaching a diameter of 237mm and 91mm of maximum depth. The basin reaches its maximum depth on the right half of the skull, at the level of the anterior tip of the zygomatic process. It is not clear whether if this deepening of the basin extended into the braincase, but in extant *Kogia*, the skull asymmetry extends into the brain[14]. Only the right lateral wall of the basin is preserved, which continuous posteriorly to form the nuchal crest. The maximum extension of the basin is reached on the right half of the skull, being slightly offset of the main body axis. In this region the dorsal surface of the nuchal crest is mostly flat but anterodorsally wide, creating large insertion areas for the posterior facial muscles. On the left half of the skull, only a small portion of the sagittal facial crest is preserved, making impossible to assess whether if this extended completely into the nuchal crest or deviated joining the lateral left wall of the basin. Nevertheless, a small portion of the left maxillary fossa can be recognised being much shallower than the rest of the basin.

Frontal– In dorsal view, the frontals are mostly covered by the maxillae, except for the right orbit orbital region. Despite being broken, a small tip of the left preorbital process might have preserved near along with the left antorbital process. In lateral view, the suture between the maxilla-frontal runs almost parallel to the long axis of the rostrum, forming an angle of around 30° with the main body axis and its easily recognisable along the supraorbital process, as it is located in a shallow recess. This makes the frontal orientation in *Scaphokogia huachafo* much vertical than the condition observed in other members of the genus *Scaphokogia*. The right orbit is well-preserved, reaching a length of 88mm between the preorbital and postorbital processes. Unlike the extant *Kogia* which possess a postorbital process posteroventrally projected, *Scaphokogia* has a short projection in the same direction. The preorbital process is much dorsoventrally thick with a fairly round anterior tip; however, it is not clear whether this is the *in vivo* condition of a result of the taphonomic process. The supraorbital process of the frontal is bulbous and overhangs laterally, being much robust anteriorly. The entire orbital region of *Scaphokogia* is located much below the level of the rostrum, possessing an orbit much ventral that in *Scaphokogia*. In ventral view, only the right frontal groove is well-preserved, forming an angle of approximately 5 degrees with the main body axis. There are some fragments of the left frontal preserved; however, due to the bad preservation of the left side no further assessments can be done.

Vomer– Unlike other cetaceans, in which the vomer forms a plate like structure on the roof of the mesorostral groove, the vomer in *Scaphokogia* spp. is well-developed and enlarged, a condition unique to this genus. In *S. huachafo* and *S. cochlearis* the most anterior tip of the vomer is exposed as a robust, trapezoidal structure; thus, contrasting the vomer in *S. totajpe* in which the vomer has a 'y' outline. The CT data of *S. totajpe*, shows that the vomer retains this shape all along the rostral length, creating the lateral walls and floor of the mesorostral canal. The vomer is well-ossified, except in its ventral region where it displays a greater density, a pattern also observed in the surrounding portions of the maxilla. In a juvenile specimen referred to *S. cochlearis* (MUSM 955) which has a rostrum broken at mid length, a similar pattern is observed. Due to its large rostral development, *Scaphokogia huachafo* most probably had a similarly developed vomer.

Temporal Region–The temporal fossa of *Scaphokogia* displays a rectangular outline in lateral view, being moderately deep similar to that of *S. cochlearis*, but shallower than the one present in *S. totajpe* or *Platyscaphokogia*. The fossa extends partly into the occipital plane of the skull, reaching a maximum anteroposterior length of 165mm and a maximum height of 81 mm. The temporal crest is more prominent on its posteroventral portion, where it joins the supramastoid crest of the squamosal.

Lacrimojugal– Only the right lacrimojugal is preserved. Both the dorsal and lateral surfaces of the lacrimojugal are broken, as the entire antorbital lamina is missing. In lateral view the lacrimojugal displays a triangular profile, with a short projection between the maxilla and the frontal. Since the most distal portion of the bone is lost it is not possible to discern whether if the anchor site for the masseter was well-developed. Nevertheless it shouldn't have been large, as ventrally the bone is mostly reduced to a narrow strip.

Palatine–Only a minor portion of the palatines is exposed ventrally, as these are mostly covered by the pterygoid. Each palatine displays a triangular-like outline, being particularly bulky due to the pachyosteosclerosis of the ventral region of the skull. However, and unlike the condition observed in other members of the genus *Scaphokogia*, the palatines do not form a single ankylosed unit but two separate plate-like structures. The palatines have a series of sulci shared with the posteriormost part of the maxilla, which are laterally oriented towards the pterygoid sinus fossa.

Pterygoid–The pterygoid is concave in its ventral surface due to the high pachyosteosclerotic growth of the bone, a condition shared with *Scaphokogia cochlearis* and *S. totajpe.* The pterygoid reaches an anteroposterior length of 217mm, of which 102 correspond to the hamular process. The anterior limit of the pterygoid is difficult to distinguish from the corresponding palatine due to the heavy fusion of the bone, nevertheless a few strips hint to the position of this suture. This anteriormost region of the pterigoids have a pointed outline. In the hamular portion of the pterygoid, the median suture between both pterygoids is closed but not ankylosed and is slightly deflected towards the right side of the skull. The pterygoid hammuli in *Scaphokogia huachafo* has a mostly rounded outline in ventral view, with

the exception of an anterior region where the palatines are exposed. Lateral to the pterygoid hammuli, the pterygoid sinus fossa is long and narrow; however, as in *Scaphokogia totajpe* and in the combination with the bulky profile of the hammuli, the attachment area for the pterygoidal muscles is well-developed. The pterygoid medial lamina forms the lateral walls of the choana, and it continues posteriorly into the pharyngeal crests, where it joins the basioccipital to form the basioccipital crests. Unlike *Scaphokogia cochlearis* the dorsal plate does not project laterally to cover the alisphenoid, but it runs parallel as a narrow strip.

Alisphenoid–The alisphenoid has a trapezoidal outline in ventral view, with little define limits with the surrounding bones due to the heavily ankylosed sutures. The ventral surface of the alisphenoid is concave, a condition present in other Scaphokogiinae and extant *Kogia*. Near the contact with the frontal, the inferior and superior orbital fissure can be recognised, which reach a diameter of roughly 9mm and 8mm respectively. The foramen ovale is located more posteriorly than both fissurae, reaching a diameter of about 6 mm.

Squamosal–Only the right squamosal is preserved, which is mostly complete. In lateral view the zygomatic process is mostly triangular, with a rounded anterior tip. The dorsal portion of the process is mostly concave, with the exception of its outermost portion where the supramastoid crest is located. Ventrally, the zygomatic process becomes thicker on its proximal end, lacking any additional anterior expansion or projection as the condition observed in *Scaphokogia totajpe*. The glenoid fossa is shallow and reaches a maximum length of 28mm, being mostly medially oriented. Posterior to it, the postglenoid process extends roughly until the level of the posttympanic process. The falciform process is difficult to recognise as it has either been eroded away or is greatly reduced, similar to the condition observed in *Scaphokogia totajpe*. The region of the anterior meatus is not well-preserved, impeding to recognise most of the structure; nevertheless, the anterior wall is thickened and posterolaterally oriented as in *Scaphokogia totajpe* or *S. cochlearis*. The posttympanic process is well-developed as in other kogiids to house the enlarged posterior process of the tympanic.

Occipital Region–Since part of the left occipital condyle is lost, the skull was oriented taking in account the right condyle and part of the surrounding occipital surface. The occipital shield can be divided in two regions: the bottom half, which surround the condyle and extends until the level of the temporal fossa, is anterodorsally oriented; and the second and most dorsal portion, which corresponds to the occipital portion of the nuchal crest, which is posteroventrally oriented, overhanging the occipital condyles. Most of the outer surface of the occipital shield is convex, with the exception of a depression lateral to the condyle, interpreted dorsal condyloid fossa, which serves as the attachment site for the anterior and lateral *rectus capitis* muscle. The condyle is small, reaching a height of 67mm and a width of 51mm. The preserved portion of the foramen magnum suggests that it might have had a circular outline, being higher than wider. The paraoccipital process is much ventrally extended that the posttympanic process, but not surpassing posteriorly the condyles. The jugular notch is easily recognisable reaching a 7mm width and a depth of 9.5mm

- **Supplementary Notes 3: Phylogenetic definitions**

a) Kogiidae are diagnosed by the following combination of characters: rostrum with a bottle-like constriction in dorsal view (c.10{2}); development of a facial sagittal crest as a shelf covered by the right premaxilla (c.13{1}); presence of a compartmentalized supracranial basin divided into left and right maxillary fossae (c.16{1}); three to four small dorsal infraorbital foramina (c.28{0}); level of the preorbital process of the frontal considerably lower than the lateral margin of the rostrum base (c.36{2}); partly developed notch (paraoccipital concavity) in the posterior region of the postglenoid process (c.41{1}); dorsoventrally thickened lateral margin of the supraorbital process of the maxilla forming a subvertical wall (c.44{1}); small teeth (c.53{0}); and absence of enamel (c.54{1}).

b) The clade formed by Kogiinae and Scaphokogiinae, excluding *Aprixokogia*, shares the following features: presence of a supracranial basin (c.12{1}); lack of posterior widening of the right premaxilla (c.29{0}); low temporal fossa occupying less than half the skull height (c.46{1}); upper tooth row transformed into a shallow alveolar groove without septa (c.51{2}); and anteroposterior positioning of the last upper alveolus distinctly anterior to the antorbital notch (c.57{2}).

c) Kogiinae are diagnosed by the following characteristics: right antorbital notch located within the supracranial basin (c.5{1}); posterior end of the sagittal facial crest forming a 'W'-like shape where it joins the nuchal crest (c.14{1}); shallow to moderately developed supracranial basin with little to no effect on the surrounding cranial bones (c.17{0}); supracranial basin in dorsal view with a boxy or squared posterior outline (c.20{0}); hamular process of the pterygoid flat or square-shaped (c.25{0}); postglenoid process of the squamosal extending ventrally longer than the post-tympanic process (c.40{0}); and a deep and wide paraoccipital concavity located posterior to the postglenoid process (c.41{2}).

d) The genus *Kogia* is characterized by the following features: triangle-shaped rostrum in dorsal view (c.10{0}); supracranial basin extending across the entire dorsal surface of the rostrum (c.12{2}); well-developed supracranial basin displacing surrounding cranial bones (c.17{1}); round to semi-circular posterior wall of the supracranial basin (c.20{1}); dorsoventrally thickened supraorbital process protruding as a rounded shelf (c.26{1}); and a flat or concave occipital forming an angle of approximately 90 degrees with the main body axis (c.42{2}).

e) The Scaphokogiinae clade is diagnosed by the following characteristics: moderately long rostrum with a ratio to the bizygomatic width ranging between 0.95 and 1.2 (c.1{1}); anterior wall of the supracranial basin forming an angle of 45 to 90 degrees with the main

body axis (c.18{1}); flat or concave occipital shield forming an angle greater than 90 degrees with the main body axis (c.42{3}); and the presence of a narrow groove on the ventral surface of the squamosal (c.49{1}).

f) The *Scaphokogia* genus is diagnosed by the following characteristics: mesorostral groove completely closed (c.6{2}); tubular rostrum with a height-to-width proportion exceeding 0.8 (c.8{1}); dorsoventrally thickened vomer at the anterior tip of the rostrum (c.9{1}); rostrum in dorsal view exhibiting a rectangular outline due to its mostly parallel lateral margins (c.10{1}); constriction of the premaxilla, evidenced by the premaxilla-maxilla suture being roughly anteriorly directed (c.11{1}); transversely thickened mesethmoid in the nasal septum (c.22{1}); and deep alveoli with rudimentary septa (c.51{1}).

g) *Scaphokogia huachafo* is diagnosed by the following features: three large dorsal infraorbital foramina (c.28{1}); frontomaxillary suture forming an angle greater than 35 degrees (c.37{2}); deep alveoli with well-defined septa (c.51{0}); ratio between tooth width and bizygomatic width exceeding 0.05 (c.53{1}); and the last upper alveolus positioned slightly anterior to the antorbital notch (c.57{1}).

- **<u>Supplementary Notes 4: List of Morphological Characters</u>**

1) Rostrum length:
    0. Rostrum elongated, ratio between rostrum length and skull width higher than 1.2.
    1. Rostral ratio between 1.2 and 0.95.
    2. Short rostrum, ratio below 0.95.

2) Tip of the rostrum formed by maxilla, premaxilla, and vomer:
    0. Formed only by the premaxillae.
    1. Maxilla, premaxilla, and vomer, all forming the tip of the rostrum.

3) Dorsal exposure of maxilla on the rostrum:
    0. Exposure limited to less than half the rostrum length.
    1. Maxilla exposed on more than half the length of the rostrum, narrower than the premaxilla at some levels.
    2. Wider than the premaxilla all along.

4) Antorbital notch (*ordered*):
    0. Absent.
    1. Present.
    2. Transformed into a very narrow slit.

5) Right antorbital notch:
    0. Outside the supracranial basin.
    1. Inside the supracranial basin.

6) Mesorostral groove:
    0. Open.
    1. Partially open at the level of antorbital notch.
    2. Completely closed.

7) Maxillary flange:
    0. Distinctly convex.
    1. Straight.
    2. Concave.

8) Tubular development of the rostrum:
    0. Proportion between the rostrum height and width at midlength lesser than 0.8
    1. Proportion higher than 0.8

9) Dorsoventral thickening of the vomer:
   0. Absent, mostly flat vomer.
   1. Dorsoventrally thickened vomer with a trapezoidal profile in anterior view.

10) Shape of the rostrum in dorsal view:
   0. Triangle-shaped rostrum, with lateral margins converging.
   1. Rectangle-shape rostrum, with mostly parallel margins.
   2. Bottle-like rostrum, parallel margins with a constriction at midlength.

11) Constriction of the premaxilla:
   0. Absent, suture maxilla-premaxilla on the rostrum roughly anteriorly directed.
   1. Present, suture maxilla-premaxilla distinctly anterolaterally directed.

12) Supracranial basin (*ordered*):
   0. Absent.
   1. Present.
   2. Extended onto the whole dorsal surface of the rostrum.

13) Facial sagittal crest (*ordered*):
   0. Absent.
   1. Present as a shelf covered by the pointed right premaxilla.

14) Posterior end of the facial sagittal crest:
   0. Displaced towards the left side of the skull.
   1. Forming a 'w'-like shape when joining the nuchal crest.

15) Transverse widening of supracranial basin:
   0. Ratio between the maximum transverse width of the supracranial basin and the transverse width of the rostrum at base < 1.
   1. Ratio >1.

16) Compartmentalisation of the supracranial basin (*ordered*):
   0. Single basin.
   1. Basin divided in left and right maxillary fossae.
   2. Two lateral maxillary fossae and one premaxillary fossa.

17) Effect of the supracranial basin on its surrounding bones:
   0. Basin shallow or moderately deep not displacing cranial bones.
   1. Heavily developed basin displacing other cranial bones, including the basicranium.

18) Anterior wall of the supracranial basin:
    0. Absent or incipient.
    1. Forming a 45-90° angle with the rest of the skull (main anteroposterior axis of the basin floor).

19) Relative position of the external bony nares:
    0. Anterior to the antorbital notch.
    1. Roughly at the same level of the antorbital notch.
    2. Much posterior than level of the antorbital notch

20) Shape of the posterior wall of the supracranial basin in dorsal view:
    0. Boxy (squared) outline.
    1. Round to semi-circular profile.

21) Asymmetry of bony nares (*ordered*):
    0. Absent or reduced.
    1. Strong, left bony naris significantly larger than right naris.

22) Transversal thickening of the mesethmoid in the nasal septum:
    0. Absent.
    1. Present

23) Right premaxilla:
    0. Posteriorly extended as the left premaxilla.
    1. More posteriorly extended than the left premaxilla.

24) Nasal bones (*ordered*):
    0. Both nasals present.
    1. One nasal absent.
    2. Both nasals absent.

25) Hamular process of the pterygoid:
    0. Flat to-squared shaped.
    1. Bulbous due to the pachyosteoesclerotic growth of the bone

26) Dorsoventral thickening of the supraorbital process:
    0. Absent.
    1. Present, supraorbital process protruding like a rounded shelf.

27) Maximum width of the skull:

0. Less than 40 cm.
1. Between 40 and 60 cm.
2. Between 60 and 100 cm.
3. More than 100 cm.

28) Number and size of dorsal infraorbital foramina (*ordered*):
    0. Small to moderate size foramina, at least three-four.
    1. Three large foramina.
    2. Two large foramina.
    3. One large foramen (maxillary incisure).

29) Widening of right premaxilla:
    0. Not widened posteriorly.
    1. Posterior extreme of the right premaxilla laterally widened, occupying at least one third of the width of the supracranial basin, mostly on the right side.
    2. Right premaxilla more laterally expanded such that supracranial basin overhangs the right orbit.

30) Left premaxillary foramen:
    0. Present.
    1. Reduced or absent

31) Increase in size of the right premaxillary foramen:
    0. Absent, ratio between width of foramen and width of premaxilla at that level below 0.20.
    1. Present, ratio higher than 0.20

32) Anteroposterior level of right premaxillary foramen:
    0. Distinctly anterior to antorbital notch.
    1. Slightly anterior to antorbital notch.
    2. Same level or posterior to antorbital notch

33) Right maxilla reaching the sagittal plane:
    0. Absent
    1. Present

34) Fusion of lacrimal and jugal (*ordered*):
    0. Absent.
    1. Present

35) Projection of the lacrimal-jugal in lateral view:

0. Short or absent.
1. Long

36) Dorsoventral level of the preorbital process of the frontal:
    0. Higher than the lateral margin of rostrum base.
    1. At approximately the same level.
    2. Considerably lower

37) Frontal-maxilla suture:
    0. Forming an angle < 15° with the longitudinal axis of the rostrum.
    1. Between 15° to 35°.
    2. Higher than 35°.

38) Temporal fossa:
    0. Anteroposteriorly longer than distance between antorbital process of the maxilla and anterior wall of temporal fossa(width/height>1)
    1. approximately same length (width/height=1)
    2. distinctly shorter (with/height<1)

39) Zygomatic process of squamosal in lateral view:
    0. ‘L’-shaped with dorsal margin ventrally bending in its posterior portion.
    1. Equilateral triangle shaped, with dorsal margin dorsally bending in its posterior portion (length no more than two times higher than height at mid-length).
    2. isosceles triangle shaped, with dorsal margin dorsally bending in its posterior portion (elongated, length more than two times higher than height at mid-length).

40) Postglenoid process of the squamosal:
    0. Significantly ventrally longer than post-tympanic process.
    1. Roughly same ventral extent as post-tympanic process.

41) Wide notch posterior of the postglenoid process:
    0. Absent.
    1. Present but partially developed, paraoccipital concavity moderately excavated.
    2. Present and well developed, paraoccipital concavity transformed in a wide and deep notch.

42) Occipital shield:
    0. Convex and forming an angle of about 40? with the longitudinal axis of the rostrum.
    1. As state 0 with an angle of about 60?
    2. Flat or concave forming an angle of about 90?

3. Flat or concave forming an angle distinctly greater than 90?

43) Falciform process of the squamosal:
   0. Contacting the corresponding pterygoid.
   1. Forming a thin plate not contacting the pterygoid.
   2. Reduced to a simple peg or absent.

44) Lateral margin of the supraorbital process of the maxilla:
   0. Dorsoventrally thin.
   1. Significantly dorsoventrally thickened, making a subvertical wall

45) Postorbital process of the frontal:
   0. Moderately posteroventrally extended.
   1. Much ventrally extended.

46) Height of temporal fossa:
   0. Dorsal margin at top of skull or somewhat lower.
   1. Much lower, temporal fossa making less than half the skull height.

47) Contact between jugal and zygomatic process:
   0. Anteroposteriorly long contact.
   1. Proportionally short, more rounded contact.
   2. No contact

48) Length of the zygomatic process.
   0. Ratio between length of the process and bizygomatic width of skull > 0.35
   1. Ratio < 0.35

49) Narrow groove in ventral surface of squamosal:
   0. Absent or shallow and poorly delineated
   1. Present

50) Long axis of the skull:
   0. Roughly parallel to the long axis of the body.
   1. Projected ventrally.
   2. Projected dorsally.

51) Upper tooth row (*ordered*):
   0. Deep alveoli with interalveolar septa well developed.
   1. Deep alveoli with rudimentary septa.

2. Shallow alveolar groove without septa

52) Premaxillary teeth (*ordered*):
   0. Present.
   1. Absent

53) Size of teeth:
   0. < 5%
   1. > 5%

54) Dental enamel 1(*ordered*):
   0. Present.
   1. Absent.

55) Number of mandibular teeth:
   0. 11
   1. 12-14
   2. > 14

56) Labiolingual compression of the posterior lower teeth:
   0. Strong
   1. Weak or absent.

57) Anteroposterior level of last upper alveolus
   0. Posterior to antorbital process
   1. At level of antorbital notch or slightly anterior
   2. Distinctly anterior to the notch

58) Ventral position of the mandibular condyle:
   0. well-developed angular process
   1. Ventral position and an angular process low or absent.

59) Curvature of mandible in lateral view
   0. absent or reduced, ventral margin roughly rectilinear or rising moderately anterodorsally.
   1. conspicuous, ventral margin distinctly convex rising both posterodorsally and anterodorsally
   2. present, ventral margin concave

60) Symphyseal angle on the mandibles
   0. < 35?

1. 35?-55?
2. > 55?

61) Dorsal process of the periotic
    0. dorsally extended and anteroposteriorly long
    1. anteroposteriorly shorter, but dorsally extended beyond the medial margin of the internal acoustic meatus.
    2. dorsally short.

62) Posteromedial outline of the pars cochlearis in dorsal view:
    0. Angular
    1. Flattened, barely convex, and roughly continuous with posterior margin of superior process.

63) Anterior bullar facet of the periotic
    0. Very anteroposteriorly elongated
    1. Reduced
    2. Absent or very small

64) Posterior extension of the posterior process:
    0. Ventrally oriented
    1. Parallel to the horizontal plane of the bone and not ventrally orientated.

65) Accessory ossicle of the tympanic bulla:
    0. Absent or small.
    1. Enlarged and partially fused with the anterior process of the periotic
    2. Present and partially fused with the anterior process.

66) Involucrum of the tympanic bulla
    0. Absent
    1. Present

67) Notch in the anterior margin of the basihyal
    0. Wide and shallow notch
    1. Narrow and deep notch
    2. No notch, rectilinear or convex anterior margin.

68) Lateral margin of atlas:
    0. Roughly rectilinear or laterally concave
    1. Convex, with laterally pointed transverse process at mid-height of the bone.

- **Supplementary Table 1: Measurements of *Scaphokogia huachafo* MUSM 972**

Dimensions	MUSM 972
Transversal longitudinal length	701
Condylobasal length	632
Length of the rostrum	415
Width of rostrum at base	182
Width of rostrum at midlength	136
Width of premaxillae at midlength of the rostrum	92
Heigth of rostrum at base	204
Heigth of rostrum at midlength	163
Heigth of rostrum at end	87
Distance from tip of rostrum to left external nare	436
Distance from tip of rostrum to right external nare	482
Maximum width of the external right bony nare	24
Maximum width of the external left bony nare	42
Greatest preorbital width (width across preorbital processes)	286e
Greatest postorbital width (width across postorbital processes)	321e
Maximum width of external nares	68

(e= estimated)

- **Supplementary Table 2: List of Taxa Featured in the morphometric analyses**

Taxon	Collection ID	Source
Acrophyseter robustus	MUSM 1399	https://doi.org/10.5852/geodiversitas2023v45a22_s3
Ankylorhiza tiedemani	CCNHM 103	https://www.phenome10k.org/ankylorhiza-tiedemani
Berardius bairdii	NHMUK 1954.9.21.1	https://www.phenome10k.org/berardius-bairdii
Cozzuoliphyseter rionegrensis	MLP 62-XII-19-1	https://sketchfab.com/3d-models/cozzuoliphyseter-rionegrensis-mlp-62-xii-19-1-24812bd42ec14b0b89a14e6ff206ed7e
Diaphorocetus ortegai	MUSM 3246	https://doi.org/10.5852/geodiversitas2023v45a22_s1
Echovenator sandersi	GSM 1098	https://www.phenome10k.org/echovenator-sandersi
Inia geoffrensis	USNM 49582	https://www.phenome10k.org/inia-geoffrensis
Kentriodon pernix	USNM V 10670	https://www.phenome10k.org/kentriodon-pernix
Kogia breviceps	USNM 22015	https://www.phenome10k.org/kogia-breviceps
Koristocetus pescei	MUSM 888	This paper
Livyatan melvillei	MUSM 1676	https://www.phenome10k.org/livyatan-melvillei
Monodon monoceros	USNM 267959	https://www.phenome10k.org/monodon-monoceros
Odobenocetops peruvianus	SMNK PAL 2491	https://www.phenome10k.org/odobenocetops-peruvianus
Orcinus orca	USNM 11980	https://www.phenome10k.org/orcinus-orca
Pebanista yacuruna	MUSM 4017	https://morphomuseum.com/articles/view/221
Phocoena	NHMUK 1900.5.7.29	https://www.phenome10k.org/phocoena-spinipinnis
Phocoenoides	USNM 238083	https://www.phenome10k.org/phocoenoides-dalli-3
Physeter macrocephalus	NHMUK 2007.1	https://www.phenome10k.org/physeter-macrocephalus
Platanista	USNM 172409	https://www.phenome10k.org/platanista-gangetica-2
Pomatodelphis sp.	CMM V3915	https://www.phenome10k.org/pomatodelphis
Pontoporia blainvillei	USNM 482727	https://www.phenome10k.org/pontoporia-blainvillei-4
Prosqualodon davidis	USNM PAL 467596	https://www.phenome10k.org/prosqualodon-davidis-cast
Raphicetus valenciae	MUSM 2543	https://doi.org/10.5852/geodiversitas2023v45a22_s2
Scaphokogia cochlearis	MUSM 1988	This paper
Scaphokogia huachafo	MUSM 972	This paper
Scaphokogia totajpe	MUSM 973	This paper
Simocetus rayi	USNM PAL 256517	https://www.phenome10k.org/simocetus-rayi
Stenella attenuata	NHMUK 1966.11.18.5	https://www.phenome10k.org/stenella-attenuata
Tasmacetus sheperdi	USNM 484878	https://www.phenome10k.org/tasmacetus-shepherdi
Tursiops truncatus	SDNHM 23798	https://www.phenome10k.org/tursiops-truncatus-2
Ziphius cavirostris	NHMUK 2006.15	https://www.phenome10k.org/ziphius-cavirostris

- **Supplemental References**

1. Celma, C. D. *et al.* Stratigraphic framework of the late Miocene to Pliocene Pisco Formation at Cerro Colorado (Ica Desert, Peru). *Journal of Maps* **12**, 515–529 (2016).
2. Celma, C. D. *et al.* Intraformational unconformities as a record of late miocene eustatic falls of sea level in the pisco formation (Southern Peru). *Journal of Maps* **14**, 607–619 (2018).
3. Ochoa, D. *et al.* Revisiting the Late Neogene chronostratigraphic and paleontological record of the Pisco Basin (Peru): Implications on the onset and evolution of modern marine-coastal Humboldt Current ecosystems. *International Journal of Earth Sciences* (2021).
4. Brand, L. R., Urbina, M., Chadwick, A. V., DeVries, T. J. & Esperante, R. A high resolution stratigraphic framework for the remarkable fossil cetacean assemblage of the Miocene/Pliocene Pisco Formation, Peru. *Journal of South American Earth Sciences* **31**, 414–425 (2011).
5. DeVries, T. J. Oligocene deposition and Cenozoic sequence boundaries in the Pisco Basin (Peru). *Journal of South American Earth Sciences* **11**, 217–231 (1998).
6. Muizon, C. de & DeVries, T. J. Geology and paleontology of late Cenozoic marine deposits in the Sacaco area (Peru). *Geologische Rundschau* **74**, 547–563 (1985).
7. Esperante, R., Brand, L. R., Nick, K. E., Poma, O. & Urbina, M. Exceptional occurrence of fossil baleen in shallow marine sediments of the Neogene Pisco Formation, Southern Peru. *Palaeogeography, Palaeoclimatology, Palaeoecology* **257**, 344–360 (2008).
8. Esperante, R., Brand, L. R., Chadwick, A. V. & Poma, O. Taphonomy and paleoenvironmental conditions of deposition of fossil whales in the diatomaceous sediments of the Miocene/Pliocene Pisco Formation, southern Peru-A new fossil-lagerstätte. *Palaeogeography, Palaeoclimatology, Palaeoecology* **417**, 337–370 (2015).
9. Gioncada, A. *et al.* Inside baleen: Exceptional microstructure preservation in a late Miocene whale skeleton from Peru. *Geology* **44**, 839–842 (2016).
10. Benites-Palomino, A., Vélez-Juarbe, J., Salas-Gismondi, R. & Urbina, M. *Scaphokogia totajpe*, sp. nov., a new bulky-faced pygmy sperm whale (Kogiidae) from the late Miocene of Peru. *Journal of Vertebrate Paleontology* **39**, e1728538 (2020).
11. Velez-Juarbe, J., Wood, A. R., De Gracia, C. & Hendy, A. J. W. Evolutionary patterns among living and fossil kogiid sperm whales: Evidence from the neogene of Central America. *PLoS ONE* **10**, 1–29 (2015).
12. Benites-Palomino, A. *et al.* Sperm whales (Physeteroidea) from the Pisco Formation, Peru, and their trophic role as fat sources for late Miocene sharks. *Proceedings of the Royal Society B* **289**, (2022).
13. Lambert, O., Bianucci, G. & Beatty, B. L. Bony outgrowths on the jaws of an extinct sperm whale support macroraptorial feeding in several stem physeteroids. *Naturwissenschaften* **101**, 517–521 (2014).
14. Marino, L., Sudheimer, K., Pabst, D. A., McLellan, W. A. & Johnson, J. I. Magnetic resonance images of the brain of a dwarf sperm whale (Kogia simus). *Journal of Anatomy* **203**, 57–76 (2003).
