# Supplementary material for: Extreme cranial asymmetry in extinct benthic feeding sperm whales
Source: Commun Biol. 2026 Nov 9;9:1406. doi: 10.1038/s42003-026-11074-2 (PMC13748650; doi:10.1038/s42003-026-11074-2)
Supplement: Supplementary file 3 [file 42003_2026_11074_MOESM3_ESM.pdf]

Description of Additional Supplementary Files

Supplementary Data 1- Landmarks were digitized using MorphoDig50 and comprised a total of 37 points (Supplementary Data)

Supplementary Data 2- These measurements were incorporated into the dataset of Okamura and Fujiwara, expanding the dataset to include 31 species of cetaceans (Supplementary Data 2)
