# Supplementary material for: Extreme cranial asymmetry in extinct benthic feeding sperm whales
Source: Commun Biol. 2026 Nov 9;9:1406. doi: 10.1038/s42003-026-11074-2 (PMC13748650; doi:10.1038/s42003-026-11074-2)
Supplement: Supplementary file 5 [file 42003_2026_11074_MOESM4_ESM.zip › Asymmetry/Whole Dataset/base 1.pdf]

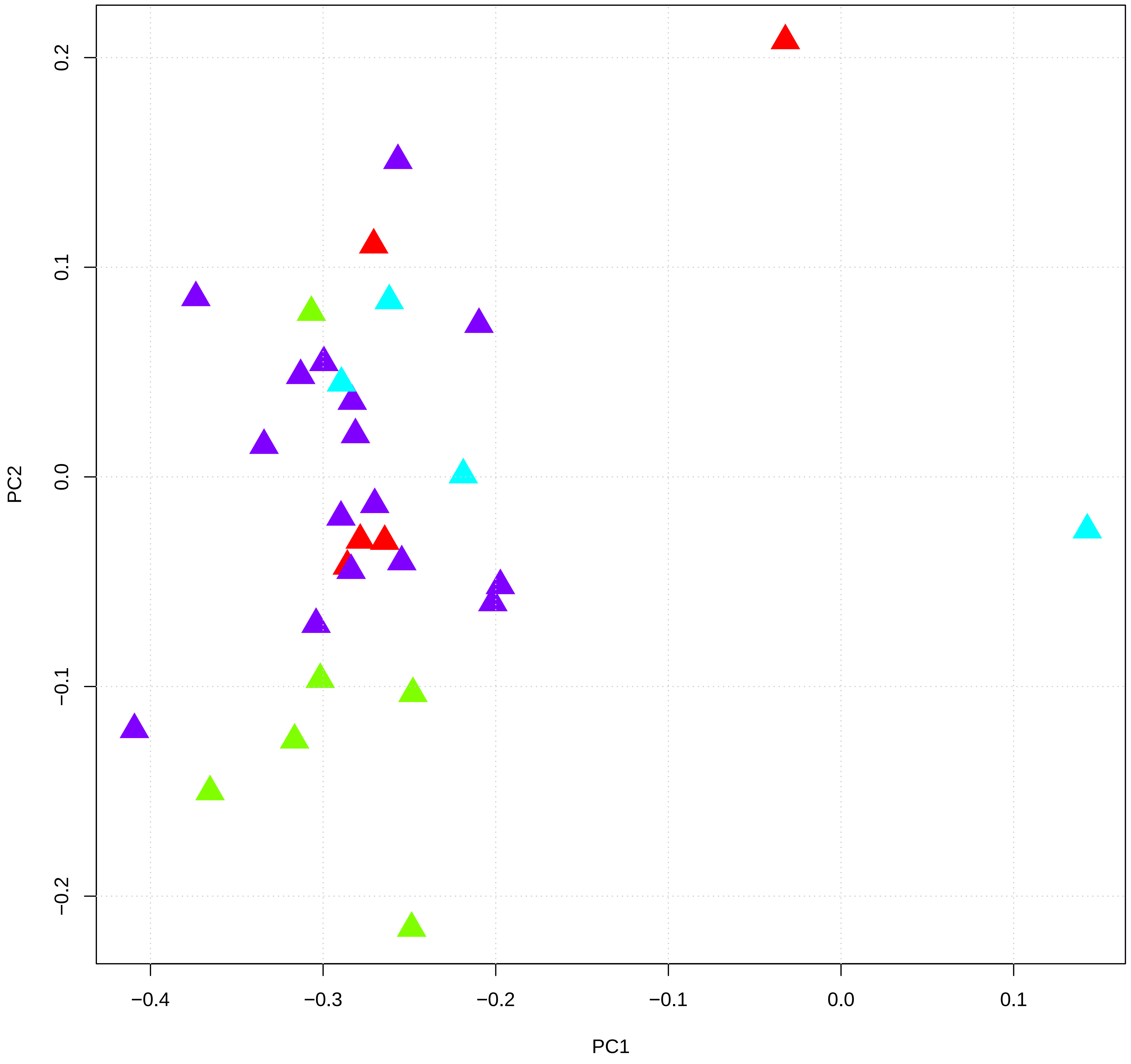
0.2
0.1
0.0
−0.1
−0.2
PC2
−0.4
−0.3
−0.2
−0.1
0.0
0.1
PC1
