# Supplementary material for: Extreme cranial asymmetry in extinct benthic feeding sperm whales
Source: Commun Biol. 2026 Nov 9;9:1406. doi: 10.1038/s42003-026-11074-2 (PMC13748650; doi:10.1038/s42003-026-11074-2)
Supplement: Supplementary file 5 [file 42003_2026_11074_MOESM4_ESM.zip › Asymmetry/Whole Dataset/Rplot.pdf]

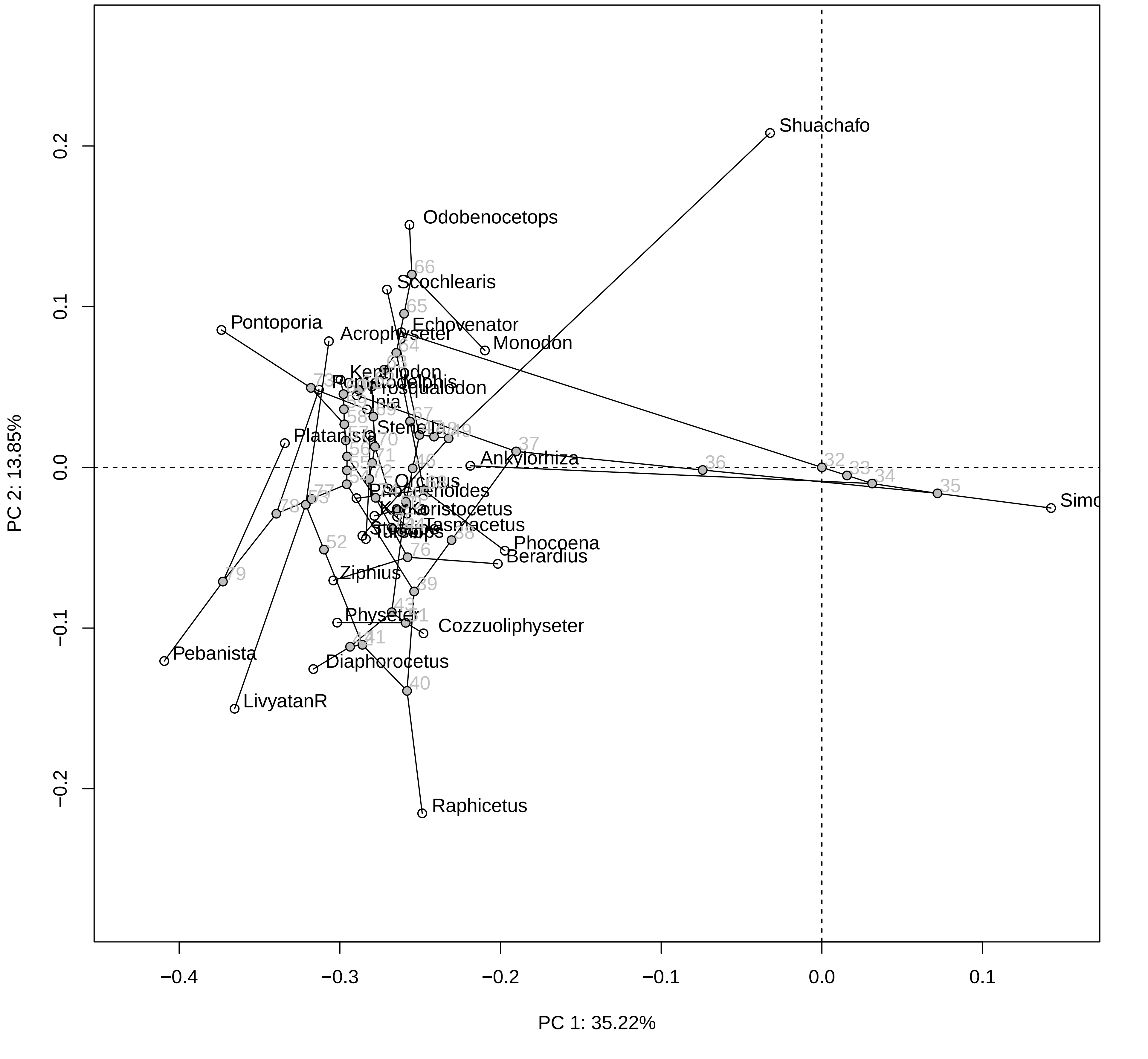

PC 1: 35.22%
PC 2: 13.85%
Shuachafo
Odobenocetops
Scochlearis
Pontoporia
Acrophyseter
Echovenator
Monodon
Kentriodon
Prosqualodon
Inia
Platanista
Ankylorhiza
Simo
Orcinus
Phocoenoides
Tasmacetus
Phocoena
Berardius
Ziphius
Physeter
Cozzuoliphyseter
Pebanista
Diaphorocetus
LivyatanR
Raphicetus
